# How effective is a powered toothbrush as compared to a manual toothbrush? A systematic review and meta‐analysis of single brushing exercises

**DOI:** 10.1111/idh.12401

**Published:** 2019-07-23

**Authors:** Therese A. Elkerbout, Dagmar E. Slot, N. A. Martijn Rosema, G. A. Van der Weijden

**Affiliations:** ^1^ Department of Periodontology, Academic Centre for Dentistry Amsterdam (ACTA) University of Amsterdam and Vrije Universiteit Amsterdam Amsterdam The Netherlands

**Keywords:** dental plaque, manual toothbrush, powered toothbrush, single brushing exercise, systematic review

## Abstract

**Objectives:**

In adult participants, what is, following a single brushing exercise, the efficacy of a powered toothbrush (PTB) as compared to a manual toothbrush (MTB) on plaque removal?

**Methods:**

MEDLINE‐PubMed and Cochrane‐CENTRAL were searched from inception to February 2019. The inclusion criteria were (randomized) controlled clinical trials conducted in human subjects ≥18 years of age, in good general health and without periodontitis, orthodontic treatment, implants and/or removable prosthesis. Papers evaluating a PTB compared with a MTB in a single brushing exercise were included. When plaque scores were assessed according to the Quigley‐Hein plaque index (Q&HPI) or the Rustogi modified Navy plaque index (RMNPI). From the eligible studies, data were extracted. A meta‐analysis and subanalysis for brands and mode of action being oscillating‐rotating (OR) and side‐to‐side (SS) were performed when feasible.

**Results:**

Independent screening of 3450 unique papers resulted in 17 eligible publications presenting 36 comparisons. In total, 28 comparisons assessed toothbrushing efficacy according to the Q&HPI and eight comparisons used the RMNPI. Results showed a significant effect in favour of the PTB. The difference of Means (DiffM) was −0.14 (*P* < 0.001; 95%CI [−0.19; −0.09]) for the Q&HPI and −0.10 (*P* < 0.001; 95%CI [−0.14; −0.06]) for the RMNPI, respectively. The subanalysis on the OR mode of action showed a DiffM −0.16 (*P* < 0.001; 95%CI [−0.22, −0.10]) for the Q&HPI. For the SS mode of action using RMNPI, the DiffM showed −0.10 (*P* < 0.001; 95%CI [−0.15; −0.05]). The subanalysis for brands showed for the P&G OR PTB using the Q&HPI a DiffM of −0.15 (*P* < 0.001; 95%CI [−0.22; −0.08]) and the Colgate SS for RMNPI showed a DiffM of −0.15 (*P* < 0.001; 95%CI [−0.18; −0.12]).

**Conclusion:**

There is moderate certainty that the PTB was more effective than the MTB with respect to plaque removal following a single brushing exercise independent of the plaque index scale that was used.

## 1 INTRODUCTION

It is well established that natural oral self‐cleaning mechanisms have no significant effect on dental plaque formation. Therefore, active removal of plaque at regular intervals is necessary.^1^ Dental plaque leads to gingivitis and can eventually turn into chronic periodontitis.^2^ Therefore, adequate oral hygiene is an essential habit for maintaining oral health.^3^

Currently, there are numerous toothbrushes available on the market. The manual toothbrush (MTB) is a simple device which is widely accepted and affordable to most people.^1^ Powered toothbrushes (PTB) have been around since the 1940s. Improvements have resulted in various types of PTBs with different power supplies and different modes of action.^4^ In 1964, Ash^5^ wrote: “Although power toothbrushes are not particularly recent in origin, advanced designs, intensive promotion and widespread use of many types and manufacturers have stimulated considerable interest and research into their safety and effectiveness.” This introductory statement remains valid almost 55 years later. The number of marketed products increases, and the volume of published clinical research data pertaining to the efficacy of these new designs also continues to expand.^6^ Whether powered brushing is superior to manual brushing has for long been a subject to controversy, as studies have demonstrated conflicting results.^7^ However, the PTB has become an established alternative to the MTB.^8^ The Cochrane Collaboration showed that the PTB is more effective in the reduction of plaque and gingivitis. This is based on studies with an evaluation time of 3 months or longer.^8^

Single brushing exercise studies are considered to provide limited information since they do not take into account the benefits of gingival health.^9^ Nevertheless, they are appropriate for assessing plaque removal, as they facilitate the control of confounding variables such as patient compliance.^10^ Two previous published systematic reviews (SR) have determined the efficacy on plaque removal, following a single brushing exercise, on plaque removal of MTB and PTB separately. A head‐to‐head comparison with a SR approach of studies evaluating a PTB vs a MTB with a single brushing model is lacking. Collective evidence would help to guide the dental care professionals in making a well‐considered recommendation for optimal plaque removal. Therefore, the purpose of this study was to review the effect on plaque removal of a PTB compared to a MTB following a single brushing exercise.

## 2 MATERIAL AND METHODS

This SR was prepared and described in accordance with the Cochrane Handbook for Systematic Reviews of Interventions and the guidelines of Transparent Reporting of Systematic Reviews and Meta‐analysis.^11^, ^12^, ^13^ The protocol that details the review method was developed a priori following an initial discussion among the members of the research team.

### 2.1 Focused question

In adult participants, what is, following a single brushing exercise, the efficacy of a PTB as compared to a MTB on plaque removal?

### 2.2 Definition of a powered toothbrush

In the dental literature, “electric” and “powered” are used interchangeably for identical toothbrushes. It may be described in general as a powered device that consists of a handle having an electromotor which converts electricity into a mechanical action that is transferred to a shaft that propels the brush‐head.^14^ A large variety of PTBs are available to the consumer. For the purpose of this review, only toothbrushes with rechargeable batteries were included. Brushes containing a normal battery to provide an electric current, those that do not have a moving brush‐head or those using a “switched off” mode, were not considered.^14^

### 2.3 Search strategy

A structured search strategy was designed to retrieve all relevant studies that evaluated the efficacy of a single brushing exercise in adults using either a PTB or a MTB. The National Library of Medicine, Washington, DC (MEDLINE‐PubMed) and the Cochrane Central Register of Controlled Trials (CENTRAL) were searched from inception to February 2019 for appropriate papers that evaluated the effect on dental plaque in a single brushing exercise in healthy adults. The reference lists of the included studies were hand‐searched to identify additional potentially relevant studies. For details regarding the search terms used, see Table 1.

**Table 1:** Search terms used for MEDLINE‐PubMed and Cochrane‐CENTRAL. The search strategy was customized according to the database being searched

The following strategy was used in the search: {(<intervention AND outcome>)} {<[(MeSH terms) Toothbrushing OR (text words) toothbrush OR toothbrushing OR toothbrush*> **AND** <(MeSH terms) dental plaque OR dental plaque index OR dental deposits OR [text words] plaque OR dental plaque OR plaque removal OR plaque index OR dental plaque removal OR dental deposit* OR dental deposits* OR dental deposit OR dental deposits>}

Note

The asterisk (*) was used as a truncation symbol.

### 2.4 Screening and selection

Titles and abstracts from the studies obtained by the searches were independently screened by two reviewers (TAE, NAMR) to select studies that potentially met the inclusion criteria. Only papers in the English language were accepted. Based on the title and abstract, the full‐text versions of potentially relevant papers were obtained. These were categorized (TAE, DES) as definitely eligible, definitely not eligible or questionable. Disagreements concerning eligibility were resolved by consensus or if disagreement persisted, by arbitration through a fourth reviewer (GAW). The papers that fulfilled all of the inclusion criteria were processed for data extraction.

The inclusion criteria were as follows:

- Randomized controlled clinical trials (RCT) or controlled clinical trials (CCT)
- Conducted in humans:
  o ≥18 years of age
  o In good general health (no systemic disorder or pregnant)
  o No periodontitis
  o No orthodontic treatment and/or removable prosthesis
  o No dental implants
- Self‐performed brushing by the participants.
- Single‐headed MTB compared to single‐headed rechargeable PTB
- Full‐mouth plaque scores assessed according to one or more plaque indices of interest or its modification:
  o Quigley and Hein plaque index (Q&HPI^15^ or the Turesky 16 modification assessed at two sites per tooth or the Lobene^17^ modification assessed at six sites per tooth).
  o Navy plaque index^18^ or Rustogi modified Navy plaque index (RMNPI).^19^

### 2.5 Assessment of heterogeneity

Factors used to evaluate the heterogeneity of outcomes of different studies were categorized as follows: study design, subject characteristics, regimen details, mode of action, brands and plaque indices.

### 2.6 Quality assessment

Two reviewers (TAE and DES) independently scored the individual methodological qualities of the included studies using the checklist as presented in Appendix S1 according to the method described in detail by Keukenmeester et al^20^ In short, a study was classified as having a “low risk of bias” when random allocation, defined inclusion/exclusion criteria, blinding to the examiner, balanced experimental groups, identical treatment between groups (except for the intervention) and reporting of loss to follow‐up were present. Blinding to the participant was not taken into account as the participants could always see whether they used a PTB or a MTB. Studies that had five of these six criteria were considered to have a potential moderate risk of bias. If two or more of these six criteria were absent, the study was considered to have a high risk of bias.^21^

### 2.7 Data extraction

From the papers that met the selection criteria, the data were processed for analysis. If possible, the mean plaque scores for pre‐brushing, post‐brushing, change and standard deviations were independently extracted. This data extraction was performed by the three independent reviewers (TAE, NAMR and DES) using a specially designed data extraction form. Disagreement between the reviewers was resolved through discussion and consensus. If a disagreement persisted, the judgement of a fourth reviewer (GAW) was decisive. Some of the studies provided standard errors (SE) of the mean. If needed and where possible, the authors calculated standard deviation (SD) based on the sample size (SE = SD/√N). If the 95% CI, mean and sample size were provided, using Omni calculator (bib22://www.omnicalculator.com/statistics/confidence-interval),^22^ the SD was calculated. For those papers that provided insufficient data to be included in the analysis, the first and/or corresponding authors were contacted to request additional data.

### 2.8 Data analysis

Pre‐ and post‐brushing plaque scores of a single brushing exercise and the change in plaque scores are presented and ordered by the plaque index score used for the assessment. The modifications of indices were categorized by the original index. As a summary, a descriptive data presentation was used for all studies. When feasible, using mean scores and the standard deviations provided by the selected papers, a meta‐analysis (MA) was performed on plaque scores and a subanalysis on the mode of action and brand using Review Manager [(RevMan) [Computer program] Version 5.3. Copenhagen: The Nordic Cochrane Centre, The Cochrane Collaboration, 2014]. In studies consisting of multiple treatment arms and data from one particular group compared with more than one other group, the number of subjects (n) in the group was divided by the number of comparisons. A meta‐analysis was only performed if there could be two or more comparisons included.^23^ The difference of means (DiffM) between PTB and MTB was calculated using a “random or fixed effects” model where appropriate. A fixed‐effect analysis was implemented if there were fewer than four studies because the estimate of between‐study variance is poor for analysis with low numbers of studies.^11^ The formal testing for publication bias was used as proposed by Egger et al^24^ with a minimum of 10 comparisons.

### 2.9 Grading the “body of evidence”

The Grading of Recommendations Assessment, Development and Evaluation (GRADE)^25^ was used to appraise the evidence.^26^ Three reviewers (TAE, DES, GAW) rated the quality of the evidence and the strength and direction of the recommendations^27^ according to the following aspects: risk of bias, consistency of results, directness of evidence, precision and publication bias, and magnitude of the effect. Any disagreement among the three reviewers was resolved after additional discussion.

## 3 RESULTS

### 3.1 Search and selection results

**Figure 1:**
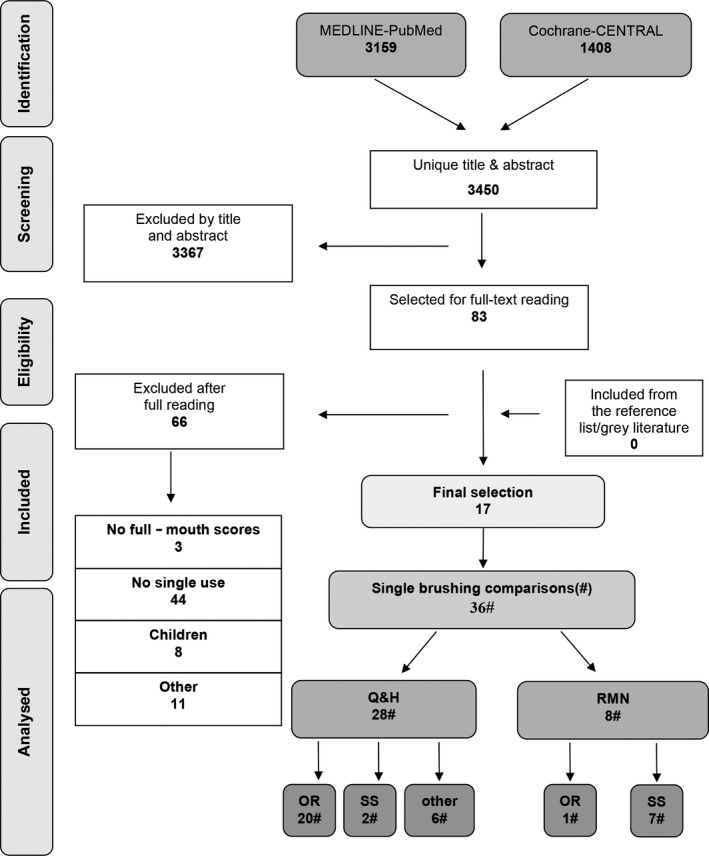
Search and selection results

Searching the MEDLINE‐PubMed and Cochrane‐CENTRAL databases resulted in 3450 unique papers (for details, see Figure 1). Screening of the titles and abstracts resulted in 83 papers, which were obtained in full text. Based on a detailed reading of these papers, 66 papers were excluded. The reasons were no full‐mouth scores, no single use or conducted among children. The other 11 studies did not fit the eligibility criteria of which one study was due to the fact that the PTB that was used was a prototype^28^ and another did a long‐term study including a single brushing exercise but unfortunately did not report the data.^29^ In total, 17 papers were selected.^30^, ^31^, ^32^, ^33^, ^34^, ^35^, ^36^, ^37^, ^38^, ^39^, ^40^, ^41^, ^42^, ^43^, ^44^, ^45^, ^46^ In various trials, more than one brush type was used to obtain data on plaque removal efficacy, resulting in 36 comparisons for inclusion in this review.

### 3.2 Assessment of heterogeneity

The selected papers showed considerable heterogeneity in study design, participant characteristics and entry criteria, period of plaque accumulation prior to the brushing experiment, used products, PTB mode of action, brands, instruction method, brushing duration and plaque indices used. Appendix S2 showed an overview of these items in the selected studies.

### 3.3 Study design

Of the 17 selected studies, 16 were randomized controlled trials (RCTs) and the Rosema et al^42^ study was a controlled clinical trial (CCT). In nine studies, a crossover design was used, and the other eight studies had a parallel design. Two studies (Khocht et al^31^ and Kulkarni et al^46^) provided also a single brushing evaluation at 4 weeks. The number of participants varied from 16 to 181, and various inclusion criteria were used. In many studies, “carious lesions” or “acute lesions” or “hard tissue lesions” were defined as exclusion criteria. These descriptions were summarized as “dental neglect.”

The method of instruction in oral hygiene practices was classified as “none” as reporting normal regimen or no instruction. Instructions according to the manufacturer or written instructions or leaflet were considered as “written.” Professional instructions by a dental care professional, video instructions or if feedback was provided are classified as “visual.” Plaque accumulation varied from 12 hours to 4 days, and brushing duration was 30 seconds till unrestricted time for self‐performed brushing (for details, see Appendix S2).

#### 3.3.1 Toothbrush and mode of action and brands for the PTBs only

In total, 21 experiments evaluated oscillating‐rotating (OR) PTBs, side‐to‐side (SS) were evaluated in nine experiments, and in six experiments, other unknown modes of action were evaluated. There were two brands with enough comparisons to do a subanalysis, so for Procter & Gamble (P&G), there were 10 experiments using the OR mode of action, and for Colgate, three experiments used the SS mode of action.

#### 3.3.2 Plaque indices

Of the 36 comparisons, 28 comparisons used the Q&HPI or a modification.^15^, ^16^ In eight comparisons, the plaque scores were assessed according to the criteria as described for the RMNPI.^18^, ^19^

### 3.4 Methodological quality assessment

The potential risk of bias was estimated based on the methodological quality aspects of the included studies as presented in the Appendix S1. Based on a summary of the proposed criteria, the potential risk of bias was estimated to be high for Kulkarni et al^46^ moderate for the Renton‐Harper et al,^34^ Pizzo et al^37^ and Kurtz et al^45^ studies and low in the remaining 13 studies.

### 3.5 Study outcomes results

The Appendix S3 presents the data as extracted per study when the Q&HPI was used and Appendix S4 when RMNPI was used. Consequently, studies are categorized by index and ordered by year of publication. Data are presented with respect to prebrushing, post‐brushing, changes in plaque scores and the absolute difference in terms of percentage of plaque scores.

#### 3.5.1 Between groups

Table 2 summarized the descriptive analysis for the statistical differences irrespective of the used PI between PTB and MTB. In addition, it shows the subanalysis based on the mode of action. Appendix S5 showed the information in more detail. Out of the 36 experiments, 22 comparisons found a significant difference in favour of the PTB in the efficacy of removing plaque after a single brushing exercise. No comparisons showed the MTB to be more effective than the PTB while eight showed parity. The descriptive subanalysis showed that for the SS mode of action, nine out of nine comparisons were significantly more effective than the MTB. For OR PTBs, this was nine out of 21 comparisons of which four did not provide sufficient statistical data.

**Table 2:** Overview of the descriptive summary of the comparisons with the number of statistical significance of PTB compared with MTB on the overall plaque scores and a subanalysis on mode of action. For details, see Appendix S5

Comparisons N = 36		PTB was more effective	MTB was more effective	No difference	Unknown	**Comparison**
Overall		22	0	8	6	
Subanalysis	PTB OR N = 21	9	0	8	4	MTB
	PTB SS N = 9	9	0	0	0	MTB
	other N = 6	4	0	0	2	MTB

Abbreviations: OR, oscillating‐rotating; SS, side‐to‐side.

### 3.6 Meta‐analysis

#### 3.6.1 Overall

Independent of the mode of action of the PTB removed more plaque as compared to the MTB. The difference of means (DiffM) for the Q&HPI was significant for the incremental change DiffM of −0.14 (*P* < 0.001; 95%CI [−0.19; −0.09]) in favour of the PTB (Table 3). The results of the change using the RMNPI showed a DiffM of −0.10 (*P* < 0.001; 95%CI [−0.14; −0.06]) (Table 4).

**Table 3:** A meta‐analysis for PTB compared with MTB at prebrushing, post‐brushing and the change in plaque scores on the Q&HPI. Presented as overall and a subanalysis of the mode of action

	Moment	#Comparisons	Model	DiffM	Test for overall		Test for heterogeneity		Online Appendix	
					95% CI	*P*‐value	*I* ^2^ value (%)	*P*‐value	Forrest plot	Funnel plot
**Overall**	Pre	27 comparisons	Random	0.04	[−0.01, 0.08]	0.09	12	0.29	S6a	S7a
	Post	20 comparisons	Random	−0.06	[−0.10, −0.02]	0.003	1	0.45	S8a	S9a
	Change	15 comparisons	Random	−0.14	[−0.19, −0.09]	<0.001	55	0.006	S10a	S11a
Subanalysis	Pre	27 OR comparisons	Random	0.04	[−0.02, 0.09]	0.18	32	0.08	S6b	S7b
	Post	20 OR comparisons	Random	−0.06	[−0.11, −0.00]	0.03	22	0.19	S8b	S9b
	Change	11 OR comparisons	Random	−0.16	[−0.22, −0.10]	<0.001	57	0.01	S10b	S11b
	Pre	2 SS comparisons	Fixed	0.06	[−0.52, 0.64]	0.85	0	0.84	S6c	NA
	Post	2 SS comparisons	Fixed	−0.06	[−0.22, 0.10]	0.45	0	0.35	S8c	NA
Subanalysis only P&G PTB	Pre	18 OR comparisons	Random	0.02	[−0.03, 0.06]	0.52	0	0.99	S6d	S7c
	Post	18 OR comparisons	Random	−0.07	[−0.12, −0.02]	0.01	15	0.28	S8d	S9c
	Change	10 OR comparisons	Random	−0.15	[−0.22, −0.08]	<0.001	60	0.008	S10c	S11c

**Table 4:** A meta‐analysis for PTB compared with MTB at prebrushing, post‐brushing and the change in plaque scores on the RMNPI. Presented as overall and a subanalysis of the mode of action

	Moment	Comparisons	Model	DiffM	Test for overall		Test for heterogeneity		Online Appendix	
					95% CI	*P*‐value	*I* ^2^ value (%)	*P*‐value	Forrest plot	Funnel plot
**Overall**	Pre	8 comparisons	Random	0.01	[0.00, 0.02]	0.02	0	1.00	S12a	NA
	Post	8 comparisons	Random	−0.08	[−0.12, −0.05]	<0.001	87	<0.001	S13a	NA
	Change	8 comparisons	Random	−0.10	[−0.14, −0.06]	<0.001	90	<0.001	S14a	NA
Subanalysis	Pre	7 SS comparisons	Random	0.01	[−0.00, 0.02]	0.06	0	1.0	S12b	NA
	Post	7 SS comparisons	Random	−0.08	[−0.12, −0.03]	0.001	87	<0.001	S13b	NA
	Change	7 SS comparisons	Random	−0.10	[−0.15, −0.05]	<0.001	91	<0.001	S14b	NA
Subanalysis only Colgate PTB	Pre	3 SS comparisons	Fixed	0.00	[−0.03, 0.04]	0.81	0	0.88	S12c	NA
	Post	3 SS comparisons	Fixed	−0.11	[−0.14, −0.08]	<0.001	79	0.009	S13c	NA
	Change	3 SS comparisons	Fixed	−0.15	[−0.18, −0.12]	<0.001	0	0.93	S14c	NA

Note

Heterogeneity was tested by the chi‐square test and the *I* ^2^ statistic. A chi‐square test resulting in a *P* < 0.1 was considered an indication of significant statistical heterogeneity. As an approximate guide for assessing the magnitude of inconsistency across studies, an *I* ^2^ statistic of 0‐40% was interpreted as potentially not important, and for a statistic above 40%, a moderate to considerable heterogeneity may be present.

Abbreviations: CI, confidence interval; DiffM, difference of means; NA, not applicable; OR, oscillating‐rotating; PI, plaque index; SS, side‐to‐side.

#### 3.6.2 Subanalysis

A subanalysis on the mode of action showed that for the OR technology using the Q&HPI a DiffM of −0.16 (*P* < 0.001; 95%CI [−0.22; −0.10]) (Table 3). For the SS technology, using the RMNPI showed a DiffM of −0.10 (*P* < 0.001; 95%CI [−0.15; −0.05]). The subanalysis for brands showed for the P&G PTB with the OR technology using the Q&HPI a DiffM of −0.15 (*P* < 0.001; 95%CI [−0.22; −0.08]). The Colgate PTB with the SS technology using the RMNPI showed a DiffM of −0.15 (*P* < 0.001; 95%CI [−0.18; −0.12]). Tables 3 and 4 show a summary of the MA outcomes. Detailed information regarding the forest plots and funnel plots can be found in the Appendices S6‐S14.

### 3.7 Evidence profile

Table 5 presents a summary of the various factors used to rate the quality of evidence and to appraise the strength and direction of recommendations according to GRADE^25^ including the level of certainty. There is a small difference in plaque removal in favour of the PTB. The single brushing design is rather direct as it does not reflect long‐term use. As the risk of bias varied from “low to high” and many studies were industry‐financed reporting bias cannot be ruled out. The strength of the recommendation was estimated to be “strong” due to the precision and rather consistent results of the plaque scores. Given the strength of this recommendation, there is a moderate rate of certainty of the beneficial effect of a PTB removing more dental plaque than a MTB.

**Table 5:** Estimated evidence profile appraisal of the strength of the recommendation, and the direction regarding the use of the PTB compared with the MTB based on a single brush exercise on the plaque removal

Determinants of the quality	Plaque score
Study design (Appendix S2)	RCT/CCT
# Studies (Figure 1) # Comparisons	# 17 # 36
Risk of bias (Appendix S1)	Low to high
Consistency (Table 2)	Rather consistent
Directness	Rather generalizable
Precision (Tables 3 and 4)	Precise
Reporting bias	Likely
Magnitude of the effect (Tables 3 and 4)	Small
Strength of the recommendation based on the quality and body of evidence	Strong
Direction of recommendation: With respect to the removal of dental plaque, there is moderate certainty to advise a PTB over a MTB	

## 4 DISCUSSION

This review selected and included studies that evaluated the efficacy of a PTB compared with a MTB following a single brushing exercise on plaque removal. The efficacy of PTBs and MTBs was compared by assessing prebrushing and post‐brushing plaque scores following a single brushing exercise. A single brushing model provides a useful indication of the plaque removal ability of a toothbrush and facilitates control of confounding variables such as compliance, frequency of toothbrushing and probably even the Hawthorne and novelty effects.^9^, ^10^, ^36^, ^47^ Most of the included studies were previously used in the reviews regarding the efficacy of the MTB^48^ or the PTB.^14^ These reviews showed that on average the plaque removing efficacy for the MTB was 42% and 46% for the PTB. Rosema et al^14^ showed that brushes with rechargeable batteries yield higher reductions in plaque scores then replaceable battery‐operated designs.^14^ It was therefore decided a priori to include only rechargeable PTBs in the present review. Terézhalmy et al^28^ was included in the SR of Rosema in 2016 as a replaceable PTB, but after critically re‐reading the paper, it was excluded in this review because in the description of the brush, it was mentioned that this was a prototype and a special rechargeable battery was used. In addition, as a result of a search update, new studies (Re et al,^43^ Gallob et al,^44^ Kurtz et al^45^ and Kulkarni et al^46^) could be included. Consequently, the present review included in total of 17 studies with 36 comparisons and observed a small but statistically significant higher level of efficacy in plaque removal in favour of the PTB. The differences in mode of action on the efficacy of a PTB are interesting. The overall data in the MA include all modes of action. In the subanalysis, only OR and SS could be taken into account. From this subanalysis, it is shown that both the OR and SS mode of action are more effective than the MTB. As for brands, the subanalysis showed that the OR P&G PTB and the SS Colgate PTB are more effective than the MTB. However, the direct comparison between OR and SS cannot be deduced from the outcome of this review.

### 4.1 Plaque indices

The RMNPI^18^ and the Q&HPI^15^ and their modifications are the two indices most commonly used for assessing plaque removal efficacy with toothbrushes. Although these indices score plaque in different ways, there appears to be a strong positive correlation between them.^49^ The MA showed that the PTB is more effective than the MTB, independent of the overall plaque score used (Appendices S6a, S8a, S10a, S12a, S13a, S14a). Sicilia et al^50^ proposed some common minimum indexes which should be included in a study. From the data of their review, they deduced that the Q&HPI^15^ would be the most suitable. It is important for further SRs that clinical trials employ common indexes for the quantitative analysis.^50^ The choice of the index however appears to be based on an industry policy or a research facility opportunity. As a result, the manufacturers producing different modes of action PTB, use different plaque indices to evaluate the efficacy. In this review, most PTBs with the OR mode of action assessed the Q&HPI. In contrary, most PTB with the SS mode of action assessed the RMNPI. This may result in a reporting bias.

### 4.2 Publication bias and risk of bias

The analysis of funnel plots provides a useful test for the possible presence of bias in the MA. The capacity to detect bias will be limited when MA is based on a few number of small trials due to the fact that the methods for detecting publication bias relate effect size to sample size.^8^, ^24^ Publication bias in this SR might be subjectively inferred since the funnel shape is asymmetrical (Appendices S7a‐c, S9a‐c, S11a‐c). In the lower part of the funnel plots, studies are missing and the assumption is that these showed no beneficial effect and were therefore not published.^24^, ^51^, ^52^ Publication bias can therefore not be ruled out.

In 14 studies, the instructions were given according to what the manufacturer did advice. Only three studies gave written instructions to the users of the PTB but no instructions to the MTB users.^34^, ^37^, ^45^ This aspect can potentially introduce a bias as emphasis on the brushing method in the form of a written instruction can change the individual brushing skills. This may enhance the effect of the PTB over the MTB which will have an impact on the overall outcome. However, this was not apparent when a sensitivity analysis was performed. It does have an effect on the estimated potential risk of bias because the treatment was not identical for both the interventions.

### 4.3 Familiarization phase and learning effect

Glavind et al^53^ have suggested that the mere participation of a group in a preventive programme may in itself improve the level of oral hygiene. Lazarescu et al (2003)^29^ evaluated the effect in efficient handling of a manual and PTB over an 18‐week period. There appeared to be a significant learning effect that was more pronounced with the electric toothbrush in first‐time users. Also, Van der Weijden et al (2001)^54^ observed in a study with power toothbrushes a “learning effect” during the familiarization phase.

Five included studies^32^, ^34^, ^35^, ^38^, ^40^ used a familiarization phase before the single brushing experiment. We performed a subanalysis to investigate the impact on plaque removal efficacy of such a period for the participants to become familiar with their assigned product. Statistical analysis (data not shown) demonstrated that there was no difference between studies that did, and those studies that did not include a familiarization phase, neither on prebrushing nor on post‐brushing scores. This rather disappointing observation may be explained by the outcome of the study by Van Leeuwen et al.^55^ They found that a single oral hygiene instruction and 3 weeks of home use did not significantly change the plaque scores from baseline.

### 4.4 Indication for clinical practitioners

Both the use of PTBs or MTBs has been reported to have positive effects on plaque and gingivitis reduction in many RCTs. Therefore, recommending the use of a toothbrush to patients is supported by evidence.^56^, ^57^ Many factors may be of influence for the effectiveness of toothbrushes including filament arrangement, filament orientation and angulation, filament size, filament shape and filament flexibility, brush‐head size and brush‐head shape. For PTBs, in particular, this may also be the brushing speed^58^ as well as the presence or absence and characteristics of a timer.^4^ The Cochrane Collaboration review concluded that the PTB reduces plaque and gingivitis more than a MTB both in the short and long term.^8^ Based on the present review, it is justifiable to state that independent of the mode of action a PTB is more effective in reducing plaque as compared to a MTB.

## 5 LIMITATIONS

The English language restriction could have introduced a language bias. However, over the years, the extent and effects of such a possible bias may have diminished because of the shift towards publication in English.^11^

Blinding for the participant was not possible due to the fact that they see and experience whether they use a PTB or a MTB which cannot be excluded. For the examiners, blinding to the toothbrush is feasible. Blinding the examiner to the single brushing exercise deserves special attention mainly regarding the sound. Some of the studies have reported on this particular aspect.^35^, ^40^, ^45^

Only full publications were taken into account. No abstracts from scientific meetings or data on file of manufacturers were sought.

## 6 CONCLUSION

There is moderate certainty that the PTB was more effective than the MTB with respect to plaque removal following a single brushing exercise independent of the plaque index score that was used.

## 7 CLINICAL RELEVANCE

### 7.1 Scientific rationale for the study

Toothbrushing is generally accepted as the most efficient oral hygiene method.

Traditionally, MTBs are used, but the last decades’ PTBs became more popular. Data from a comparison of MTB vs PTB in single brushing exercises have at present not been systematically evaluated.

### 7.2 Principle findings

PTB and MTB are both effective oral hygiene devices for removing dental plaque. There is a small but significant difference observed in plaque score reduction in favour of a PTB.

### 7.3 Practical implications

Consequently, for plaque removal in daily oral hygiene, with moderate certainty the PTB can be recommended over a MTB independent of the mode of action.

## CONFLICT OF INTEREST

The authors declare that they have no conflict of interest.

## STATEMENT OF AUTHORSHIP

All authors gave final approval and agreed to be accountable for all aspects of work ensuring integrity and accuracy. TAE contributed to design, search and selection, analysis and interpretation, drafted the manuscript, DES and NAMR contributed to conception and design, search and selection, analysis and interpretation, and critically revised the manuscript. GAW contributed to conception and design, analysis and interpretation, and critically revised the manuscript.
