# Supplementary material for: How effective is a powered toothbrush as compared to a manual toothbrush? A systematic review and meta‐analysis of single brushing exercises
Source: Int J Dent Hyg. 2019 Jul 23;18(1):17–26. doi: 10.1111/idh.12401 (PMC7004084; doi:10.1111/idh.12401)

How effective is a powered toothbrush as compared to a manual toothbrush?

-A Systematic Review and meta-analysis-

T.A. Elkerbout *

D.E. Slot *

N.A.M. Rosema*

G.A. Van der Weijden *

Online appendices

Online supporting information legends

Appendix S1

Methodological quality and potential risk of bias scores of the individual included studies

Appendix S2

Overview of the studies processed for data extraction

Appendix S3

Studies using the Turesky (Q&H) (Turesky et al. 1970) modification of the Quigley & Hein (Q&H 1962) PI

Appendix S4

Studies using the Navy plaque index (Elliott et al 1972) or Rustogi modified Navy (Rustogi et al 1992) PI

Appendix S5

A descriptive summary of statistical significance of PTB compared to MTB

Appendix S6a

Forest plot of the overall analysis for PTB compared to MTB at pre-brushing in plaque scores on the Q&HPI

Appendix S6b

Forest plot of the random effects subanalysis for the OR mode of action for PTB compared to MTB at pre-brushing in plaque scores on the Q&HPI

Appendix S6c

Forest plot of the fixed effects subanalysis for the SS mode of action for PTB compared to MTB at pre-brushing in plaque scores on the Q&HPI

Appendix S6d

Forest plot of the random effects subanalysis for the OR mode of action by product (P&G) for PTB compared to MTB at pre-brushing in plaque scores on the Q&HPI

Appendix S7a

Funnel plot for the overall analysis for PTB compared to MTB at pre-brushing in plaque scores on the Q&HPI

Appendix S7b

Funnel plot for the random effects subanalysis for the OR Mode of Action for PTB compared to MTB at pre-brushing in plaque scores on the Q&HPI

Appendix S7c

Funnel plot for the random effects subanalysis for the OR mode of action by product (P&G) for PTB compared to MTB at pre-brushing in plaque scores on the Q&HPI

Appendix S8a

Forest plot of the overall analysis for PTB compared to MTB at post-brushing in plaque scores on the Q&HPI

Appendix S8b

Forest plot of the random effects subanalysis for the OR Mode of Action for PTB compared to MTB at post-brushing in plaque scores on the Q&HPI

Appendix S8c

Forest plot of the fixed subanalysis for the SS mode of action for PTB compared to MTB at the post-brushing in plaque scores on the Q&HPI

Appendix S8d

Forest plot of the random effects subanalysis for the OR mode of action by product (P&G) for PTB compared to MTB at post-brushing in plaque scores on the Q&HPI

Appendix S9a

Funnel plot for the overall analysis for the mode of action for PTB compared to MTB at post-brushing in plaque scores on the Q&HPI

Appendix S9b

Funnel plot for the random effects subanalysis for the OR mode of action for PTB compared to MTB at post-brushing in plaque scores on the Q&HPI

Appendix S9c

Funnel plot of the random effects subanalysis for the OR mode of action by product (P&G) for PTB compared to MTB at post-brushing in plaque scores on the Q&HPI

Appendix S10a

Forest plot of the overall analysis for PTB compared to MTB at the change in plaque scores on the Q&HPI

Appendix S10b

Forest plot of the random effects subanalysis for the OR mode of action for PTB compared to MTB at the change in plaque scores on the Q&HPI

Appendix S10c

Forest plot of the random effects subanalysis for the OR mode of action by product (P&G) for PTB compared to MTB at the change in plaque scores on the Q&HPI

Appendix S11a

Funnel plot of the overall analysis for PTB compared to MTB at the change in plaque scores on the Q&HPI

Appendix S11b

Funnel plot of the random effects subanalysis for the OR mode of action for PTB compared to MTB at the change in plaque scores on the Q&HPI

Appendix S11c

Funnel plot of the random effects subanalysis for the OR mode of action by product (P&G) for PTB compared to MTB at the change in plaque scores on the Q&HPI

Appendix S12a

Forest plot of the overall analysis for PTB compared to MTB at pre-brushing in plaque scores on the RMNPI

Appendix S12b

Forest plot of the random effects subanalysis for the SS mode of action for PTB compared to MTB at pre-brushing in plaque scores on the RMNPI

Appendix S12c

Forest plot of the random effects subanalysis for the SS mode of action by product (Colgate) for PTB compared to MTB at pre-brushing in plaque scores on the RMNPI

Appendix S13a

Forest plot of the overall analysis for PTB compared to MTB at post-brushing in plaque scores on the RMNPI

Appendix S13b

Forest plot of the random effects subanalysis for the SS mode of action for PTB compared to MTB at post-brushing in plaque scores on the RMNPI

Appendix S13c

Forest plot of the random effects subanalysis for the SS mode of action by product (Colgate) for PTB compared to MTB at post-brushing in plaque scores on the RMNPI

Appendix S14a

Forest plot of the overall analysis for PTB compared to MTB at the change in plaque scores on the RMNPI

Appendix S14b

Forest plot of the random effects subanalysis for the SS mode of action for PTB compared to MTB at the change in plaque scores on the RMNPI

Appendix S14c

Forest plot of the random effects subanalysis for the SS mode of action by product (Colgate) for PTB compared to MTB at the change in plaque scores on the RMNPI

Appendix S1

Methodological quality and potential risk of bias scores of the individual included studies

	Study / Quality criteria	I	II	III	IV	V	VI	VII	VIII	IV	X	XI	XII	XIII	XIV	XV	XVI	XVII
		Killoy (1989)	Khocht (1992)	Van der Weijden (1996)	Heasman (1999)	Renton-Harper (2001)	Moritis (2002)	Sharma (2006)	Pizzo (2010)	Sharma (2011)	Nathoo (2012)	Klukowska (2012)	Nathoo 2014)	Rosema (2014)	Gallob (2015)	Re (2015)	Kurtz (2016)	Kulkarni (2018)
	Study design	parallel	parallel	cross-over	parallel	cross-over	cross-over	parallel	cross-over	cross-over	parallel	cross-over	parallel	cross sectional	parallel	cross-over	cross-over	parallel
internal validity	Random allocation *	+	+	+	+	+	+	+	+	+	+	+	+	NA	+	+	+	?
	Allocation concealment	?	?	?	?	?	?	?	+	+	?	+	?	-	+	?	+	?
	Blinded to product *	NA	NA	NA	NA	NA	NA	NA	NA	NA	NA	NA	NA	NA	NA	NA	NA	NA
	Blinded to examiner *	+	+	+	+	+	+	+	+	+	+	+	+	+	+	+	+	?
	Blinding during statistical analysis	?	?	?	?	?	?	?	?	?	?	?	?	?	?	?	?	?
	Balanced experimental groups *	+	+	+	+	+	+	+	+	+	+	+	+	+	+	+	+	+
	Reported loss to follow up*	+	+	+	+	+	+	+	+	+	+	+	+	+	+	+	+	+
	# (%) of drop-outs	0	1 (1%)◊	0	1 (1%)◊	0	0	0	0	0	6 (7%)◊	0	0	0	1 (1%)◊	0	8 (8%)◊	0
	Treatment identical, except for intervention *	+	+	+	+	-	+	+	-	+	+	+	+	+	+	+	-	+

**External validity**	Representative population group	+	+	+	+	+	+	+	+	+	+	+	+	+	+	+	+	+
	Eligibility criteria defined *	+	+	+	+	+	+	+	+	+	+	+	+	+	+	+	+	+
**Statistical validity**	Sample size calculation and power	?	?	?	+	?	?	?	+	+	?	+	?	-	+	?	+	+
	ADA sample size $n \geq 30$[⌂⌂]	-	+	-	-	-	-	+	+	+	+	+	+	+	+	+	+	-
	Point estimates presented for the primary outcome	+	+	+	+	+	+	+	+	+	+	+	+	+	+	+	+	+
	Measures of variability presented for the primary outcome	+	+	+	+	+	+	+	+	+	+	+	+	+	+	+	+	+
	Unit of analysis	full mouth	full mouth	full mouth	full mouth	full mouth	full mouth	full mouth	full mouth	full mouth	full mouth	full mouth	full mouth	full mouth	full mouth	full mouth	full mouth	full mouth
	Include an per protocol analysis	?	?	?	?	?	?	?	?	?	?	?	?	?	?	?	?	?
	Include an intention- to-treat analysis	+	+	+	+	+	+	+	+	+	+	+	+	+	+	+	+	+
	Correction for multiple comparisons	?	?	?	?	?	?	?	?	?	?	?	?	?	?	?	?	?
	Validated measurement	+	+	+	+	+	+	+	+	+	+	+	+	+	+	+	+	+
	Calibration examiner	?	+	?	+	?	+	+	+	?	+	+	+	+	+	-	-	+
	Reproducibility data shown	-	-	-	-	-	-	-	-	-	-	-	-	-	-	-	-	-
**Authors estimated risk of bias**		Low	Low	Low	Low	moderate	Low	Low	moderate	Low	Low	Low	Low	Low	Low	Low	moderate	High

Each aspect of the score list was given a rating of ‘+’ for an informative description of the item concerned and a study design meeting the quality standard, ‘-’ for an informative description without a study design that met the quality standard, and ‘?’ for lacking or insufficient information. When random allocation, defined eligibility criteria, blinding of examiners, balanced experimental groups, identical treatment between groups (except for intervention), and report of follow-up were present, the study was classified as having a low risk of bias. When one of these seven criteria was missing, the study was considered to have a moderate potential risk of bias. When two or more of these criteria were missing, the study was considered to have a high potential risk of bias, as proposed by Van der Weijden et al. (2009). For abbreviations, see Table 2

◊ Calculated by the authors when not provided in the manuscript. ? = not specified/unclear + = yes - = no * = reporting criteria for estimation the potential risk of bias NA= not applicable > not possible for the patients

⌂⌂American Dental Association (ADA), Council on Scientific Affairs, Acceptance program Requirements; Toothbrushes (2016).

Appendix S2
Overview of the studies processed for data extraction

**Authors (year)**<br><br>**Study design**<br><br>**Plaque Index**	**# Participants baseline (end),**<br><br>**gender,**<br><br>**age (mean/range),**<br><br>**Oral Prophylaxis (OP)**	**Mode of action per group/ *brands***	**Instruction method (I),**<br><br>**Brushing duration**<br><br>**Hours of plaque accumulation**	**Participant exclusion criteria**	**Conclusions of the original authors**
Killoy et al. (1989[30]<br><br>RCT<br><br>Parallel<br><br>Q&H	24(24)<br><br>♀: ?<br>♂: ?<br><br>Mean age: ?<br>Age range: ?<br><br>?	CO-PTB<br>*(?)*<br><br>MTB<br>*(?)*	I: visual<br><br>3 minutes<br><br>?	<3 month AB<br><br><50% plaque	The CO-PTB brush was superior to the MTB in reducing supragingival plaque.
Khocht et al. (1992)[31]<br><br>RCT<br><br>Parallel<br><br>Q&H	64◊(63◊)<br><br>♀: ?<br>♂: ?<br><br>Mean age: ?<br>Age range:?<br><br>?	CO-PTB<br>*(Interplak)*<br><br>MTB<br>*(Oral-B 40)*	I:visual<br><br>un-restricted<br><br>overnight	periodontal disease<br><br>dental neglect<br><br>< 15 teeth<br><br>GI score (Löe) < 0.9<br><br>plaque score < 1.8	Each of the 3 brushes was effective in reducing plaque for every tooth surface scored. Between group analysis showed that CO-PTB was better than the MTB

Van der Weijden et al. (1996[32]<br>RCT<br>Cross over<br>Q&H	20 (20)<br>♀: ?<br>♂: ?<br>Mean age: ?<br>Age range:?<br>?	OR-PTB<br>*(Braun Plak Control)*<br>CO-PTB<br>*(Interplak)*<br>Cir-PTB<br>*(Rotadent)*<br>MTB<br>*(?)*	I: ?<br>2 minutes<br>23-25 h	periodontal disease<br>< 24 teeth<br>>25% plaque	The PTB are more effective in plaque removal than the MTB.
Heasman et al. (1999)[33]<br>RCT<br>Parallel<br>Q&H	75 (74)<br>♀: ?<br>♂: at least 10<br>Mean age: ?<br>Age range: 18-25<br>OP	OR-PTB<br>*(Philips Jordan HP 735)*<br>OR-PTB<br>*(Braun Oral-B D7)*<br>MTB<br>*(Oral-B 35 advantage)*	I:visual<br>90 seconds<br>24 h	periodontal disease<br>removable prosthesis<br>dental neglect<br>< 20 teeth	There were no significant differences for PI between groups following supervised brushing at 24h.
Renton-Harper et al. (2001)[34]<br>RCT<br>Cross over	16 (16)<br>♀: 10<br>♂: 6<br>Mean age: ?	OR-PTB<br>*(Braun Oral-B Plaque Remover D5)*<br>OR-PTB	I:<br>PTB >written<br>MTB> none<br>30 seconds	orthodontics<br>removable prosthesis<br>plaque score < 1.5 or > 2.5	The present study showed greater benefits from the use of electric brushes.

washout 2,5 days<br><br>Q&H	Age range: 23-41<br><br>OP	*(Braun Oral-B Plaque Remover D9)*<br><br>MTB<br>*(Oral-B 35)*	60 seconds<br><br>120 seconds<br><br>4 d		
Moritis et al. (2002)[35]<br><br>RCT<br><br>Cross over<br>No washout<br><br>Q&H	25 (25)<br><br>♀: 17<br>♂: 8<br><br>Mean age:45.3 (10.9)<br>Age range: ?<br><br>OP	SS-PTB<br>*(Sonicare Elite)*<br><br>MTB<br>*(Oral-B 35)*	I: written<br><br>2 minutes<br><br>12-18 h	periodontal disease<br><br>orthodontics<br><br>dental neglect<br><br>> 20 teeth<br><br>plaque score > 1.80	The SS-PTB achieved a significantly greater reduction in plaque than the MTB.
Sharma et al. (2006)[36]<br><br>RCT<br><br>Parallel<br><br>RMN	144 (144)<br><br>♀: 96<br>♂: 48<br><br>Mean age: 37.5 (10.94)<br>Age range: 19-66<br><br>No OP	SS-PTB<br>*(Sonicare Elite)*<br><br>SS-PTB<br>*(Oral-B Sonic Complete)*<br><br>SS-PTB<br>*(Waterpik Sensonic)*<br><br>MTB<br>*(Oral-B 35 Indicator)*	I: none<br><br>2 minutes<br><br>23-25 h	periodontal disease<br><br>orthodontics<br><br>removable prosthesis<br><br>dental neglect<br><br>< 20 teeth<br><br>plaque score < 0.6	Two out of the three SS-PTB compared favorably to the MTB.

Pizzo et al. (2010)[37]<br><br>RCT<br><br>Cross over<br>Washout ?<br><br>Q&H	66(66)<br><br>♀: 28<br>♂: 38<br><br>Mean age: 36.5 (10.1)<br>Age range: 18-59<br><br>OP	OR-PTB<br>*(Oral-B PC 8500)*<br><br>MTB<br>*(Oral-B Crossaction Vitalizer)*<br><br>MTB<br>*(Oral-B Indicator)*	I:<br>PTB> written<br>MTB> none<br><br>1 minute<br><br>23-25 h	periodontal disease<br><br>orthodontics<br><br>removable prosthesis<br><br>< 20 teeth.<br><br>plaque score < 2.0<br><br><3 month antimicrobial agents	The OR-PTB demonstrated to be more effective in plaque control than the 2 MTBs.
Sharma et al. (2011)[38]<br><br>RCT<br><br>Cross over<br>Washout ?<br><br>RMN	40 (40)<br><br>♀: 28<br>♂: 12<br><br>Mean age: 42.3 (11.36)<br>Age range: 20-69<br><br>?	OR-PTB<br>*(Oral-B Professional Care 1000)*<br><br>MTB<br>*(Oral-B Indicator)*	I: written<br><br>2 minutes<br><br>24 h	dental neglect<br><br>< 16 teeth	The OR-PTB with a novel brush head showed statistically significantly superior plaque reduction compared to a MTB.
Nathoo et al. (2012)[39]<br><br>RCT<br><br>Parallel<br><br>RMN	82 (76)<br><br>♀: 51<br>♂: 25<br><br>Mean age: 43<br>Age range: 18-66<br><br>?	SS-PTB<br>*(Colgate Pro Clinical A1500 Triple clean brush head)*<br><br>MTB<br>*(Oral-B Indicator)*	I: written<br><br>2 minutes<br><br>24 h	periodontal disease<br><br>orthodontics<br><br>removable prosthesis<br><br>dental neglect<br><br>< 20 teeth	The SS-PTB provides statistically significant and clinically relevant levels of efficacy in the removal of supragingival dental plaque after a single tooth brushing compared to a MTB.

				GI score (Löe) < 1.0<br>plaque score < 0.6	
Klukowska et al. (2012)[40]<br>RCT<br>Cross over washout 2-5 days<br>Q&H	36 (36)<br>♀: 31<br>♂: 5<br>Mean age: 45.6 (8.63)<br>Age range: 25-60<br>?	OR-PTB (with SS movement)<br>*(Oral-B Vitality TriZone)*<br>MTB<br>*(?)*	I: written<br>2 minutes<br>23-25 h	periodontal disease<br>orthodontics<br>removable prosthesis<br>dental neglect<br>< 16 teeth	Comparing the brushes, the SS-PTB provided a significantly superior mean whole mouth plaque reduction relative to the manual brush control.
Nathoo et al. (2014)[41]<br>RCT<br>Parallel<br>RMN	120 (120)<br>♀:71<br>♂: 49<br>Mean age: 41.4<br>Age range: 18-67<br>?	SS-PTB<br>*(Colgate Pro Clinical C200 Triple clean brush head)*<br>SS-PTB<br>*(Colgate Pro Clinical C200 Sensitive clean brush head)*<br>MTB<br>*(Oral-B indicator)*	I: written<br>2 minutes<br>24 h	periodontal disease<br>orthodontics<br>removable prosthesis<br>dental neglect<br><20 teeth<br>plaque score < 0.6<br>GI score (Löe) < 1.0	Both SS-PTB's provides statistically significant and clinically relevant levels of efficacy in the removal of supragingival dental plaque when compared to a manual flat-trim toothbrush.

Rosema et al. (2014)[42]<br><br>CCT<br><br>Cross sectional<br><br>Q&H	181 (181)<br><br>♀: 125<br>♂: 56<br><br>Mean age: 22.7<br>Age range: 18-35<br><br>?	OR-PTB<br>*(?)*<br><br>MTB<br>*(?)*	I:none<br><br>unrestricted<br><br>48h	periodontal disease<br>orthodontics<br>removable prosthesis<br>dental neglect<br>< 20 teeth<br>daily user OR-PTB	The PTB removed significantly more plaque than the MTB.
Re et al. (2015)[43]<br><br>RCT<br><br>Cross over washout 2-5 days<br><br>Q&H	40 (40)<br><br>♀: 22<br>♂: 18<br><br>Mean age: 24 (3.5)<br>Age range: 18-32<br><br>?	SS-PTB<br>*(Philips Sonicare DiamondClean 300)*<br><br>MTB<br>*(Butler Gum 409 Compact Soft)*	I: visual<br><br>2 minutes<br><br>23-25 h	periodontal disease<br>orthodontics<br>removable prosthesis<br>< 24 teeth	In subjects without any previous experience of a similar technology, the SS-PTB showed a significantly greater plaque reduction compared to the MTB.
Gallob et al. (2015)[44]<br><br>RCT<br><br>Parallel<br><br>RMN	80 (79)<br><br>♀: 58<br>♂: 21<br><br>Mean age: 49.3<br>Age range: 19-69<br><br>?	SS-PTB<br>*(Colgate ProClinical A1500)*<br><br>MTB<br>*(Oral-B Indicator)*	I: written<br><br>2 minutes<br><br>24 h	periodontal disease<br>orthodontics<br>removable prosthesis<br>dental neglect<br><20 teeth<br>plaque score ≥ 0.6	Compared to the MTB, the SS-PTB demonstrates statistically and clinically significantly greater levels of plaque removal

Kurtz et al. (2016)[45]<br><br>RCT<br><br>Cross over washout 2-5 days<br><br>Q&H	95 (87)<br><br>♀: 54<br>♂: 41<br><br>Mean age: 30.2 (11.87)<br>Age range: 18-67<br><br>?	OR-PTB<br>*(Oral-B Vitality D12)*<br><br>MTB<br>*(?)*	I:<br>PTB> written<br>MTB> none<br><br>PTB:2 minutes<br>MTB:1 minute<br><br>24h	periodontal disease<br><br>orthodontics<br><br>removable prosthesis<br><br>dental neglect<br><br>< 16 teeth	The OR-PTB removed significantly more plaque than the MTB.
Kulkarni et al. (2018)[446]<br><br>RCT<br><br>Parallel<br><br>Q&H	45<br><br>♀: 30<br>♂: 15<br><br>Mean age: ?<br>Age range: 18-25<br><br>?	OR-PTB<br>*(Oral-B 2D)*<br><br>MTB<br>*(Oral-B 40 advantage)*	I: written<br><br>2 minutes<br><br>24 h	dental neglect<br><br>< 20 teeth<br><br>never used PTB<br><br>smoking<br><br><1 month medication	All individuals within both groups showed reduction in PI.

* OR=Oscillating Rotating, SS=Side to Side, CO=Counter-Oscillation, Cir=Circular, AB=antibiotics

Appendix S3

Studies using the Q&HPI

Paper	Equation	N	Brushing mean (SD)			
			PRE	POST	DIFF	
					Absolute	%
Killoy et al.(1989)	CO-PTB	12	2.5	0.63	-1.87◊	75◊
	MTB	12	2.47	1.34	-1.13◊	46◊
Khocht et al.(1992)	CO-PTB	32 V1	2.27 (0.36)	1.52 (0.36)	-0.75 (0.41)	33◊
	MTB	31 V1	2.20 (0.42)	1.58 (0.40)	-0.62 (0.35)	28◊
	CO-PTB	32 V4	1.84 (0.32)	1.47 (0.37)	-0.37 (0.28)	20◊
	MTB	31 V4	1.86 (0.46)	1.45 (0.37)	-0.40 (0.32)	22◊
Van der Weijden et al.(1996)	OR-PTB	20	2.1 (0.3)	0.8 (0.3)	-1.3◊	62
	CO-PTB	20	2.2 (0.3)	0.8 (0.3)	-1.4◊	64
	Cir-PTB	20	2.2 (0.3)	0.9 (0.3)	-1.3◊	61

	MTB	20	2.2 (0.3)	0.90.9 (0.3)	-1.3◊	57
Heasman et al.(1999)	OR-PTB	25	2.03 (0.55)	0.82 (0.54)	-1.21◊	60◊
	OR-PTB	25	2.17 (0.58)	0.92 (0.42)	-1.25◊	58◊
	MTB	24	2.27 (0.54)	1.05 (0.51)	-1.22 ◊	54◊
Renton-Harper et al.(2001)	OR-PTB	16	3.137 (0.147)	2.481 (0.349)	-0.656◊	21◊
	OR-PTB	16	3.205 (0.240)	2.464 (0.368)	-0.741◊	23◊
	MTB	16	3.179 (0.208)	2.402 (0.414)	-0.777◊	24◊
	OR-PTB	16	3.137 (0.147)	2.095 (0.433)	-1.042◊	33◊
	OR-PTB	16	3.205 (0.240)	2.132 (0.467)	-1.073◊	33◊
	MTB	16	3.179 (0.208)	2.020 (0.417)	-1.159◊	36◊
	OR-PTB	16	3.137 (0.147)	1.736 (0.474)	-1.401◊	45◊
	OR-PTB	16	3.205 (0.240)	1.775 (0.478)	-1.430◊	45◊
	MTB	16	3.179 (0.208)	1.669 (0.358)	-1.51◊	47◊

Moritis et al.(2002)	SS-PTB	25	2.70 (0.40)	1.73 (0.47)	-0.97◊	36◊
	MTB	25	2.55 (0.44)	1.89 (0.47)	-0.66 ◊	26◊
Pizzo et al.(2010)	OR-PTB	66	3.40 (0.67)	2.43 (0.78)	-0.97 (0.43)	29◊
	MTB	66	3.42 (0.69)	2.65 (0.86)	-0.78 (0.38)	23◊
	MTB	66	3.36 (0.8)	2.86 (0.9)	-0.49 (0.33)	15◊
Klukowska et al.(2012)	OR-PTB with SS movement	36	2.146 (0.3551) ♦	1.104 (0.4017) ♦	-1.042 (0.2174) ♦	49
	MTB	36	2.169 (0.3230) ♦	1.196 (0.4080) ♦	-0.973 (0.2516) ♦	45
Rosema et al.(2014)	OR-PTB	91	2.80 (0.450)	1.56 (0.471)	-1.24 (0.301)	44◊
	MTB	90	2.50 (0.366)	1.51 (0.349)	-1.05 (0.268)	40◊
Re et al.(2015)	SS-PTB	40	1.85 (0.51)	0.66 (0.47)	-1.19 (0.37)	64◊
	MTB	40	1.83 (0.65)	0.78 (0.48)	-1.05 (0.22)	57◊
Kurtz	OR-PTB	90	2.331 (0.4842) ♦	1.604 (0.4353) ♦	-0.726 (0.2996) ♦	31

et al.(2016)	MTB	90	2.307 (0.5212) ♦	1.741 (0.4293) ♦	-0.566 (0.2429) ♦	25
	OR-PTB	90	2.275 (0.4306) ♦	1.527 (0.3770) ♦	-0.748 (0.2568) ♦	33
	MTB	90	2.272 (0.4501) ♦	1.677 (0.3891) ♦	-0.594 (0.2158) ♦	26
	OR-PTB	60	2.325 (0.3954) ♦	1.508 (0.4205) ♦	-0.817 (0.2705) ♦	35
	MTB	60	2.310 (0.4378) ♦	1.597 (0.3662) ♦	-0.713 (0.2490) ♦	31
	OR-PTB	Ex2b 32	2.203 (0.5313) ♦	1.555 (0.3931) ♦	-0.649(0.2470) ♦	29
	MTB	Ex2b 32	2.167 (0.4892) ♦	1.695 (0.3953) ♦	-0.473 (0.2117) ♦	22
Kulkarni et al.(2017)	OR-PTB	22 V1	1.77 (0.34)	0.81 (0.39)	-0.9568 (0.4233) ◊	54◊
	MTB	23 V1	1.56 (0.44)	0.61 (0.34)	-0.9461 (0.4066) ◊	61◊
	OR-PTB	22 V2	1.43 (0.56)	0.54 (0.36)	-0.8845 (0.3938) ◊	62◊
	MTB	23 V2	1.53 (0.54)	0.58 (0.42)	-0.9570 (0.4088) ◊	62◊
	OR-PTB	22 V3	0.98 (0.45)	0.25 (0.19)	-0.7246 (0.3701) ◊	74◊
	MTB	23 V3	0.88 (0.37)	0.29 (0.24)	-0.5922 (0.3236) ◊	67◊
	OR-PTB	22 V4	0.70 (0.44)	0.13 (0.13)	-0.5664 (0.4223) ◊	81◊

	MTB	23<br>V4	0.71 (0.31)	0.17 (0.19)	-0.5370 (0.2619) ◊	76◊

◊ Calculated by the authors when not provided in the manuscript.

♦ data provided by the author

□ = no data available

OR = Oscillating Rotating SS = Side to Side CO= Counter rotating Cir = Ciculair

Calculations for PRE, POST, DIFF means based on the formula: POST= PRE-DIFF.

For standard deviations calculations were based on the formula: (SD=SE*√N).

Appendix S4

Studies using the RMNPI

Paper		N	Brushing mean (SD)			
			PRE	POST	DIFF	
					Absolute	%
Sharma et al.(2006)	SS-PTB	35	0.66 (0.04)	0.16 (0.09)	-0.50 (0.08)	76◊
	SS-PTB	36	0.66 (0.04)	0.11 (0.06)	-0.54 (0.05)	82◊
	SS-PTB	36	0.66 (0.04)	0.07 (0.05)	-0.59 (0.05)	89◊
	MTB	37	0.65 (0.03)	0.14 (0.07)	-0.51 (0.06)	78◊
Sharma et al.(2011)	OR-PTB	40	0.632 (0.0223)	0.076 (0.0260)	-0.556 (0.0268)	88
	MTB	40	0.625 (0.0264)	0.195 (0.0557)	-0.431 (0.0427)	69
Nathoo et al.(2012)	SS-PTB	40	0.75 (0.09)	0.35 (0.08)	-0.40 (0.08)	53◊
	MTB	36	0.74 (0.09)	0.49 (0.08)	-0.25 (0.05)	34◊
Nathoo et al.(2014)	SS-PTB	40	0.71 (0.10)	0.30 (0.10)	-0.41 (0.07)	58

	SS-PTB	40	0.75 (0.11)	0.32 (0.10)	-0.43 (0.09)	57
	MTB	40	0.72 (0.14)	0.45 (0.13)	-0.27 (0.15)	38
Gallob et al.(2015)	SS-PTB	39	0.66 (0.04)	0.27 (0.07)	-0.39 (0.06)	59
	MTB	40	0.65 (0.04)	0.41 (0.09)	-0.24 (0.07)	37

◊ Calculated by the authors when not provided in the manuscript.
♦ data provided by the author
OR = Oscillating Rotating SS = Side to Side
Calculations for PRE, POST, DIFF means based on the formula: POST= PRE-DIFF.
For standard deviations calculations were based on the formula: (SD=SE*√N).

Appendix S5

A descriptive summary of statistical significance of PTB compared to MTB

	Study #	Intervention by mode of action	Plaque score	Comparison
**Q&H**	Van der Weijden et al. (1996)	OR-PTB	**+**	MTB
	Heasman et al. (1999)	OR-PTB	**0**	MTB
		OR-PTB	**0**	MTB
	Renton-Harper et al. (2001)	OR-PTB	**0**	MTB
		OR-PTB	**0**	MTB
		OR-PTB	**0**	MTB
		OR-PTB	**0**	MTB
		OR-PTB	**0**	MTB
		OR-PTB	**0**	MTB
	Pizzo et al. (2010)	OR-PTB	**+**	MTB
		OR-PTB	**+**	MTB

	Rosema et al. (2014)	OR-PTB	+	MTB
	Kurtz et al. (2016)	OR-PTB	+	MTB
		OR-PTB	+	MTB
		OR-PTB	+	MTB
		OR-PTB	+	MTB
	Kulkarni et al.(2018)	OR-PTB	?	MTB
		OR-PTB	?	MTB
		OR-PTB	?	MTB
		OR-PTB	?	MTB
**RMN**	Sharma et al. (2011)	OR-PTB	+	MTB
**Q&H**	Moritis et al. (2002)	SS-PTB	+	MTB

	Re et al. (2015)	SS-PTB	+	MTB
**RMN**	Sharma et al.(2006)	SS-PTB	+	MTB
		SS-PTB	+	MTB
		SS-PTB	+	MTB
	Nathoo et al. (2012)	SS-PTB	+	MTB
	Nathoo et al. (2014)	SS-PTB	+	MTB
		SS-PTB	+	MTB
	Gallob et al. (2015)	SS-PTB	+	MTB
**other**	Killoy et al. (1989)	CO-PTB	+	MTB
	Khocht et al. (1992)	CO-PTB	?	MTB
		CO-PTB	?	MTB
	Van der Weijden et al. (1996)	CO-PTB	+	MTB
	Klukowska et al. (2012)	OR-PTB with SS movement	+	MTB

	.<br>Van der Weijden et al. (1996)	Cir-PTB	**+**	MTB

+ = significant difference in favor of the PTB - = significant difference in favor of the MTB
0 = no significant difference ? = unknown
OR = Oscillating Rotating SS = Side to Side CO= Counter rotating Cir = Ciculair

Appendix S6a

Forest plot of the overall analysis for PTB compared to MTB at pre-brushing in plaque scores on the Q&HPI

	ptb			mtb				Mean Difference
Study or Subgroup	Mean	SD	Total	Mean	SD	Total	Weight	IV, Random, 95% CI
**23.1.1 OR**								
Heasman et al. 1999 I	2.03	0.55	25	2.27	0.54	12	1.2%	-0.24 [-0.61, 0.13]
Heasman et al. 1999 II	2.17	0.58	25	2.27	0.54	12	1.2%	-0.10 [-0.48, 0.28]
Kulkarni et al. 2018 I	1.77	0.34	22	1.56	0.4	23	3.3%	0.21 [-0.01, 0.43]
Kulkarni et al. 2018 II	1.43	0.56	22	1.53	0.54	23	1.6%	-0.10 [-0.42, 0.22]
Kulkarni et al. 2018 III	0.98	0.45	22	0.88	0.37	23	2.7%	0.10 [-0.14, 0.34]
Kulkarni et al. 2018 IV	0.7	0.44	22	0.71	0.31	23	3.1%	-0.01 [-0.23, 0.21]
Kurtz et al. 2016 I	2.331	0.4842	90	2.307	0.5212	90	6.4%	0.02 [-0.12, 0.17]
Kurtz et al. 2016 II	2.275	0.4306	90	2.272	0.4501	90	7.9%	0.00 [-0.13, 0.13]
Kurtz et al. 2016 III	2.325	0.3954	60	2.31	0.4378	60	6.3%	0.02 [-0.13, 0.16]
Kurtz et al. 2016 IV	2.203	0.5313	32	2.167	0.4892	32	2.5%	0.04 [-0.21, 0.29]
Pizzo et al. 2009 I	3.42	0.67	33	3.42	0.69	66	2.0%	0.00 [-0.28, 0.28]
Pizzo et al. 2009 II	3.42	0.67	33	3.36	0.8	66	1.8%	0.06 [-0.24, 0.36]
Renton-Harper et al. 2001 I	3.137	0.147	16	3.179	0.208	8	5.5%	-0.04 [-0.20, 0.12]
Renton-Harper et al. 2001 II	3.205	0.24	16	3.179	0.208	8	4.3%	0.03 [-0.16, 0.21]
Renton-Harper et al. 2001 III	3.137	0.147	16	3.179	0.208	8	5.5%	-0.04 [-0.20, 0.12]
Renton-Harper et al. 2001 IV	3.205	0.24	16	3.179	0.208	8	4.3%	0.03 [-0.16, 0.21]
Renton-Harper et al. 2001 V	3.237	0.147	16	3.179	0.208	8	5.5%	0.06 [-0.10, 0.22]
Renton-Harper et al. 2001 VI	3.205	0.24	16	3.179	0.208	8	4.3%	0.03 [-0.16, 0.21]
Rosema et al. 2014 I	2.8	0.45	91	2.5	0.366	90	8.8%	0.30 [0.18, 0.42]
Van der Weijden et al. 1996 I	2.1	0.3	20	2.2	0.3	7	2.4%	-0.10 [-0.36, 0.16]
**Subtotal (95% CI)**			**683**			**665**	**80.9%**	**0.04 [-0.02, 0.09]**
Heterogeneity: Tau² = 0.00; Chi² = 28.08, df = 19 (P = 0.08); I² = 32%								
Test for overall effect: Z = 1.34 (P = 0.18)								
**23.1.2 SS**								
Moritis et al. 2002 I	2.7	0.4	25	2.55	2.8	25	0.1%	0.15 [-0.96, 1.26]
Re et al. 2015 I	1.85	0.65	40	1.83	2.1	40	0.4%	0.02 [-0.66, 0.70]
**Subtotal (95% CI)**			**65**			**65**	**0.5%**	**0.06 [-0.52, 0.64]**
Heterogeneity: Tau² = 0.00; Chi² = 0.04, df = 1 (P = 0.84); I² = 0%								
Test for overall effect: Z = 0.19 (P = 0.85)								
**23.1.3 other**								
Khocht et al. 1992 I	2.27	0.36	32	2.2	0.42	31	4.0%	0.07 [-0.12, 0.26]
Khocht et al. 1992 II	1.84	0.32	32	1.86	0.46	31	3.9%	-0.02 [-0.22, 0.18]
Klukowska et al. 2012 I	2.146	0.3551	36	2.169	0.323	36	5.8%	-0.02 [-0.18, 0.13]
Van der Weijden et al. 1996 II	2.2	0.3	20	2.2	0.3	7	2.4%	0.00 [-0.26, 0.26]
Van der Weijden et al. 1996 III	2.2	0.3	20	2.2	0.3	7	2.4%	0.00 [-0.26, 0.26]
**Subtotal (95% CI)**			**140**			**112**	**18.6%**	**0.00 [-0.09, 0.09]**
Heterogeneity: Tau² = 0.00; Chi² = 0.62, df = 4 (P = 0.96); I² = 0%								
Test for overall effect: Z = 0.07 (P = 0.94)								
**Total (95% CI)**			**888**			**842**	**100.0%**	**0.04 [-0.01, 0.08]**
Heterogeneity: Tau² = 0.00; Chi² = 29.50, df = 26 (P = 0.29); I² = 12%								
Test for overall effect: Z = 1.67 (P = 0.09)								
Test for subgroup differences: Chi² = 0.40, df = 2 (P = 0.82), I² = 0%								

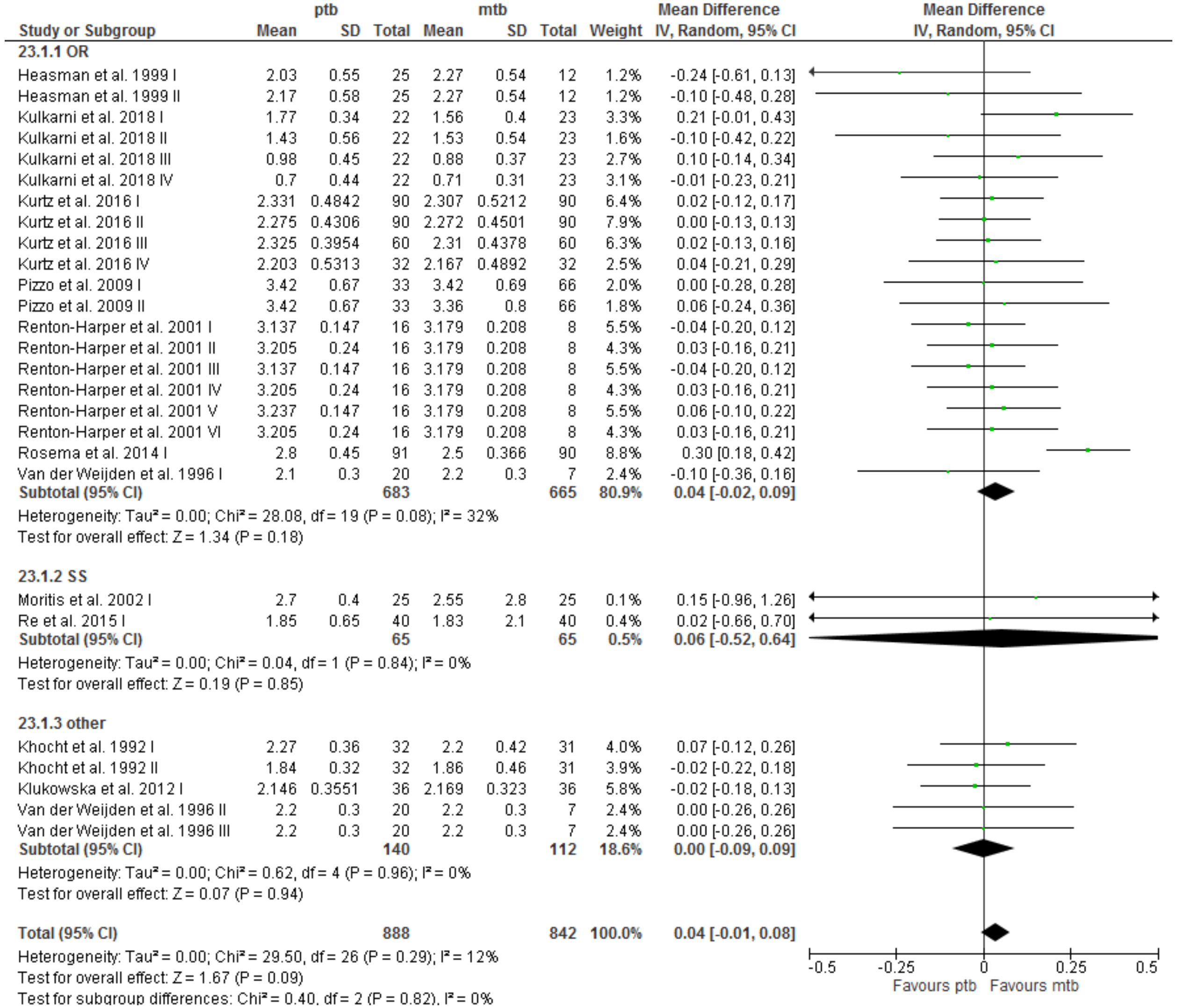

Appendix S6b

Forest plot of the random subanalysis for the OR mode of action for PTB compared to MTB at pre-brushing in plaque scores on the Q&HPI

Study or Subgroup	ptb Mean	ptb SD	ptb Total	mtb Mean	mtb SD	mtb Total	Weight	Mean Difference IV, Random, 95% CI
**23.1.1 OR**								
Heasman et al. 1999 I	2.03	0.55	25	2.27	0.54	12	1.8%	-0.24 [-0.61, 0.13]
Heasman et al. 1999 II	2.17	0.58	25	2.27	0.54	12	1.8%	-0.10 [-0.48, 0.28]
Kulkarni et al. 2018 I	1.77	0.34	22	1.56	0.4	23	4.5%	0.21 [-0.01, 0.43]
Kulkarni et al. 2018 II	1.43	0.56	22	1.53	0.54	23	2.4%	-0.10 [-0.42, 0.22]
Kulkarni et al. 2018 III	0.98	0.45	22	0.88	0.37	23	3.8%	0.10 [-0.14, 0.34]
Kulkarni et al. 2018 IV	0.7	0.44	22	0.71	0.31	23	4.3%	-0.01 [-0.23, 0.21]
Kurtz et al. 2016 I	2.331	0.4842	90	2.307	0.5212	90	7.4%	0.02 [-0.12, 0.17]
Kurtz et al. 2016 II	2.275	0.4306	90	2.272	0.4501	90	8.5%	0.00 [-0.13, 0.13]
Kurtz et al. 2016 III	2.325	0.3954	60	2.31	0.4378	60	7.3%	0.02 [-0.13, 0.16]
Kurtz et al. 2016 IV	2.203	0.5313	32	2.167	0.4892	32	3.6%	0.04 [-0.21, 0.29]
Pizzo et al. 2009 I	3.42	0.67	33	3.42	0.69	66	3.0%	0.00 [-0.28, 0.28]
Pizzo et al. 2009 II	3.42	0.67	33	3.36	0.8	66	2.7%	0.06 [-0.24, 0.36]
Renton-Harper et al. 2001 I	3.137	0.147	16	3.179	0.208	8	6.6%	-0.04 [-0.20, 0.12]
Renton-Harper et al. 2001 II	3.205	0.24	16	3.179	0.208	8	5.5%	0.03 [-0.16, 0.21]
Renton-Harper et al. 2001 III	3.137	0.147	16	3.179	0.208	8	6.6%	-0.04 [-0.20, 0.12]
Renton-Harper et al. 2001 IV	3.205	0.24	16	3.179	0.208	8	5.5%	0.03 [-0.16, 0.21]
Renton-Harper et al. 2001 V	3.237	0.147	16	3.179	0.208	8	6.6%	0.06 [-0.10, 0.22]
Renton-Harper et al. 2001 VI	3.205	0.24	16	3.179	0.208	8	5.5%	0.03 [-0.16, 0.21]
Rosema et al. 2014 I	2.8	0.45	91	2.5	0.366	90	9.1%	0.30 [0.18, 0.42]
Van der Weijden et al. 1996 I	2.1	0.3	20	2.2	0.3	7	3.4%	-0.10 [-0.36, 0.16]
**Subtotal (95% CI)**			**683**			**665**	**100.0%**	**0.04 [-0.02, 0.09]**
Heterogeneity: Tau² = 0.00; Chi² = 28.08, df = 19 (P = 0.08); I² = 32%								
Test for overall effect: Z = 1.34 (P = 0.18)								
**23.1.2 SS**								
Moritis et al. 2002 I	2.7	0.4	25	2.55	2.8	25	0.0%	0.15 [-0.96, 1.26]
Re et al. 2015 I	1.85	0.65	40	1.83	2.1	40	0.0%	0.02 [-0.66, 0.70]
**Subtotal (95% CI)**			**0**			**0**		**Not estimable**
Heterogeneity: Not applicable								
Test for overall effect: Not applicable								
**23.1.3 other**								
Khocht et al. 1992 I	2.27	0.36	32	2.2	0.42	31	0.0%	0.07 [-0.12, 0.26]
Khocht et al. 1992 II	1.84	0.32	32	1.86	0.46	31	0.0%	-0.02 [-0.22, 0.18]
Klukowska et al. 2012 I	2.146	0.3551	36	2.169	0.323	36	0.0%	-0.02 [-0.18, 0.13]
Van der Weijden et al. 1996 II	2.2	0.3	20	2.2	0.3	7	0.0%	0.00 [-0.26, 0.26]
Van der Weijden et al. 1996 III	2.2	0.3	20	2.2	0.3	7	0.0%	0.00 [-0.26, 0.26]
**Subtotal (95% CI)**			**0**			**0**		**Not estimable**
Heterogeneity: Not applicable								
Test for overall effect: Not applicable								
**Total (95% CI)**			**683**			**665**	**100.0%**	**0.04 [-0.02, 0.09]**
Heterogeneity: Tau² = 0.00; Chi² = 28.08, df = 19 (P = 0.08); I² = 32%								
Test for overall effect: Z = 1.34 (P = 0.18)								
Test for subgroup differences: Not applicable								

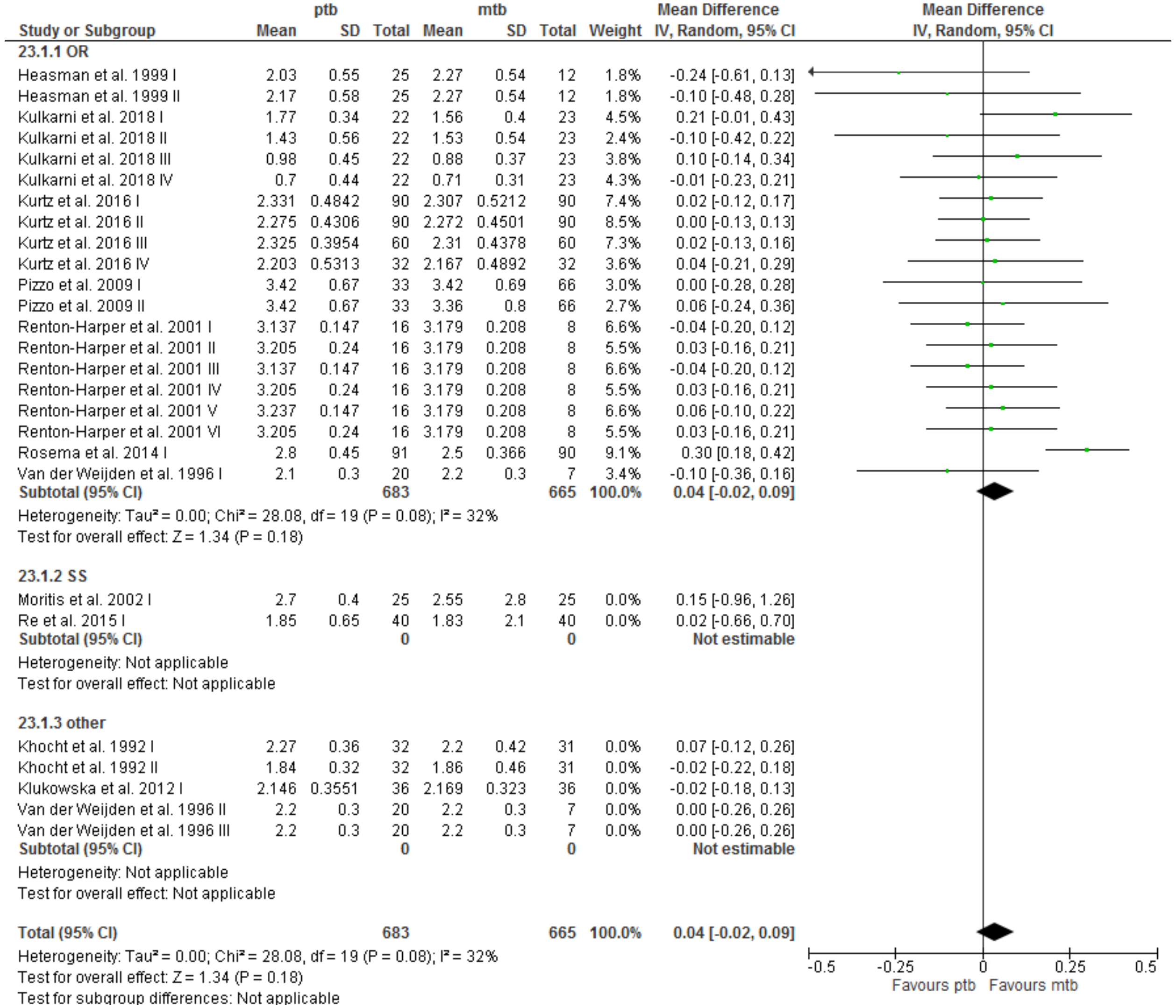

Appendix S6c

Forest plot of the fixed subanalysis for the SS mode of action for PTB compared to MTB at pre-brushing in plaque scores on the Q&HPI

	ptb			mtb				Mean Difference
**Study or Subgroup**	**Mean**	**SD**	**Total**	**Mean**	**SD**	**Total**	**Weight**	**IV, Fixed, 95% CI**
**23.1.1 OR**								
Heasman et al. 1999 I	2.03	0.55	25	2.27	0.54	12	0.0%	-0.24 [-0.61, 0.13]
Heasman et al. 1999 II	2.17	0.58	25	2.27	0.54	12	0.0%	-0.10 [-0.48, 0.28]
Kulkarni et al. 2018 I	1.77	0.34	22	1.56	0.4	23	0.0%	0.21 [-0.01, 0.43]
Kulkarni et al. 2018 II	1.43	0.56	22	1.53	0.54	23	0.0%	-0.10 [-0.42, 0.22]
Kulkarni et al. 2018 III	0.98	0.45	22	0.88	0.37	23	0.0%	0.10 [-0.14, 0.34]
Kulkarni et al. 2018 IV	0.7	0.44	22	0.71	0.31	23	0.0%	-0.01 [-0.23, 0.21]
Kurtz et al. 2016 I	2.331	0.4842	90	2.307	0.5212	90	0.0%	0.02 [-0.12, 0.17]
Kurtz et al. 2016 II	2.275	0.4306	90	2.272	0.4501	90	0.0%	0.00 [-0.13, 0.13]
Kurtz et al. 2016 III	2.325	0.3954	60	2.31	0.4378	60	0.0%	0.02 [-0.13, 0.16]
Kurtz et al. 2016 IV	2.203	0.5313	32	2.167	0.4892	32	0.0%	0.04 [-0.21, 0.29]
Pizzo et al. 2009 I	3.42	0.67	33	3.42	0.69	66	0.0%	0.00 [-0.28, 0.28]
Pizzo et al. 2009 II	3.42	0.67	33	3.36	0.8	66	0.0%	0.06 [-0.24, 0.36]
Renton-Harper et al. 2001 I	3.137	0.147	16	3.179	0.208	8	0.0%	-0.04 [-0.20, 0.12]
Renton-Harper et al. 2001 II	3.205	0.24	16	3.179	0.208	8	0.0%	0.03 [-0.16, 0.21]
Renton-Harper et al. 2001 III	3.137	0.147	16	3.179	0.208	8	0.0%	-0.04 [-0.20, 0.12]
Renton-Harper et al. 2001 IV	3.205	0.24	16	3.179	0.208	8	0.0%	0.03 [-0.16, 0.21]
Renton-Harper et al. 2001 V	3.237	0.147	16	3.179	0.208	8	0.0%	0.06 [-0.10, 0.22]
Renton-Harper et al. 2001 VI	3.205	0.24	16	3.179	0.208	8	0.0%	0.03 [-0.16, 0.21]
Rosema et al. 2014 I	2.8	0.45	91	2.5	0.366	90	0.0%	0.30 [0.18, 0.42]
Van der Weijden et al. 1996 I	2.1	0.3	20	2.2	0.3	7	0.0%	-0.10 [-0.36, 0.16]
**Subtotal (95% CI)**			**0**			**0**		**Not estimable**
Heterogeneity: Not applicable								
Test for overall effect: Not applicable								
**23.1.2 SS**								
Moritis et al. 2002 I	2.7	0.4	25	2.55	2.8	25	27.4%	0.15 [-0.96, 1.26]
Re et al. 2015 I	1.85	0.65	40	1.83	2.1	40	72.6%	0.02 [-0.66, 0.70]
**Subtotal (95% CI)**			**65**			**65**	**100.0%**	**0.06 [-0.52, 0.64]**
Heterogeneity: Chi² = 0.04, df = 1 (P = 0.84); I² = 0%								
Test for overall effect: Z = 0.19 (P = 0.85)								
**23.1.3 other**								
Khocht et al. 1992 I	2.27	0.36	32	2.2	0.42	31	0.0%	0.07 [-0.12, 0.26]
Khocht et al. 1992 II	1.84	0.32	32	1.86	0.46	31	0.0%	-0.02 [-0.22, 0.18]
Klukowska et al. 2012 I	2.146	0.3551	36	2.169	0.323	36	0.0%	-0.02 [-0.18, 0.13]
Van der Weijden et al. 1996 II	2.2	0.3	20	2.2	0.3	7	0.0%	0.00 [-0.26, 0.26]
Van der Weijden et al. 1996 III	2.2	0.3	20	2.2	0.3	7	0.0%	0.00 [-0.26, 0.26]
**Subtotal (95% CI)**			**0**			**0**		**Not estimable**
Heterogeneity: Not applicable								
Test for overall effect: Not applicable								
**Total (95% CI)**			**65**			**65**	**100.0%**	**0.06 [-0.52, 0.64]**
Heterogeneity: Chi² = 0.04, df = 1 (P = 0.84); I² = 0%								
Test for overall effect: Z = 0.19 (P = 0.85)								
Test for subgroup differences: Not applicable								

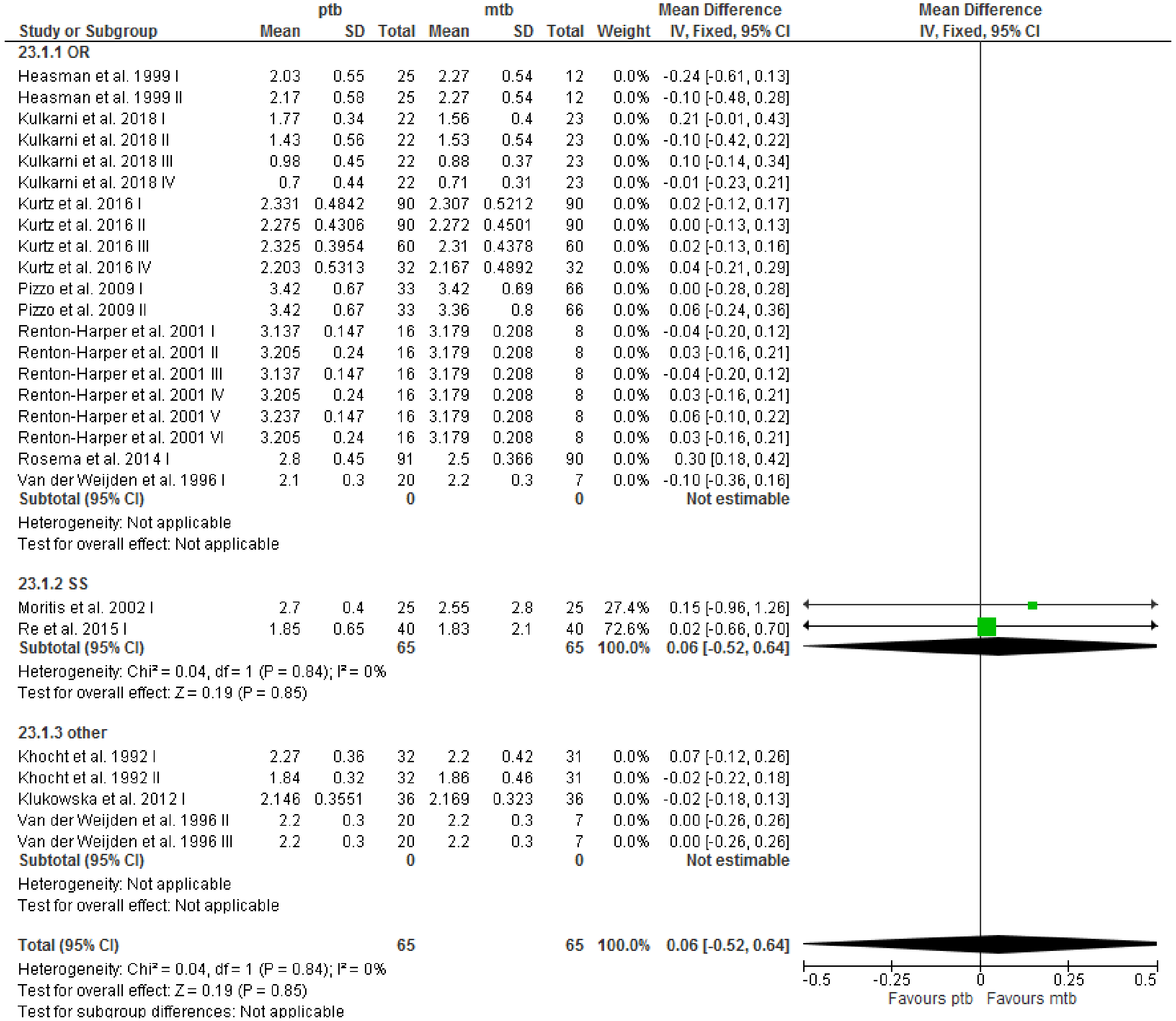

Appendix S6d

Forest plot of the random subanalysis for the OR mode of action by product (P&G) for PTB compared to MTB at pre-brushing in plaque scores on the Q&HPI

	ptb			mtb				Mean Difference
Study or Subgroup	Mean	SD	Total	Mean	SD	Total	Weight	IV, Random, 95% CI
Heasman et al. 1999 II	2.17	0.58	25	2.27	0.54	12	1.4%	-0.10 [-0.48, 0.28]
Kulkarni et al. 2018 I	1.77	0.34	22	1.56	0.4	23	4.4%	0.21 [-0.01, 0.43]
Kulkarni et al. 2018 II	1.43	0.56	22	1.53	0.54	23	2.0%	-0.10 [-0.42, 0.22]
Kulkarni et al. 2018 III	0.98	0.45	22	0.88	0.37	23	3.6%	0.10 [-0.14, 0.34]
Kulkarni et al. 2018 IV	0.7	0.44	22	0.71	0.31	23	4.1%	-0.01 [-0.23, 0.21]
Kurtz et al. 2016 I	2.331	0.4842	90	2.307	0.5212	90	9.6%	0.02 [-0.12, 0.17]
Kurtz et al. 2016 II	2.275	0.4306	90	2.272	0.4501	90	12.5%	0.00 [-0.13, 0.13]
Kurtz et al. 2016 III	2.325	0.3954	60	2.31	0.4378	60	9.3%	0.02 [-0.13, 0.16]
Kurtz et al. 2016 IV	2.203	0.5313	32	2.167	0.4892	32	3.3%	0.04 [-0.21, 0.29]
Pizzo et al. 2009 I	3.42	0.67	33	3.42	0.69	66	2.6%	0.00 [-0.28, 0.28]
Pizzo et al. 2009 II	3.42	0.67	33	3.36	0.8	66	2.3%	0.06 [-0.24, 0.36]
Renton-Harper et al. 2001 I	3.137	0.147	16	3.179	0.208	8	8.0%	-0.04 [-0.20, 0.12]
Renton-Harper et al. 2001 II	3.205	0.24	16	3.179	0.208	8	6.0%	0.03 [-0.16, 0.21]
Renton-Harper et al. 2001 III	3.137	0.147	16	3.179	0.208	8	8.0%	-0.04 [-0.20, 0.12]
Renton-Harper et al. 2001 IV	3.205	0.24	16	3.179	0.208	8	6.0%	0.03 [-0.16, 0.21]
Renton-Harper et al. 2001 V	3.237	0.147	16	3.179	0.208	8	8.0%	0.06 [-0.10, 0.22]
Renton-Harper et al. 2001 VI	3.205	0.24	16	3.179	0.208	8	6.0%	0.03 [-0.16, 0.21]
Van der Weijden et al. 1996 I	2.1	0.3	20	2.2	0.3	7	3.1%	-0.10 [-0.36, 0.16]
**Total (95% CI)**			**567**			**563**	**100.0%**	**0.02 [-0.03, 0.06]**

Heterogeneity: Tau² = 0.00; Chi² = 6.69, df = 17 (P = 0.99); I² = 0%

Test for overall effect: Z = 0.65 (P = 0.52)

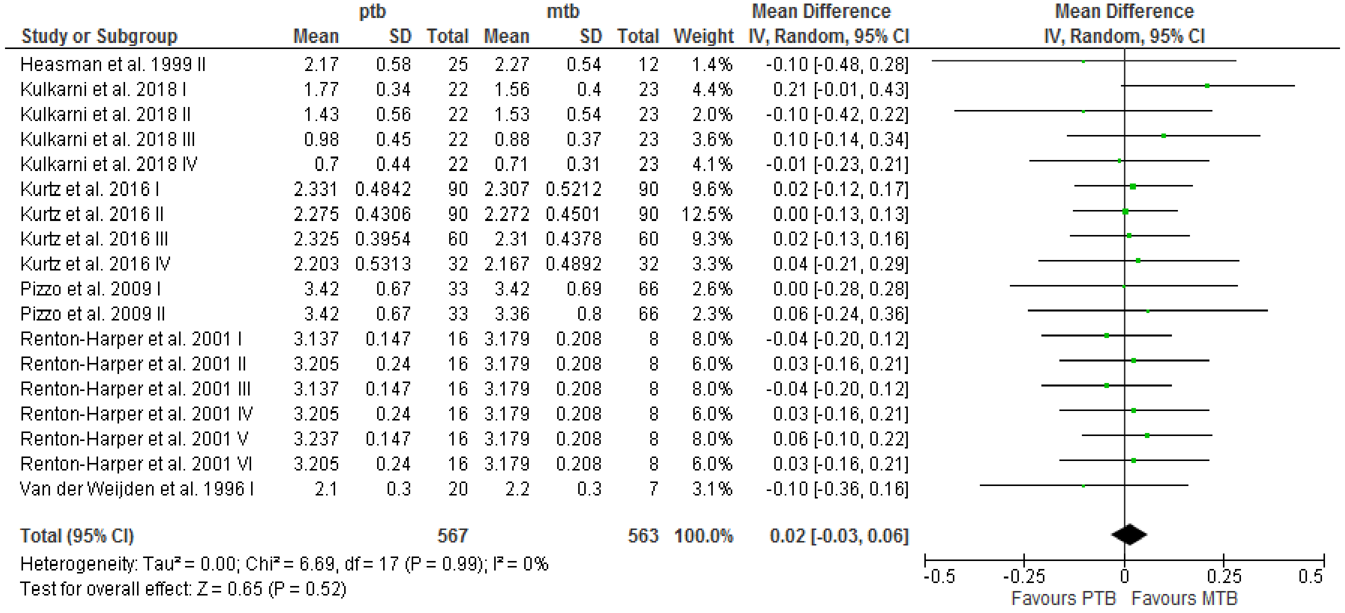

Appendix S7a

Funnel plot for the overall analysis for PTB compared to MTB at pre-brushing in plaque scores on the Q&HPI

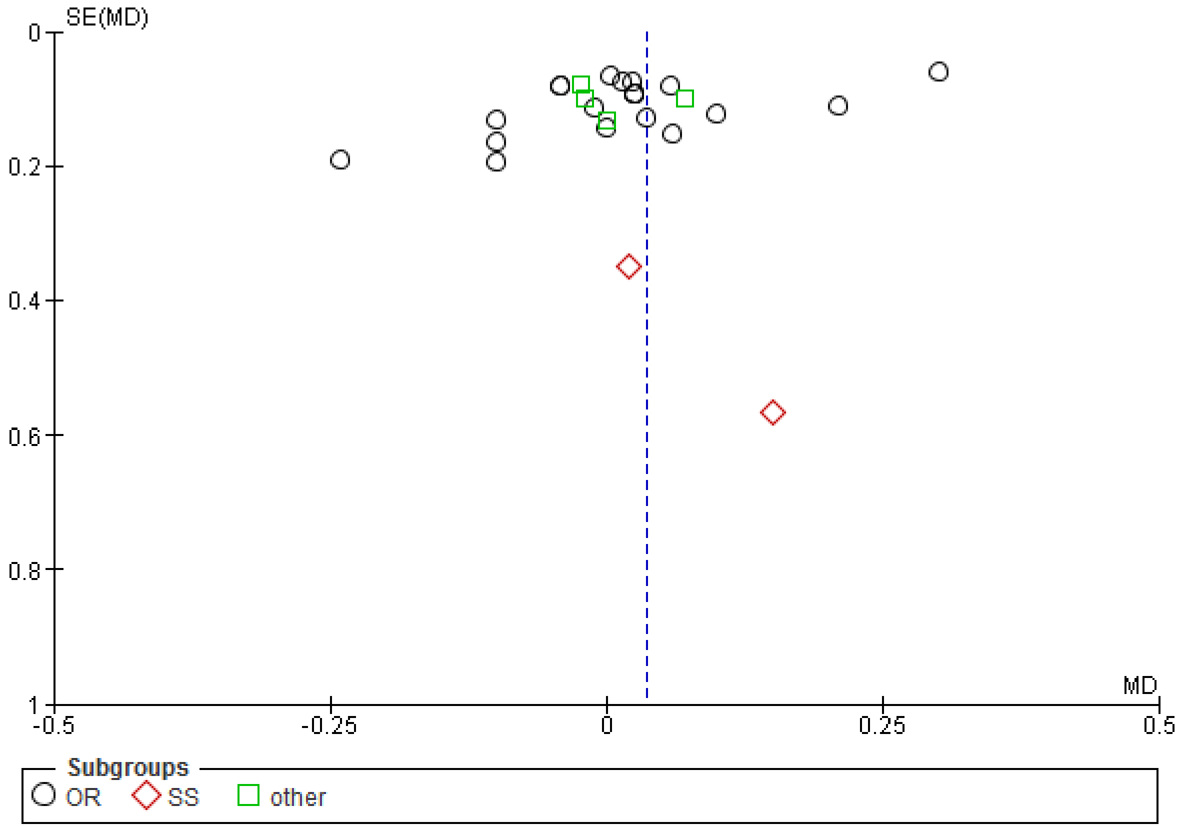

Appendix S7b

Funnel plot for the random subanalysis for the OR mode of action for PTB compared to MTB at pre-brushing in plaque scores on the Q&HPI

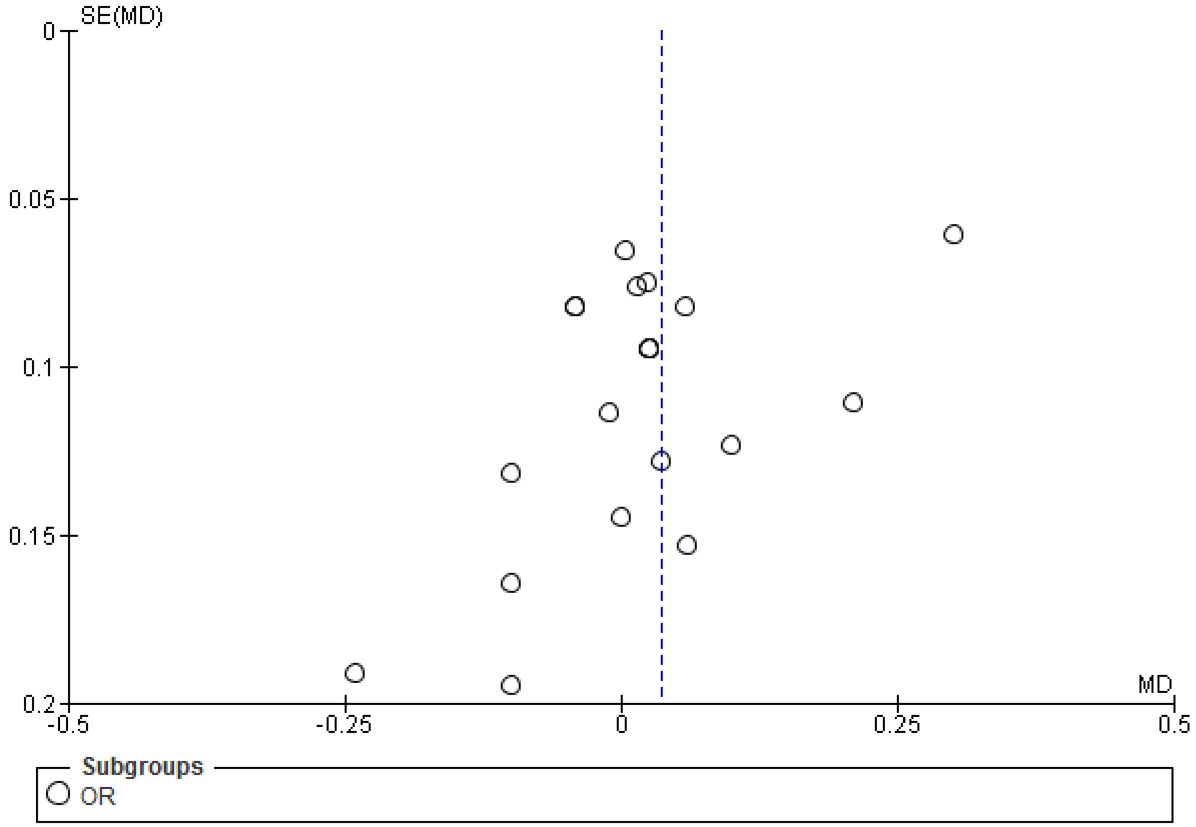

Appendix S7c

Funnel plot for the random subanalysis for the OR mode of action by product (P&G) for PTB compared to MTB at pre-brushing in plaque scores on the Q&HPI

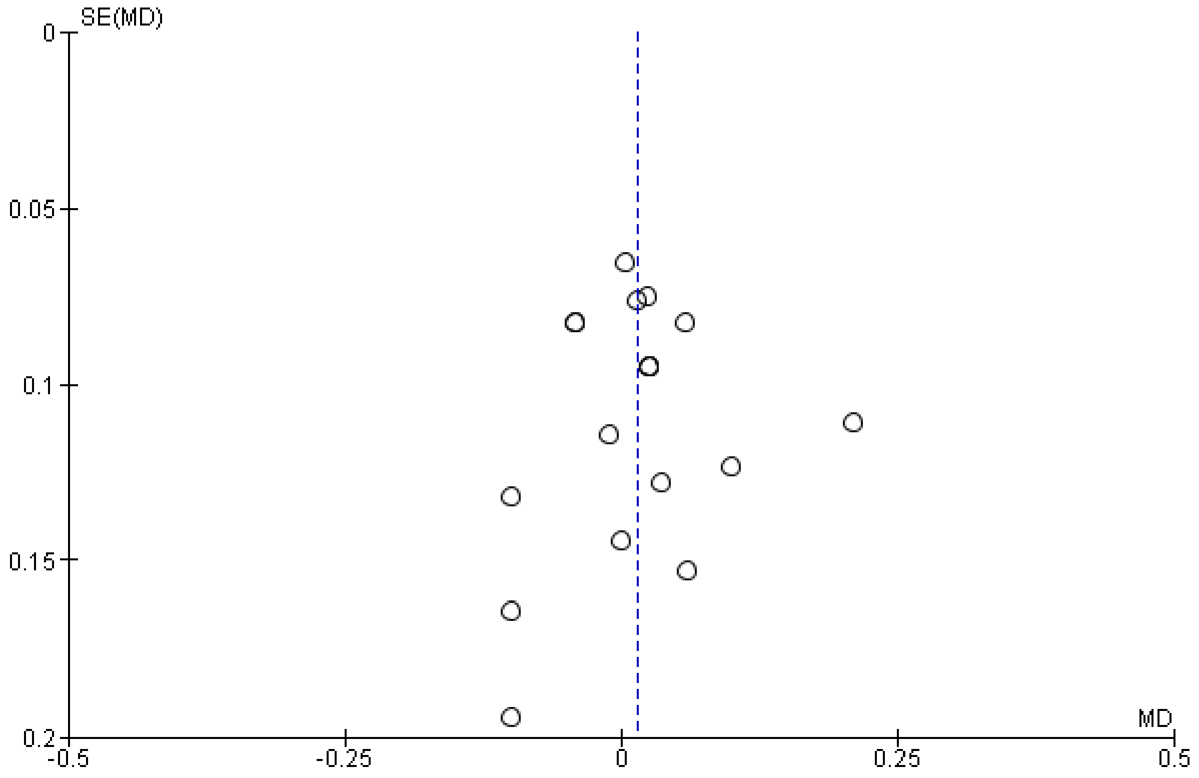

Appendix S8a

Forest plot of the overall analysis for PTB compared to MTB at post-brushing in plaque scores on the Q&HPI

Study or Subgroup	ptb Mean	ptb SD	ptb Total	mtb Mean	mtb SD	mtb Total	Weight	Mean Difference IV, Random, 95% CI
**23.2.1 OR**								
Heasman et al. 1999 I	0.82	0.54	25	1.05	0.51	12	1.1%	-0.23 [-0.59, 0.13]
Heasman et al. 1999 II	0.92	0.42	25	1.05	0.51	12	1.3%	-0.13 [-0.46, 0.20]
Kulkarni et al. 2018 I	0.81	0.39	22	0.61	0.34	23	3.1%	0.20 [-0.01, 0.41]
Kulkarni et al. 2018 II	0.54	0.36	22	0.58	0.42	23	2.8%	-0.04 [-0.27, 0.19]
Kulkarni et al. 2018 III	0.25	0.19	22	0.29	1.24	23	0.5%	-0.04 [-0.55, 0.47]
Kulkarni et al. 2018 IV	0.13	0.13	22	0.17	0.19	23	15.4%	-0.04 [-0.13, 0.05]
Kurtz et al. 2016 I	1.604	0.4353	90	1.741	0.4293	90	8.8%	-0.14 [-0.26, -0.01]
Kurtz et al. 2016 II	1.527	0.377	90	1.677	0.3891	90	11.2%	-0.15 [-0.26, -0.04]
Kurtz et al. 2016 III	1.508	0.4205	60	1.597	0.3662	90	8.3%	-0.09 [-0.22, 0.04]
Kurtz et al. 2016 IV	1.555	0.3931	32	1.695	0.3953	32	3.8%	-0.14 [-0.33, 0.05]
Pizzo et al. 2009 I	2.43	0.78	33	2.65	0.86	66	1.3%	-0.22 [-0.56, 0.12]
Pizzo et al. 2009 II	2.43	0.78	33	2.86	0.9	66	1.2%	-0.43 [-0.77, -0.09]
Renton-Harper et al. 2001 I	2.481	0.349	16	2.402	0.414	8	1.3%	0.08 [-0.25, 0.41]
Renton-Harper et al. 2001 II	2.464	0.368	16	2.402	0.414	8	1.3%	0.06 [-0.28, 0.40]
Renton-Harper et al. 2001 III	2.095	0.433	16	2.02	0.417	8	1.1%	0.08 [-0.28, 0.43]
Renton-Harper et al. 2001 IV	2.132	0.467	16	2.02	0.417	8	1.1%	0.11 [-0.26, 0.48]
Renton-Harper et al. 2001 V	1.736	0.474	16	1.669	0.358	8	1.2%	0.07 [-0.27, 0.41]
Renton-Harper et al. 2001 VI	1.775	0.478	16	1.669	0.358	8	1.2%	0.11 [-0.24, 0.45]
Rosema et al. 2014 I	1.56	0.471	91	1.51	0.349	90	9.7%	0.05 [-0.07, 0.17]
Van der Weijden et al. 1996 I	0.8	0.3	20	0.9	0.3	7	2.2%	-0.10 [-0.36, 0.16]
**Subtotal (95% CI)**			**683**			**695**	**77.9%**	**-0.06 [-0.11, -0.00]**
Heterogeneity: Tau² = 0.00; Chi² = 24.26, df = 19 (P = 0.19); I² = 22%								
Test for overall effect: Z = 2.12 (P = 0.03)								
**23.2.2 SS**								
Moritis et al. 2002 I	1.73	0.47	25	1.89	0.47	25	2.1%	-0.16 [-0.42, 0.10]
Re et al. 2015 I	0.66	0.47	40	0.66	0.48	40	3.3%	0.00 [-0.21, 0.21]
**Subtotal (95% CI)**			**65**			**65**	**5.4%**	**-0.06 [-0.22, 0.10]**
Heterogeneity: Tau² = 0.00; Chi² = 0.88, df = 1 (P = 0.35); I² = 0%								
Test for overall effect: Z = 0.75 (P = 0.45)								
**23.2.3 other**								
Khocht et al. 1992 I	1.52	0.36	32	1.58	0.4	31	4.0%	-0.06 [-0.25, 0.13]
Khocht et al. 1992 II	1.47	0.37	32	1.45	0.37	31	4.3%	0.02 [-0.16, 0.20]
Klukowska et al. 2012 I	1.104	0.4017	36	1.196	0.408	36	4.1%	-0.09 [-0.28, 0.10]
Van der Weijden et al. 1996 II	0.8	0.3	20	0.9	0.3	7	2.2%	-0.10 [-0.36, 0.16]
Van der Weijden et al. 1996 III	0.9	0.3	20	0.9	0.3	7	2.2%	0.00 [-0.26, 0.26]
**Subtotal (95% CI)**			**140**			**112**	**16.7%**	**-0.04 [-0.14, 0.05]**
Heterogeneity: Tau² = 0.00; Chi² = 1.04, df = 4 (P = 0.90); I² = 0%								
Test for overall effect: Z = 0.95 (P = 0.34)								
**Total (95% CI)**			**888**			**872**	**100.0%**	**-0.06 [-0.10, -0.02]**
Heterogeneity: Tau² = 0.00; Chi² = 26.28, df = 26 (P = 0.45); I² = 1%								
Test for overall effect: Z = 2.96 (P = 0.003)								
Test for subgroup differences: Chi² = 0.06, df = 2 (P = 0.97), I² = 0%								

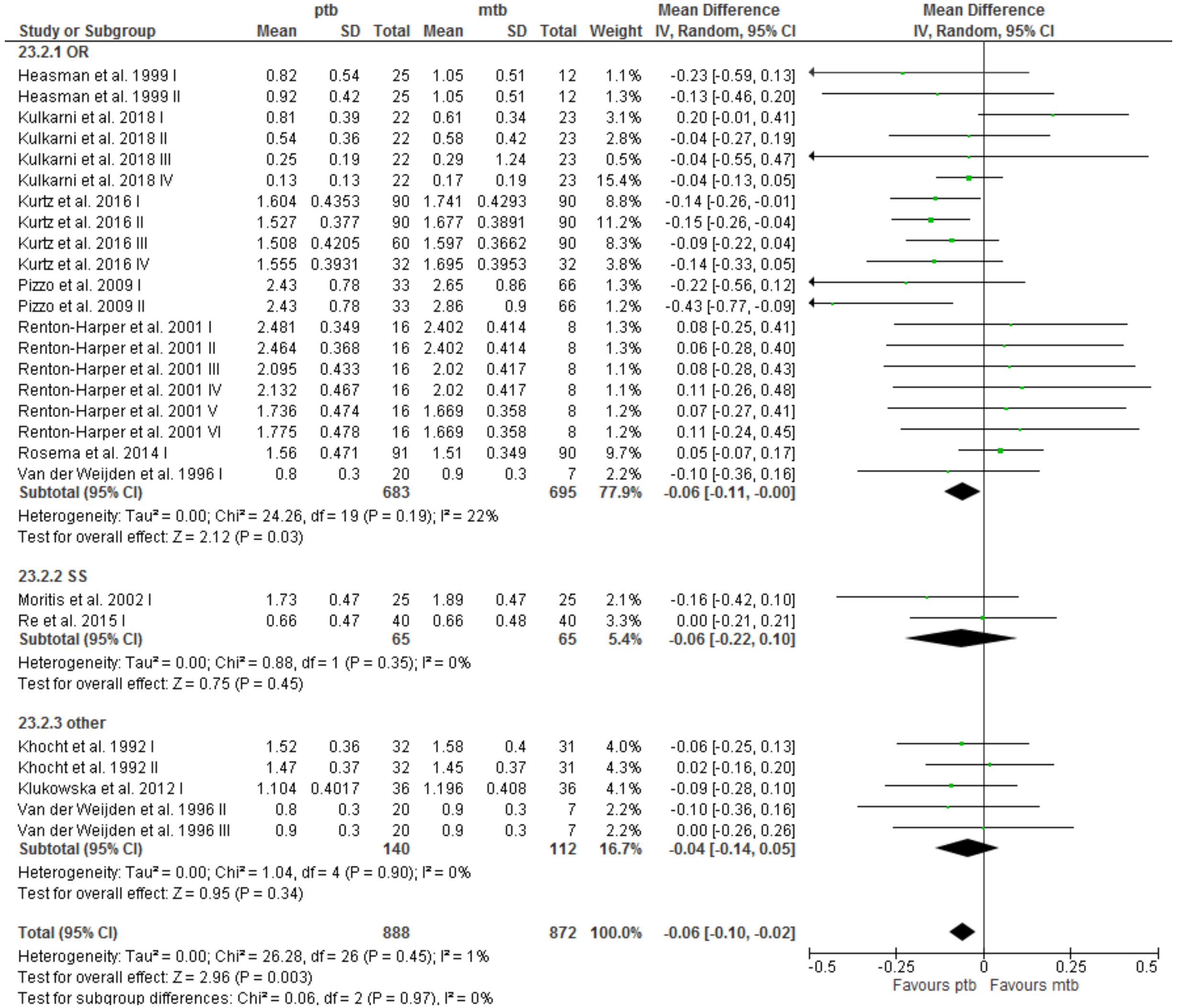

Appendix S8b

Forest plot of the random subanalysis for the OR mode of action for PTB compared to MTB at post-brushing in plaque scores on the Q&HPI

	ptb			mtb				Mean Difference
**Study or Subgroup**	**Mean**	**SD**	**Total**	**Mean**	**SD**	**Total**	**Weight**	**IV, Random, 95% CI**
23.2.1 OR								
Heasman et al. 1999 I	0.82	0.54	25	1.05	0.51	12	2.0%	-0.23 [-0.59, 0.13]
Heasman et al. 1999 II	0.92	0.42	25	1.05	0.51	12	2.3%	-0.13 [-0.46, 0.20]
Kulkarni et al. 2018 I	0.81	0.39	22	0.61	0.34	23	5.0%	0.20 [-0.01, 0.41]
Kulkarni et al. 2018 II	0.54	0.36	22	0.58	0.42	23	4.5%	-0.04 [-0.27, 0.19]
Kulkarni et al. 2018 III	0.25	0.19	22	0.29	1.24	23	1.0%	-0.04 [-0.55, 0.47]
Kulkarni et al. 2018 IV	0.13	0.13	22	0.17	0.19	23	14.3%	-0.04 [-0.13, 0.05]
Kurtz et al. 2016 I	1.604	0.4353	90	1.741	0.4293	90	10.6%	-0.14 [-0.26, -0.01]
Kurtz et al. 2016 II	1.527	0.377	90	1.677	0.3891	90	12.1%	-0.15 [-0.26, -0.04]
Kurtz et al. 2016 III	1.508	0.4205	60	1.597	0.3662	90	10.1%	-0.09 [-0.22, 0.04]
Kurtz et al. 2016 IV	1.555	0.3931	32	1.695	0.3953	32	5.9%	-0.14 [-0.33, 0.05]
Pizzo et al. 2009 I	2.43	0.78	33	2.65	0.86	66	2.3%	-0.22 [-0.56, 0.12]
Pizzo et al. 2009 II	2.43	0.78	33	2.86	0.9	66	2.2%	-0.43 [-0.77, -0.09]
Renton-Harper et al. 2001 I	2.481	0.349	16	2.402	0.414	8	2.3%	0.08 [-0.25, 0.41]
Renton-Harper et al. 2001 II	2.464	0.368	16	2.402	0.414	8	2.2%	0.06 [-0.28, 0.40]
Renton-Harper et al. 2001 III	2.095	0.433	16	2.02	0.417	8	2.0%	0.08 [-0.28, 0.43]
Renton-Harper et al. 2001 IV	2.132	0.467	16	2.02	0.417	8	1.9%	0.11 [-0.26, 0.48]
Renton-Harper et al. 2001 V	1.736	0.474	16	1.669	0.358	8	2.2%	0.07 [-0.27, 0.41]
Renton-Harper et al. 2001 VI	1.775	0.478	16	1.669	0.358	8	2.2%	0.11 [-0.24, 0.45]
Rosema et al. 2014 I	1.56	0.471	91	1.51	0.349	90	11.1%	0.05 [-0.07, 0.17]
Van der Weijden et al. 1996 I	0.8	0.3	20	0.9	0.3	7	3.6%	-0.10 [-0.36, 0.16]
**Subtotal (95% CI)**			**683**			**695**	**100.0%**	**-0.06 [-0.11, -0.00]**
Heterogeneity: Tau² = 0.00; Chi² = 24.26, df = 19 (P = 0.19); I² = 22%								
Test for overall effect: Z = 2.12 (P = 0.03)								
23.2.2 SS								
Moritis et al. 2002 I	1.73	0.47	25	1.89	0.47	25	0.0%	-0.16 [-0.42, 0.10]
Re et al. 2015 I	0.66	0.47	40	0.66	0.48	40	0.0%	0.00 [-0.21, 0.21]
**Subtotal (95% CI)**			**0**			**0**		**Not estimable**
Heterogeneity: Not applicable								
Test for overall effect: Not applicable								
23.2.3 other								
Khocht et al. 1992 I	1.52	0.36	32	1.58	0.4	31	0.0%	-0.06 [-0.25, 0.13]
Khocht et al. 1992 II	1.47	0.37	32	1.45	0.37	31	0.0%	0.02 [-0.16, 0.20]
Klukowska et al. 2012 I	1.104	0.4017	36	1.196	0.408	36	0.0%	-0.09 [-0.28, 0.10]
Van der Weijden et al. 1996 II	0.8	0.3	20	0.9	0.3	7	0.0%	-0.10 [-0.36, 0.16]
Van der Weijden et al. 1996 III	0.9	0.3	20	0.9	0.3	7	0.0%	0.00 [-0.26, 0.26]
**Subtotal (95% CI)**			**0**			**0**		**Not estimable**
Heterogeneity: Not applicable								
Test for overall effect: Not applicable								
**Total (95% CI)**			**683**			**695**	**100.0%**	**-0.06 [-0.11, -0.00]**
Heterogeneity: Tau² = 0.00; Chi² = 24.26, df = 19 (P = 0.19); I² = 22%								
Test for overall effect: Z = 2.12 (P = 0.03)								
Test for subgroup differences: Not applicable								

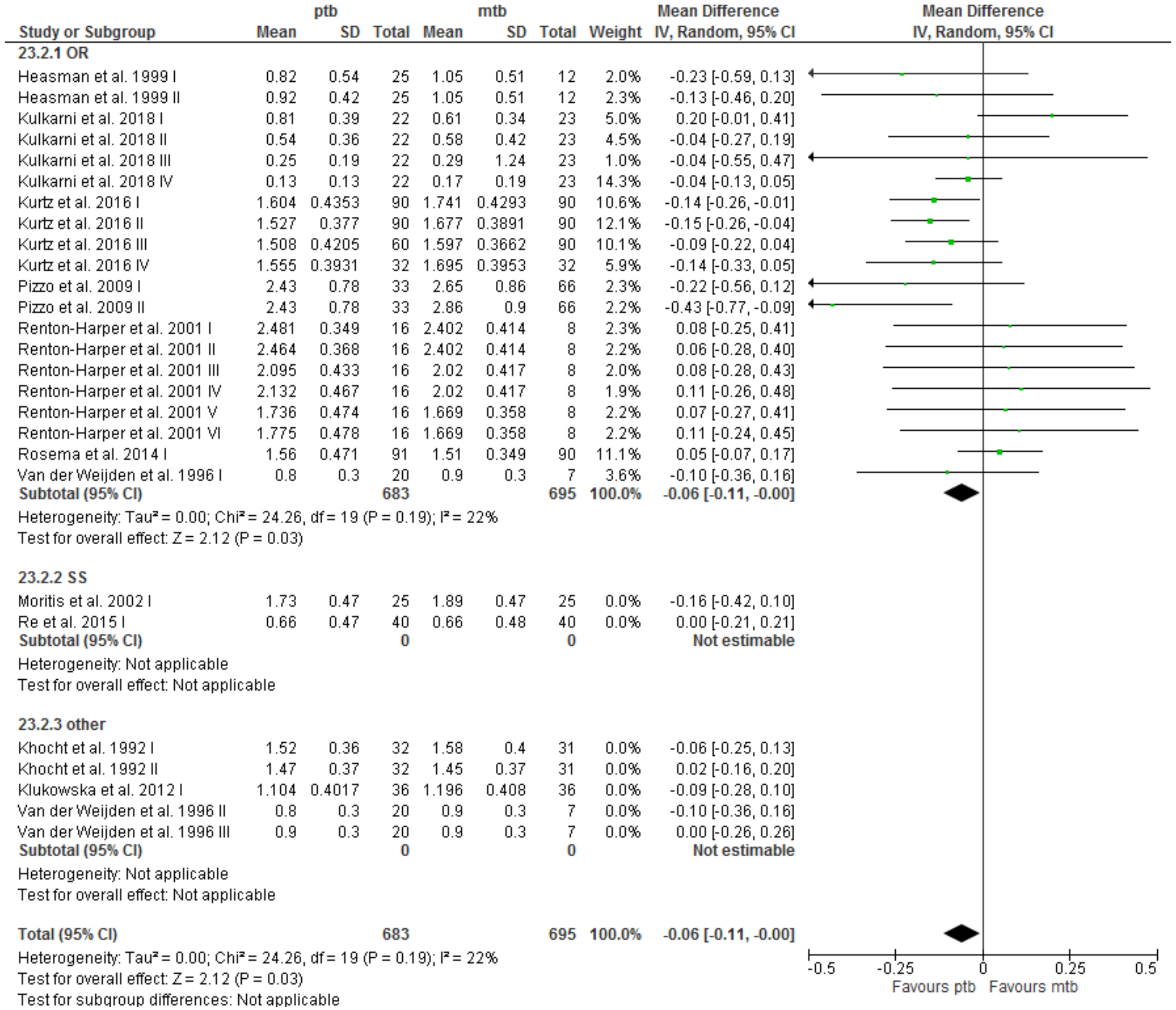

Appendix S8c

Forest plot of the fixed subanalysis for the SS mode of action for PTB compared to MTB at the post-brushing in plaque scores on the Q&HPI

Study or Subgroup	ptb Mean	ptb SD	ptb Total	mtb Mean	mtb SD	mtb Total	Weight	Mean Difference IV, Fixed, 95% CI
**23.2.1 OR**								
Heasman et al. 1999 I	0.82	0.54	25	1.05	0.51	12	0.0%	-0.23 [-0.59, 0.13]
Heasman et al. 1999 II	0.92	0.42	25	1.05	0.51	12	0.0%	-0.13 [-0.46, 0.20]
Kulkarni et al. 2018 I	0.81	0.39	22	0.61	0.34	23	0.0%	0.20 [-0.01, 0.41]
Kulkarni et al. 2018 II	0.54	0.36	22	0.58	0.42	23	0.0%	-0.04 [-0.27, 0.19]
Kulkarni et al. 2018 III	0.25	0.19	22	0.29	1.24	23	0.0%	-0.04 [-0.55, 0.47]
Kulkarni et al. 2018 IV	0.13	0.13	22	0.17	0.19	23	0.0%	-0.04 [-0.13, 0.05]
Kurtz et al. 2016 I	1.604	0.4353	90	1.741	0.4293	90	0.0%	-0.14 [-0.26, -0.01]
Kurtz et al. 2016 II	1.527	0.377	90	1.677	0.3891	90	0.0%	-0.15 [-0.26, -0.04]
Kurtz et al. 2016 III	1.508	0.4205	60	1.597	0.3662	90	0.0%	-0.09 [-0.22, 0.04]
Kurtz et al. 2016 IV	1.555	0.3931	32	1.695	0.3953	32	0.0%	-0.14 [-0.33, 0.05]
Pizzo et al. 2009 I	2.43	0.78	33	2.65	0.86	66	0.0%	-0.22 [-0.56, 0.12]
Pizzo et al. 2009 II	2.43	0.78	33	2.86	0.9	66	0.0%	-0.43 [-0.77, -0.09]
Renton-Harper et al. 2001 I	2.481	0.349	16	2.402	0.414	8	0.0%	0.08 [-0.25, 0.41]
Renton-Harper et al. 2001 II	2.464	0.368	16	2.402	0.414	8	0.0%	0.06 [-0.28, 0.40]
Renton-Harper et al. 2001 III	2.095	0.433	16	2.02	0.417	8	0.0%	0.08 [-0.28, 0.43]
Renton-Harper et al. 2001 IV	2.132	0.467	16	2.02	0.417	8	0.0%	0.11 [-0.26, 0.48]
Renton-Harper et al. 2001 V	1.736	0.474	16	1.669	0.358	8	0.0%	0.07 [-0.27, 0.41]
Renton-Harper et al. 2001 VI	1.775	0.478	16	1.669	0.358	8	0.0%	0.11 [-0.24, 0.45]
Rosema et al. 2014 I	1.56	0.471	91	1.51	0.349	90	0.0%	0.05 [-0.07, 0.17]
Van der Weijden et al. 1996 I	0.8	0.3	20	0.9	0.3	7	0.0%	-0.10 [-0.36, 0.16]
**Subtotal (95% CI)**			**0**			**0**		**Not estimable**
Heterogeneity: Not applicable								
Test for overall effect: Not applicable								
**23.2.2 SS**								
Moritis et al. 2002 I	1.73	0.47	25	1.89	0.47	25	39.0%	-0.16 [-0.42, 0.10]
Re et al. 2015 I	0.66	0.47	40	0.66	0.48	40	61.0%	0.00 [-0.21, 0.21]
**Subtotal (95% CI)**			**65**			**65**	**100.0%**	**-0.06 [-0.22, 0.10]**
Heterogeneity: Chi^2 = 0.88, df = 1 (P = 0.35); I^2 = 0%								
Test for overall effect: Z = 0.75 (P = 0.45)								
**23.2.3 other**								
Khocht et al. 1992 I	1.52	0.36	32	1.58	0.4	31	0.0%	-0.06 [-0.25, 0.13]
Khocht et al. 1992 II	1.47	0.37	32	1.45	0.37	31	0.0%	0.02 [-0.16, 0.20]
Klukowska et al. 2012 I	1.104	0.4017	36	1.196	0.408	36	0.0%	-0.09 [-0.28, 0.10]
Van der Weijden et al. 1996 II	0.8	0.3	20	0.9	0.3	7	0.0%	-0.10 [-0.36, 0.16]
Van der Weijden et al. 1996 III	0.9	0.3	20	0.9	0.3	7	0.0%	0.00 [-0.26, 0.26]
**Subtotal (95% CI)**			**0**			**0**		**Not estimable**
Heterogeneity: Not applicable								
Test for overall effect: Not applicable								
**Total (95% CI)**			**65**			**65**	**100.0%**	**-0.06 [-0.22, 0.10]**
Heterogeneity: Chi^2 = 0.88, df = 1 (P = 0.35); I^2 = 0%								
Test for overall effect: Z = 0.75 (P = 0.45)								
Test for subgroup differences: Not applicable								

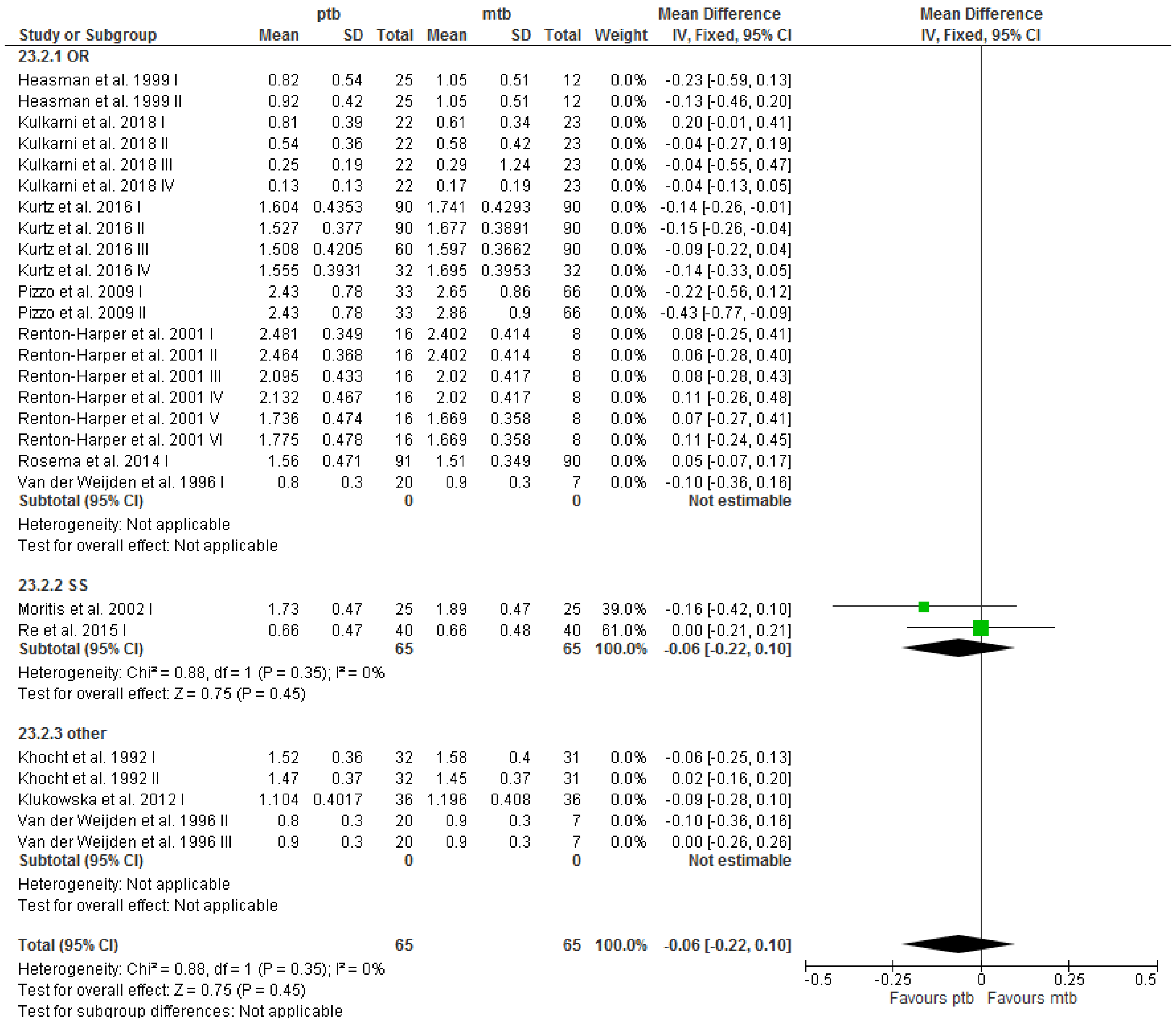

Appendix S8d

Forest plot of the random subanalysis for the OR mode of action by product (P&G) for PTB compared to MTB at post-brushing in plaque scores on the Q&HPI

Study or Subgroup	ptb Mean	ptb SD	ptb Total	mtb Mean	mtb SD	mtb Total	Weight	Mean Difference IV, Random, 95% CI
Heasman et al. 1999 II	0.92	0.42	25	1.05	0.51	12	2.5%	-0.13 [-0.46, 0.20]
Kulkarni et al. 2018 I	0.81	0.39	22	0.61	0.34	23	5.5%	0.20 [-0.01, 0.41]
Kulkarni et al. 2018 II	0.54	0.36	22	0.58	0.42	23	4.9%	-0.04 [-0.27, 0.19]
Kulkarni et al. 2018 III	0.25	0.19	22	0.29	1.24	23	1.1%	-0.04 [-0.55, 0.47]
Kulkarni et al. 2018 IV	0.13	0.13	22	0.17	0.19	23	18.1%	-0.04 [-0.13, 0.05]
Kurtz et al. 2016 I	1.604	0.4353	90	1.741	0.4293	90	12.6%	-0.14 [-0.26, -0.01]
Kurtz et al. 2016 II	1.527	0.377	90	1.677	0.3891	90	14.8%	-0.15 [-0.26, -0.04]
Kurtz et al. 2016 III	1.508	0.4205	60	1.597	0.3662	90	12.0%	-0.09 [-0.22, 0.04]
Kurtz et al. 2016 IV	1.555	0.3931	32	1.695	0.3953	32	6.5%	-0.14 [-0.33, 0.05]
Pizzo et al. 2009 I	2.43	0.78	33	2.65	0.86	66	2.4%	-0.22 [-0.56, 0.12]
Pizzo et al. 2009 II	2.43	0.78	33	2.86	0.9	66	2.3%	-0.43 [-0.77, -0.09]
Renton-Harper et al. 2001 I	2.481	0.349	16	2.402	0.414	8	2.4%	0.08 [-0.25, 0.41]
Renton-Harper et al. 2001 II	2.464	0.368	16	2.402	0.414	8	2.4%	0.06 [-0.28, 0.40]
Renton-Harper et al. 2001 III	2.095	0.433	16	2.02	0.417	8	2.1%	0.08 [-0.28, 0.43]
Renton-Harper et al. 2001 IV	2.132	0.467	16	2.02	0.417	8	2.0%	0.11 [-0.26, 0.48]
Renton-Harper et al. 2001 V	1.736	0.474	16	1.669	0.358	8	2.4%	0.07 [-0.27, 0.41]
Renton-Harper et al. 2001 VI	1.775	0.478	16	1.669	0.358	8	2.3%	0.11 [-0.24, 0.45]
Van der Weijden et al. 1996 I	0.8	0.3	20	0.9	0.3	7	3.9%	-0.10 [-0.36, 0.16]
**Total (95% CI)**			**567**			**593**	**100.0%**	**-0.07 [-0.12, -0.02]**

Heterogeneity: Tau² = 0.00; Chi² = 19.90, df = 17 (P = 0.28); I² = 15%
Test for overall effect: Z = 2.51 (P = 0.01)

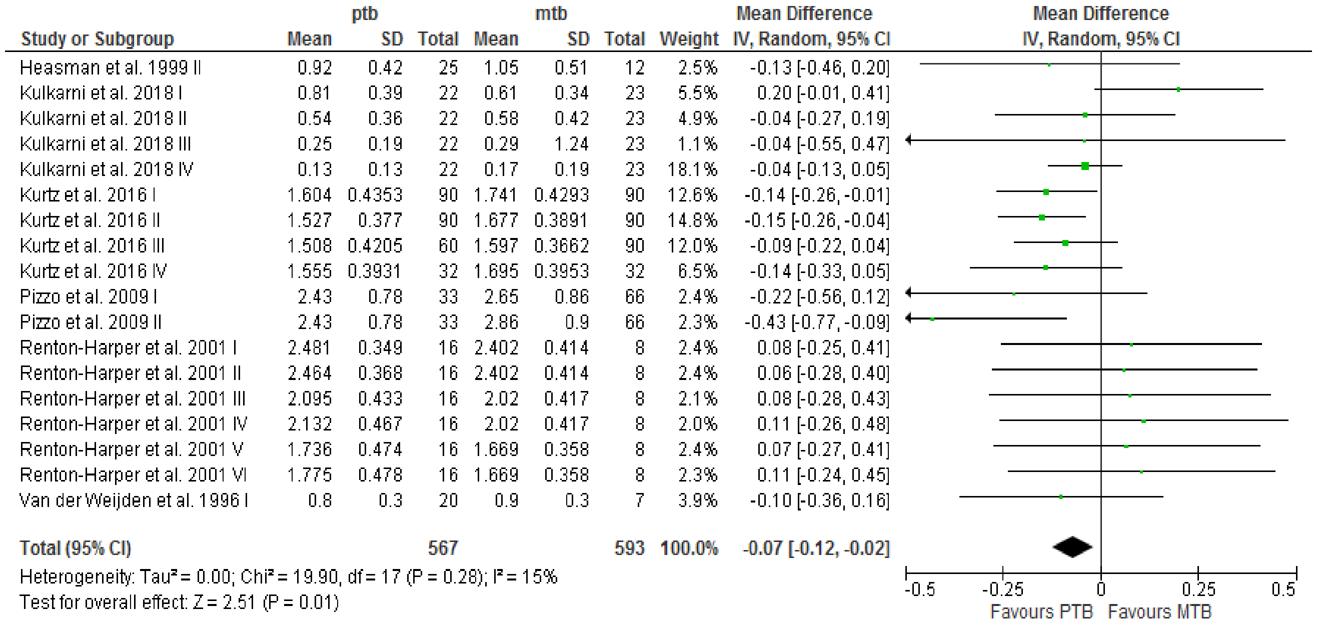

Appendix S9a

Funnel plot for the overall analysis for PTB compared to MTB at post-brushing in plaque scores on the Q&HPI

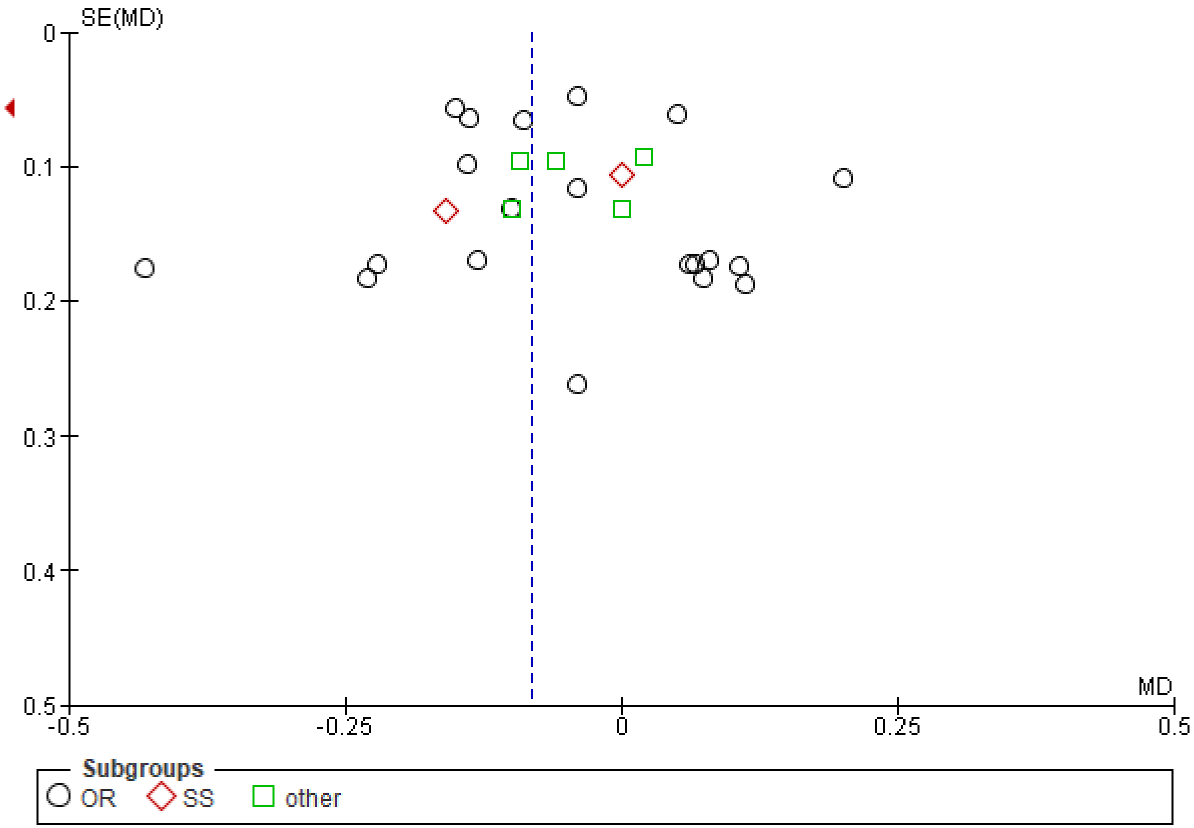

Appendix S9b

Funnel plot for the random subanalysis for the OR mode of action for PTB compared to MTB at post-brushing in plaque scores on the Q&HPI

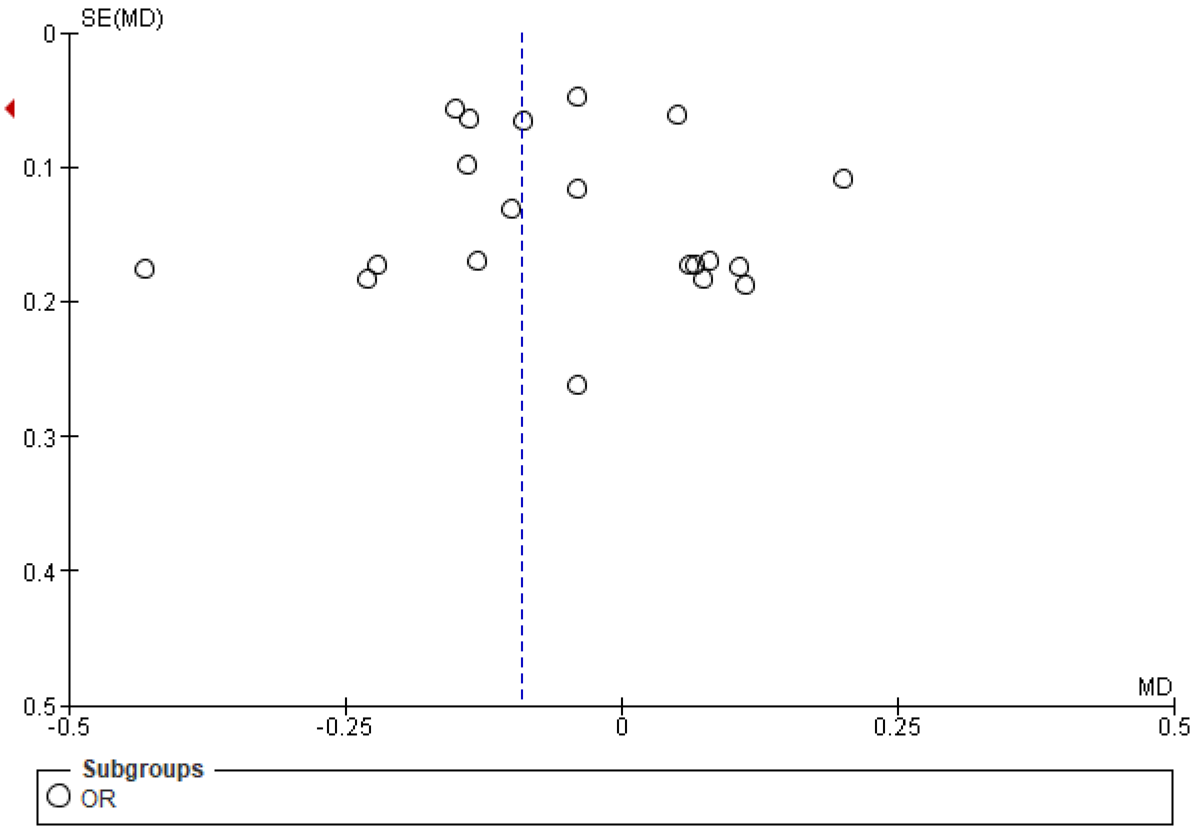

Appendix S9c

Funnel plot of the random subanalysis for the OR mode of action by product (P&G) for PTB compared to MTB at post-brushing in plaque scores on the Q&HPI

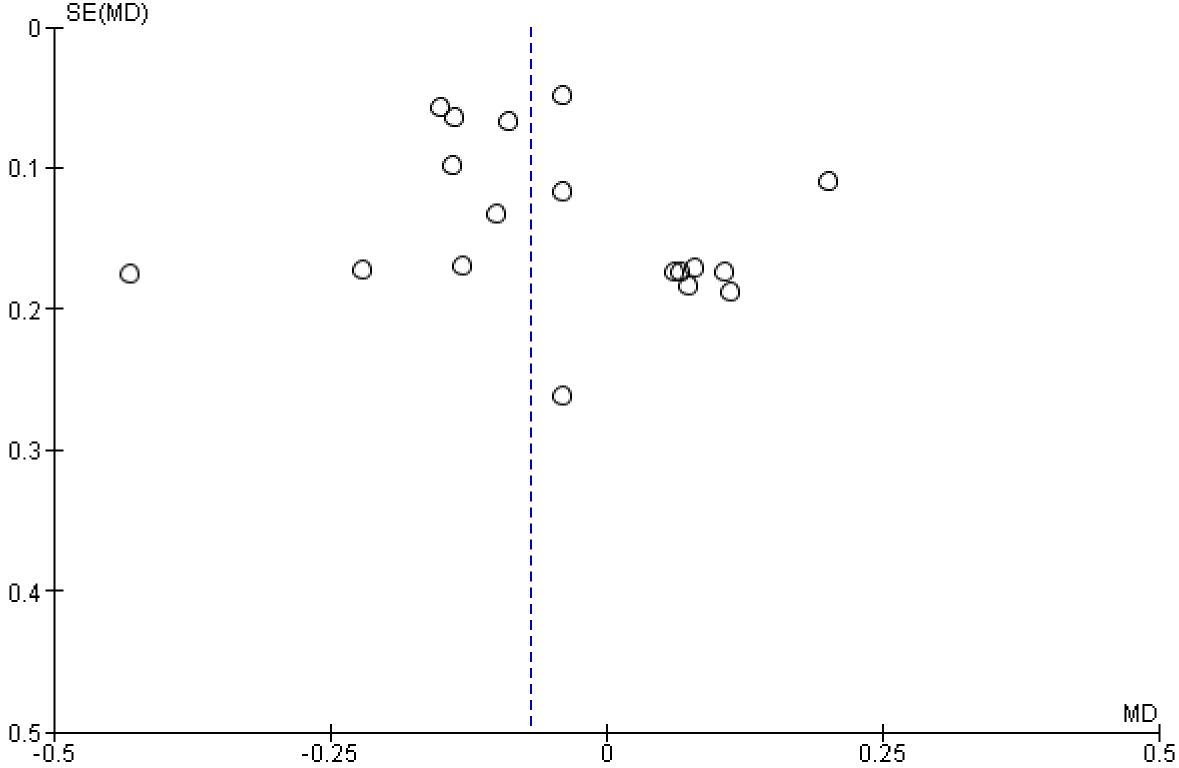

Appendix S10a

Forest plot of the overall analysis for PTB compared to MTB at the change in plaque scores on the Q&HPI

	ptb			mtb				Mean Difference
**Study or Subgroup**	**Mean**	**SD**	**Total**	**Mean**	**SD**	**Total**	**Weight**	**IV, Random, 95% CI**
**23.3.1 OR**								
Kulkarni et al. 2018 I	-0.9568	0.4233	22	-0.9461	0.4066	23	3.2%	-0.01 [-0.25, 0.23]
Kulkarni et al. 2018 II	-0.8845	0.3938	22	-0.957	0.4088	23	3.4%	0.07 [-0.16, 0.31]
Kulkarni et al. 2018 III	-0.7246	0.3701	22	-0.5922	0.3236	23	4.2%	-0.13 [-0.34, 0.07]
Kulkarni et al. 2018 IV	-0.5664	0.4223	22	-0.537	0.2619	23	4.1%	-0.03 [-0.24, 0.18]
Kurtz et al. 2016 I	-0.726	0.2996	90	-0.5666	0.2429	90	10.2%	-0.16 [-0.24, -0.08]
Kurtz et al. 2016 II	-0.748	0.2568	90	-0.594	0.2158	90	10.9%	-0.15 [-0.22, -0.08]
Kurtz et al. 2016 III	-0.817	0.2705	60	-0.713	0.249	60	9.3%	-0.10 [-0.20, -0.01]
Kurtz et al. 2016 IV	-0.649	0.247	32	-0.473	0.2117	32	8.1%	-0.18 [-0.29, -0.06]
Pizzo et al. 2009 I	-0.97	0.43	33	-0.78	0.38	66	5.2%	-0.19 [-0.36, -0.02]
Pizzo et al. 2009 II	-0.97	0.43	33	-0.49	0.33	66	5.4%	-0.48 [-0.65, -0.31]
Rosema et al. 2014 I	-1.24	0.301	91	-1.05	0.268	90	10.0%	-0.19 [-0.27, -0.11]
**Subtotal (95% CI)**			**517**			**586**	**73.9%**	**-0.16 [-0.22, -0.10]**
Heterogeneity: Tau² = 0.00; Chi² = 23.07, df = 10 (P = 0.01); I² = 57%								
Test for overall effect: Z = 5.32 (P < 0.00001)								
**23.3.2 SS**								
Re et al. 2015 I	-1.19	0.37	40	-1.05	0.22	40	6.9%	-0.14 [-0.27, -0.01]
**Subtotal (95% CI)**			**40**			**40**	**6.9%**	**-0.14 [-0.27, -0.01]**
Heterogeneity: Not applicable								
Test for overall effect: Z = 2.06 (P = 0.04)								
**23.3.3 Other**								
Khocht et al. 1992 I	-0.75	0.41	32	-0.62	0.35	31	4.6%	-0.13 [-0.32, 0.06]
Khocht et al. 1992 II	-0.37	0.28	32	-0.4	0.32	31	6.2%	0.03 [-0.12, 0.18]
Klukowska et al. 2012 I	-1.042	0.2174	36	-0.973	0.2516	36	8.3%	-0.07 [-0.18, 0.04]
**Subtotal (95% CI)**			**100**			**98**	**19.1%**	**-0.05 [-0.13, 0.03]**
Heterogeneity: Tau² = 0.00; Chi² = 1.92, df = 2 (P = 0.38); I² = 0%								
Test for overall effect: Z = 1.27 (P = 0.20)								
**Total (95% CI)**			**657**			**724**	**100.0%**	**-0.14 [-0.19, -0.09]**
Heterogeneity: Tau² = 0.00; Chi² = 31.00, df = 14 (P = 0.006); I² = 55%								
Test for overall effect: Z = 5.36 (P < 0.00001)								
Test for subgroup differences: Chi² = 4.51, df = 2 (P = 0.11), I² = 55.6%								

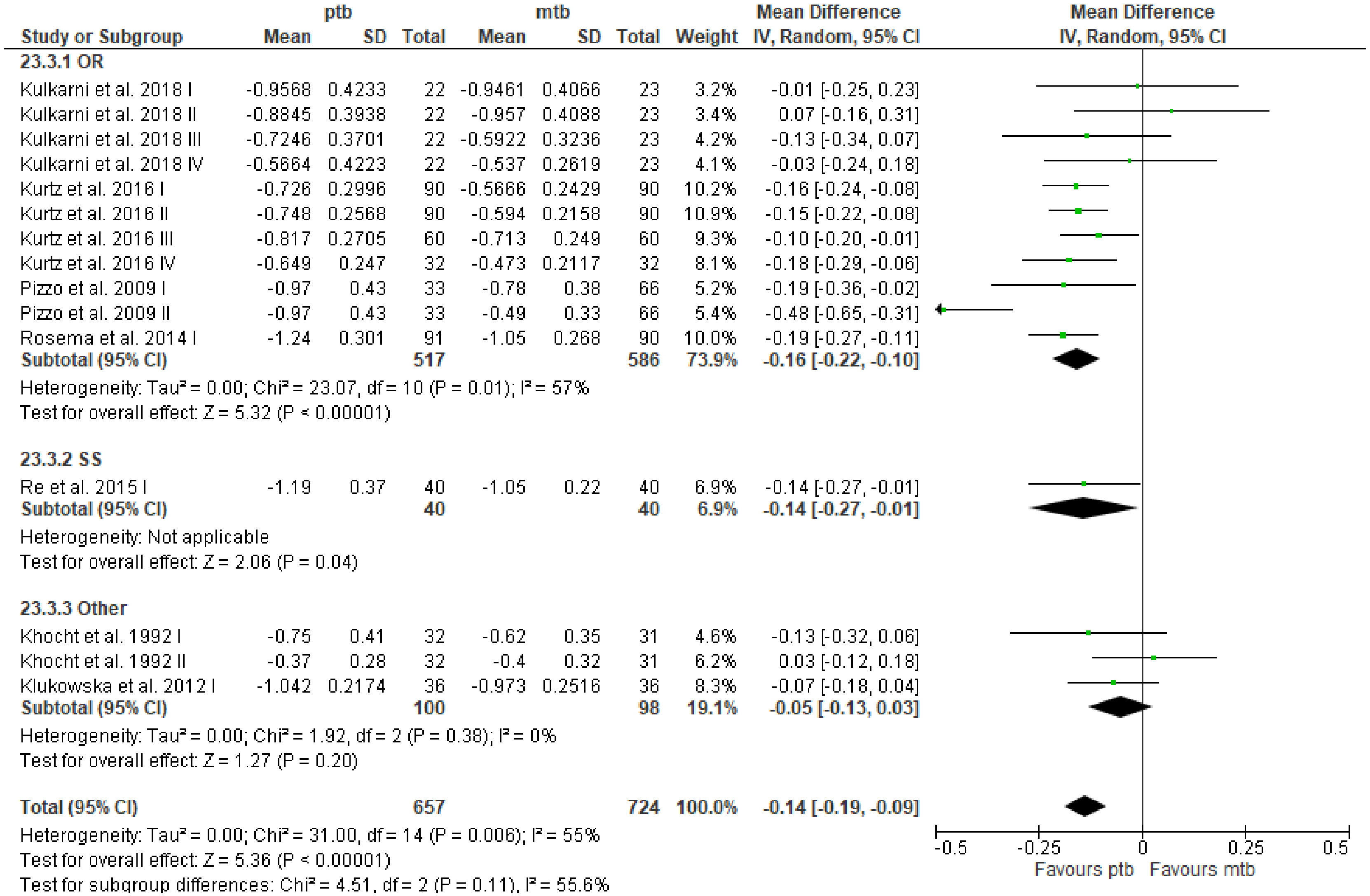

Appendix S10b

Forest plot of the random subanalysis for the OR mode of action for PTB compared to MTB at the change in plaque scores on the Q&HPI

	ptb			mtb				Mean Difference
Study or Subgroup	Mean	SD	Total	Mean	SD	Total	Weight	IV, Random, 95% CI
**23.3.1 OR**								
Kulkarni et al. 2018 I	-0.9568	0.4233	22	-0.9461	0.4066	23	4.4%	-0.01 [-0.25, 0.23]
Kulkarni et al. 2018 II	-0.8845	0.3938	22	-0.957	0.4088	23	4.6%	0.07 [-0.16, 0.31]
Kulkarni et al. 2018 III	-0.7246	0.3701	22	-0.5922	0.3236	23	5.7%	-0.13 [-0.34, 0.07]
Kulkarni et al. 2018 IV	-0.5664	0.4223	22	-0.537	0.2619	23	5.5%	-0.03 [-0.24, 0.18]
Kurtz et al. 2016 I	-0.726	0.2996	90	-0.5666	0.2429	90	13.8%	-0.16 [-0.24, -0.08]
Kurtz et al. 2016 II	-0.748	0.2568	90	-0.594	0.2158	90	14.7%	-0.15 [-0.22, -0.08]
Kurtz et al. 2016 III	-0.817	0.2705	60	-0.713	0.249	60	12.6%	-0.10 [-0.20, -0.01]
Kurtz et al. 2016 IV	-0.649	0.247	32	-0.473	0.2117	32	10.9%	-0.18 [-0.29, -0.06]
Pizzo et al. 2009 I	-0.97	0.43	33	-0.78	0.38	66	7.0%	-0.19 [-0.36, -0.02]
Pizzo et al. 2009 II	-0.97	0.43	33	-0.49	0.33	66	7.3%	-0.48 [-0.65, -0.31]
Rosema et al. 2014 I	-1.24	0.301	91	-1.05	0.268	90	13.5%	-0.19 [-0.27, -0.11]
**Subtotal (95% CI)**			**517**			**586**	**100.0%**	**-0.16 [-0.22, -0.10]**
Heterogeneity: Tau² = 0.00; Chi² = 23.07, df = 10 (P = 0.01); I² = 57%								
Test for overall effect: Z = 5.32 (P < 0.00001)								
**23.3.2 SS**								
Re et al. 2015 I	-1.19	0.37	40	-1.05	0.22	40	0.0%	-0.14 [-0.27, -0.01]
**Subtotal (95% CI)**			**0**			**0**		**Not estimable**
Heterogeneity: Not applicable								
Test for overall effect: Not applicable								
**23.3.3 Other**								
Khocht et al. 1992 I	-0.75	0.41	32	-0.62	0.35	31	0.0%	-0.13 [-0.32, 0.06]
Khocht et al. 1992 II	-0.37	0.28	32	-0.4	0.32	31	0.0%	0.03 [-0.12, 0.18]
Klukowska et al. 2012 I	-1.042	0.2174	36	-0.973	0.2516	36	0.0%	-0.07 [-0.18, 0.04]
**Subtotal (95% CI)**			**0**			**0**		**Not estimable**
Heterogeneity: Not applicable								
Test for overall effect: Not applicable								
**Total (95% CI)**			**517**			**586**	**100.0%**	**-0.16 [-0.22, -0.10]**
Heterogeneity: Tau² = 0.00; Chi² = 23.07, df = 10 (P = 0.01); I² = 57%								
Test for overall effect: Z = 5.32 (P < 0.00001)								
Test for subgroup differences: Not applicable								

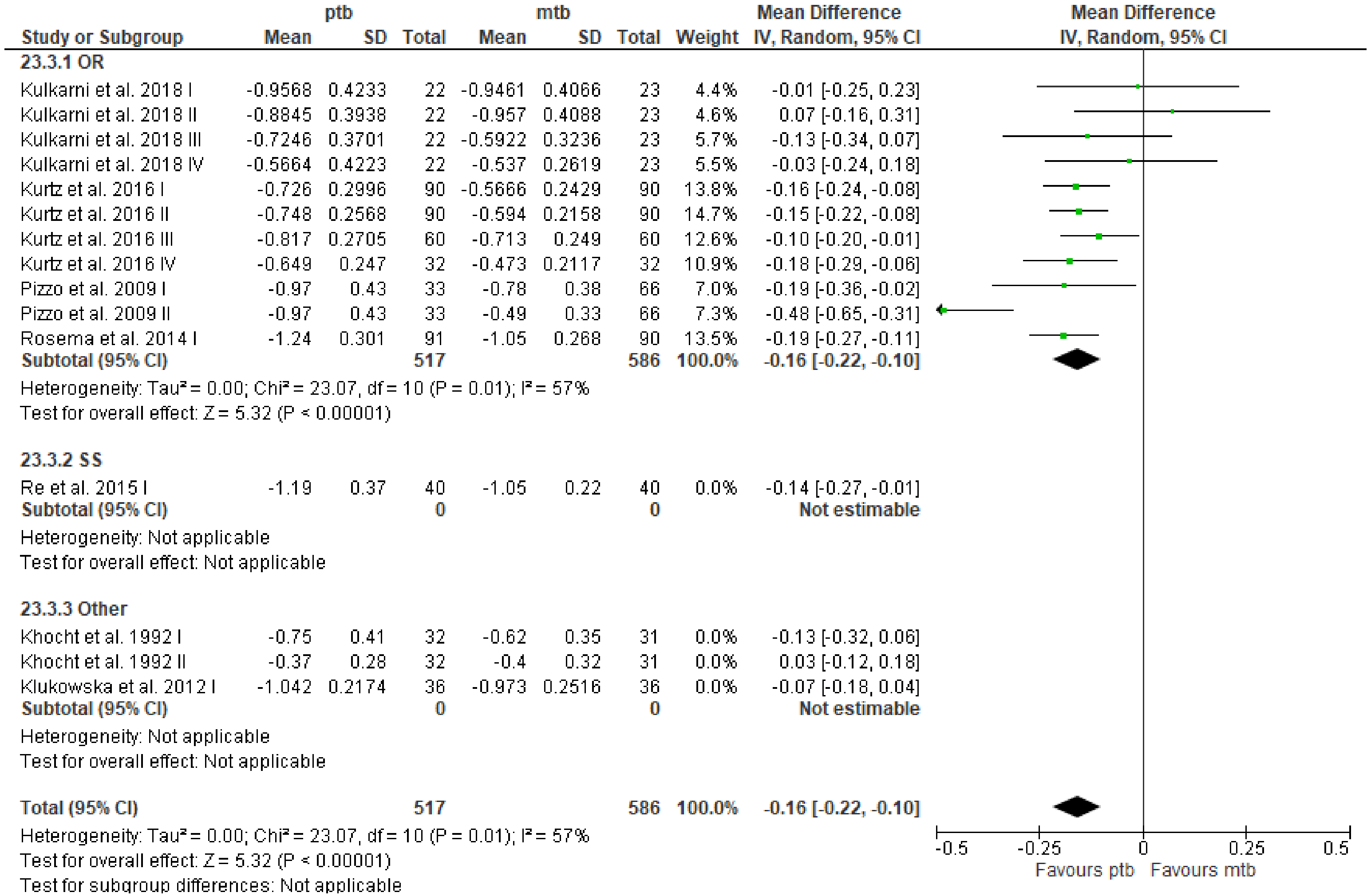

Appendix S10c

Forest plot of the random subanalysis for the OR mode of action by product (P&G) for PTB compared to MTB at the change in plaque scores on the Q&HPI

	ptb			mtb				Mean Difference
Study or Subgroup	Mean	SD	Total	Mean	SD	Total	Weight	IV, Random, 95% CI
Kulkarni et al. 2018 I	-0.9568	0.4233	22	-0.9461	0.4066	23	5.5%	-0.01 [-0.25, 0.23]
Kulkarni et al. 2018 II	-0.8845	0.3938	22	-0.957	0.4088	23	5.7%	0.07 [-0.16, 0.31]
Kulkarni et al. 2018 III	-0.7246	0.3701	22	-0.5922	0.3236	23	6.9%	-0.13 [-0.34, 0.07]
Kulkarni et al. 2018 IV	-0.5664	0.4223	22	-0.537	0.2619	23	6.8%	-0.03 [-0.24, 0.18]
Kurtz et al. 2016 I	-0.726	0.2996	90	-0.5666	0.2429	90	15.2%	-0.16 [-0.24, -0.08]
Kurtz et al. 2016 II	-0.748	0.2568	90	-0.594	0.2158	90	16.0%	-0.15 [-0.22, -0.08]
Kurtz et al. 2016 III	-0.817	0.2705	60	-0.713	0.249	60	14.1%	-0.10 [-0.20, -0.01]
Kurtz et al. 2016 IV	-0.649	0.247	32	-0.473	0.2117	32	12.5%	-0.18 [-0.29, -0.06]
Pizzo et al. 2009 I	-0.97	0.43	33	-0.78	0.38	66	8.4%	-0.19 [-0.36, -0.02]
Pizzo et al. 2009 II	-0.97	0.43	33	-0.49	0.33	66	8.8%	-0.48 [-0.65, -0.31]
**Total (95% CI)**			**426**			**496**	**100.0%**	**-0.15 [-0.22, -0.08]**

Heterogeneity: Tau² = 0.01; Chi² = 22.46, df = 9 (P = 0.008); I² = 60%
Test for overall effect: Z = 4.44 (P < 0.00001)

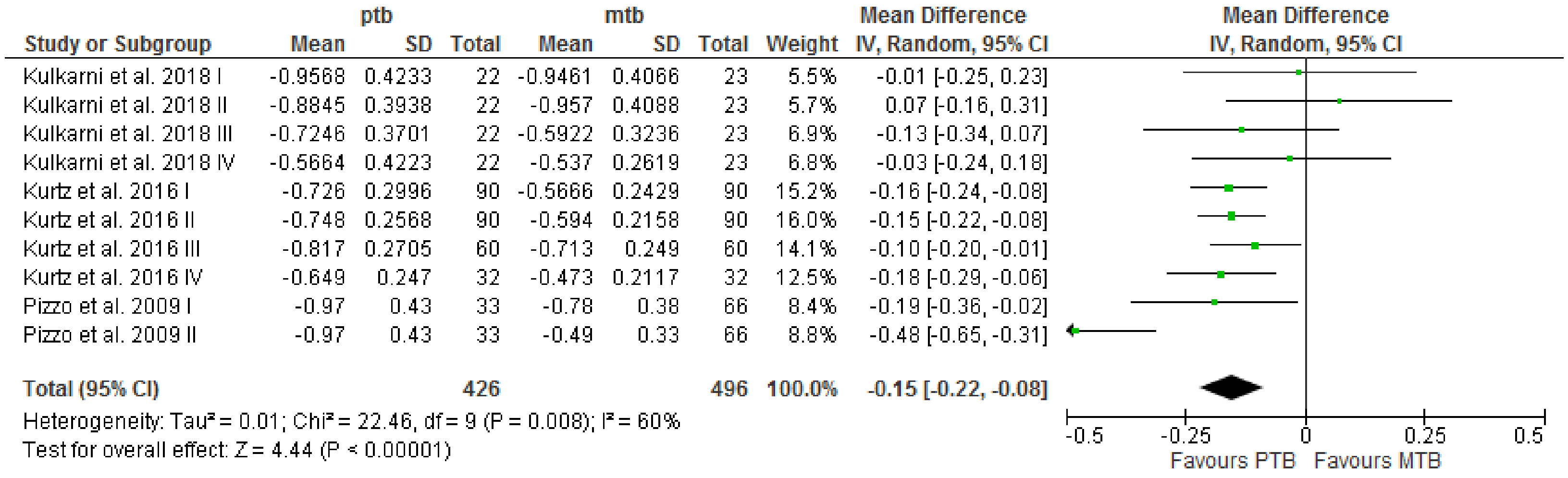

Appendix S11a

Funnel plot of the overall analysis for PTB compared to MTB at the change in plaque scores on the Q&HPI

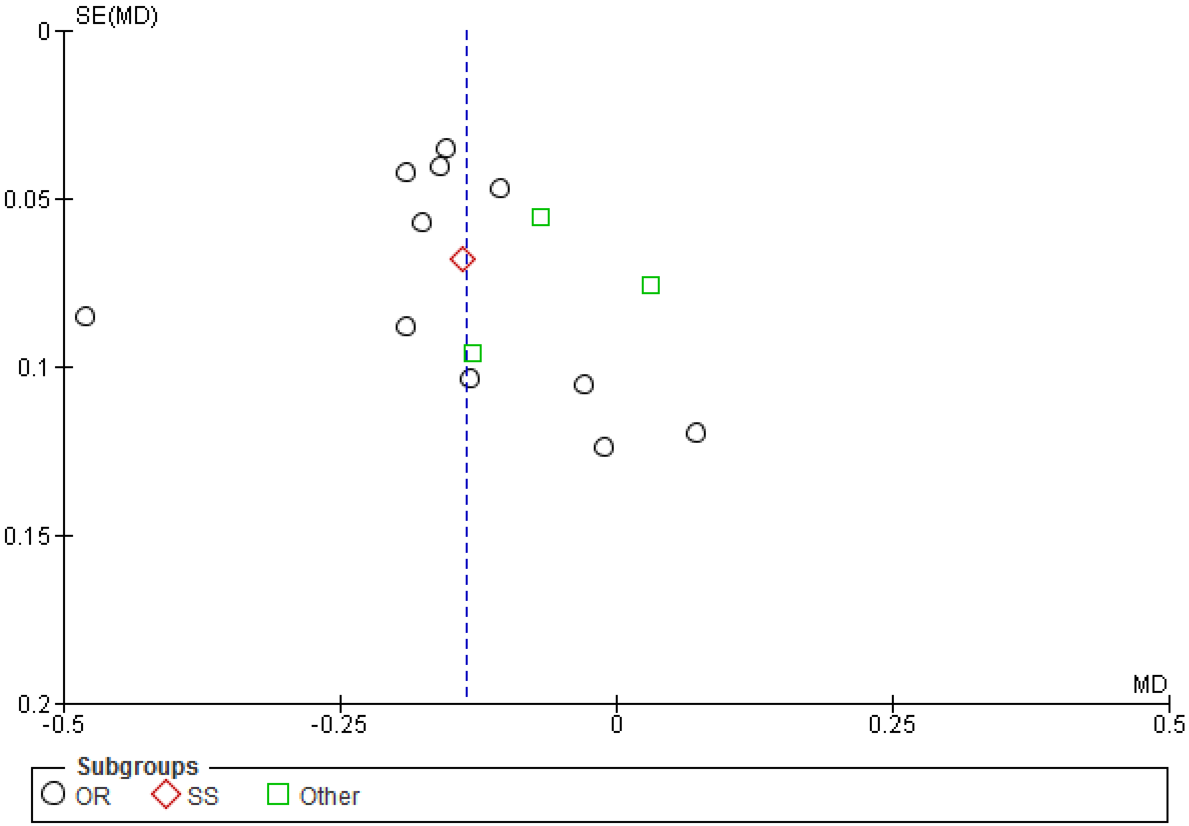

Appendix S11b

Funnel plot of the random subanalysis for the OR mode of action for PTB compared to MTB at the change in plaque scores on the Q&HPI

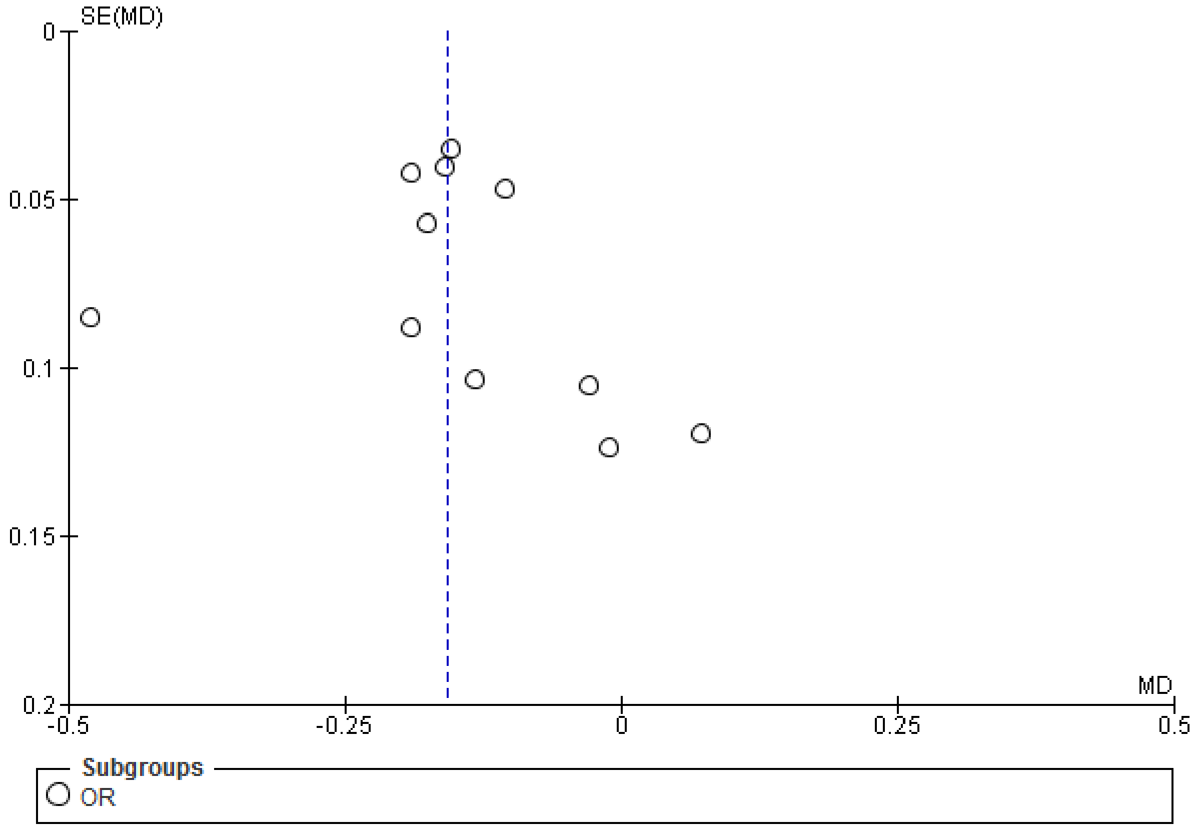

Appendix S11c

Funnel plot of the random subanalysis for the OR mode of action by product (P&G) for PTB compared to MTB at the change in plaque scores on the Q&HPI

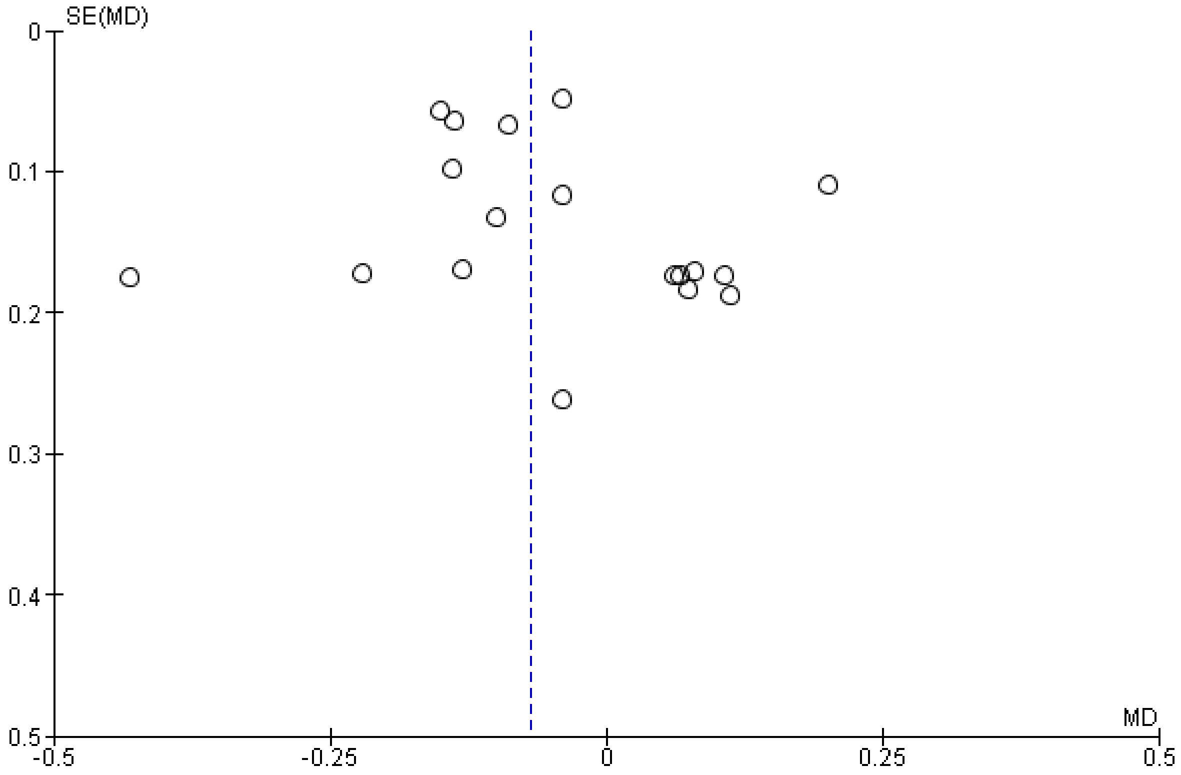

Appendix S12a

Forest plot of the overall analysis for PTB compared to MTB at pre-brushing in plaque scores on the RMNPI

Study or Subgroup	PTB Mean	PTB SD	PTB Total	MTB Mean	MTB SD	MTB Total	Weight	Mean Difference IV, Random, 95% CI
**24.1.1 OR**								
Sharma et al. 2011 I	0.632	0.0223	40	0.625	0.0264	40	44.8%	0.01 [-0.00, 0.02]
**Subtotal (95% CI)**			**40**			**40**	**44.8%**	**0.01 [-0.00, 0.02]**
Heterogeneity: Not applicable								
Test for overall effect: Z = 1.28 (P = 0.20)								
**24.1.2 SS**								
Gallob et al. 2015 I	0.66	0.04	39	0.65	0.04	40	16.5%	0.01 [-0.01, 0.03]
Nathoo et al. 2012 I	0.75	0.09	40	0.74	0.09	36	3.1%	0.01 [-0.03, 0.05]
Nathoo et al. 2014 I	0.71	0.1	40	0.72	0.14	20	1.1%	-0.01 [-0.08, 0.06]
Nathoo et al. 2014 II	0.75	0.11	40	0.75	0.14	20	1.0%	0.00 [-0.07, 0.07]
Sharma et al. 2006 I	0.66	0.04	35	0.65	0.03	12	11.1%	0.01 [-0.01, 0.03]
Sharma et al. 2006 II	0.66	0.04	36	0.65	0.03	12	11.2%	0.01 [-0.01, 0.03]
Sharma et al. 2006 III	0.66	0.04	36	0.65	0.03	12	11.2%	0.01 [-0.01, 0.03]
**Subtotal (95% CI)**			**266**			**152**	**55.2%**	**0.01 [-0.00, 0.02]**
Heterogeneity: Tau² = 0.00; Chi² = 0.39, df = 6 (P = 1.00); I² = 0%								
Test for overall effect: Z = 1.91 (P = 0.06)								
**Total (95% CI)**			**306**			**192**	**100.0%**	**0.01 [0.00, 0.02]**
Heterogeneity: Tau² = 0.00; Chi² = 0.50, df = 7 (P = 1.00); I² = 0%								
Test for overall effect: Z = 2.28 (P = 0.02)								
Test for subgroup differences: Chi² = 0.11, df = 1 (P = 0.74), I² = 0%								

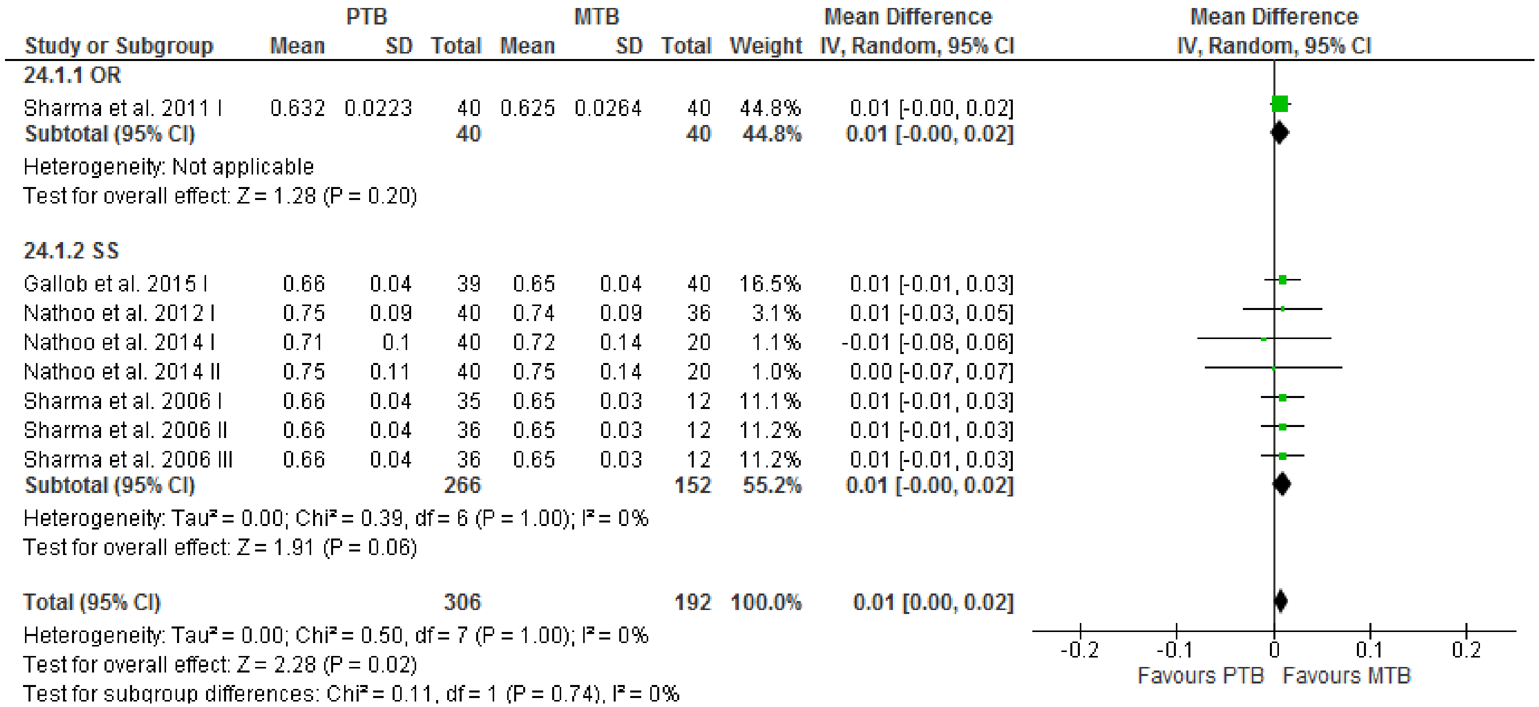

Appendix S12b

Forest plot of the random subanalysis for the SS mode of action for PTB compared to MTB at pre-brushing in plaque scores on the RMNPI

	PTB			MTB				Mean Difference
**Study or Subgroup**	**Mean**	**SD**	**Total**	**Mean**	**SD**	**Total**	**Weight**	**IV, Random, 95% CI**
**24.1.1 OR**								
Sharma et al. 2011 I	0.632	0.0223	40	0.625	0.0264	40	0.0%	0.01 [-0.00, 0.02]
**Subtotal (95% CI)**			**0**			**0**		**Not estimable**
Heterogeneity: Not applicable								
Test for overall effect: Not applicable								
**24.1.2 SS**								
Gallob et al. 2015 I	0.66	0.04	39	0.65	0.04	40	29.9%	0.01 [-0.01, 0.03]
Nathoo et al. 2012 I	0.75	0.09	40	0.74	0.09	36	5.7%	0.01 [-0.03, 0.05]
Nathoo et al. 2014 I	0.71	0.1	40	0.72	0.14	20	2.0%	-0.01 [-0.08, 0.06]
Nathoo et al. 2014 II	0.75	0.11	40	0.75	0.14	20	1.9%	0.00 [-0.07, 0.07]
Sharma et al. 2006 I	0.66	0.04	35	0.65	0.03	12	20.1%	0.01 [-0.01, 0.03]
Sharma et al. 2006 II	0.66	0.04	36	0.65	0.03	12	20.3%	0.01 [-0.01, 0.03]
Sharma et al. 2006 III	0.66	0.04	36	0.65	0.03	12	20.3%	0.01 [-0.01, 0.03]
**Subtotal (95% CI)**			**266**			**152**	**100.0%**	**0.01 [-0.00, 0.02]**
Heterogeneity: $Tau^2 = 0.00$; $Chi^2 = 0.39$, df = 6 (P = 1.00); $I^2 = 0\%$								
Test for overall effect: Z = 1.91 (P = 0.06)								
**Total (95% CI)**			**266**			**152**	**100.0%**	**0.01 [-0.00, 0.02]**
Heterogeneity: $Tau^2 = 0.00$; $Chi^2 = 0.39$, df = 6 (P = 1.00); $I^2 = 0\%$								
Test for overall effect: Z = 1.91 (P = 0.06)								
Test for subgroup differences: Not applicable								

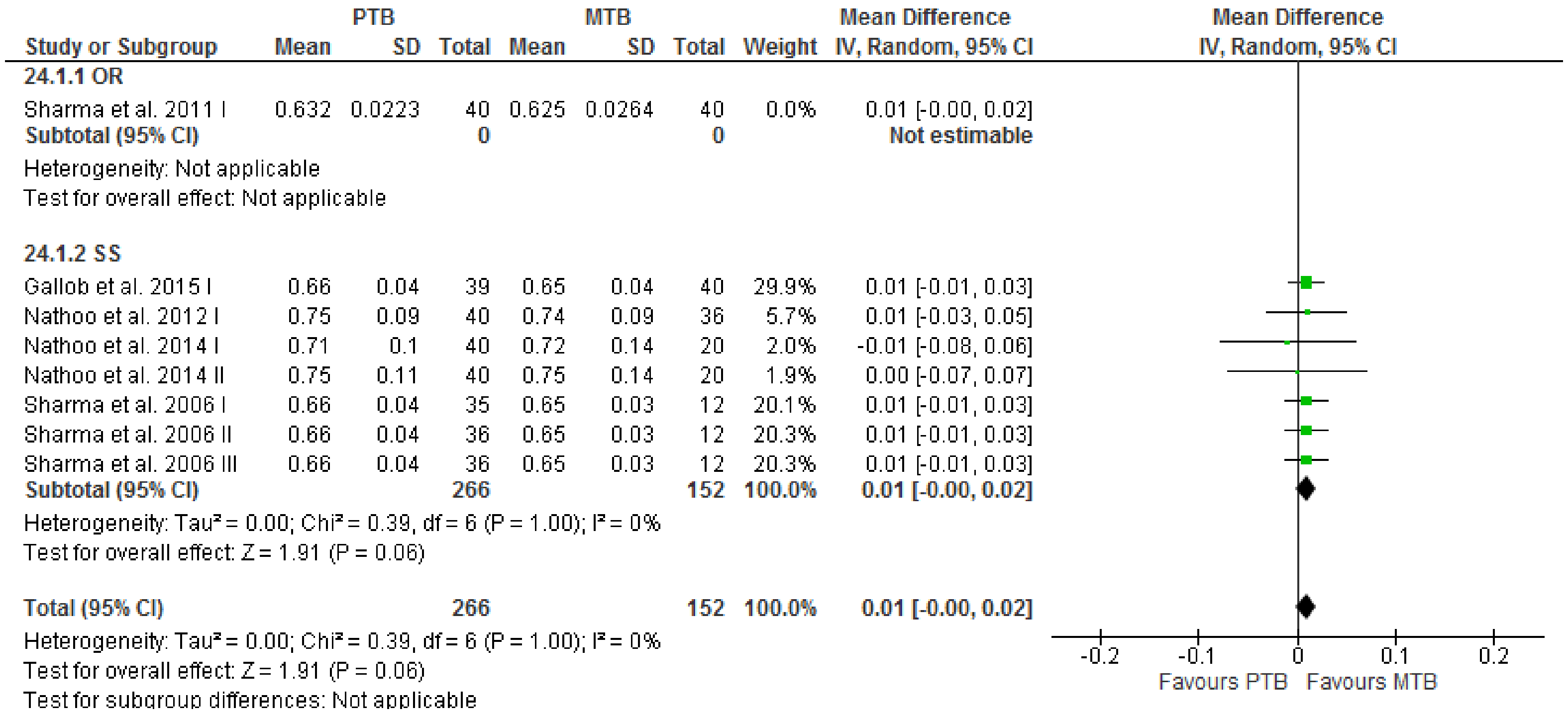

Appendix S12c

Forest plot of the random subanalysis for the SS mode of action by product (Colgate) for PTB compared to MTB at pre-brushing in plaque scores on the RMNPI

	PTB			MTB				Mean Difference
**Study or Subgroup**	**Mean**	**SD**	**Total**	**Mean**	**SD**	**Total**	**Weight**	**IV, Fixed, 95% CI**
Nathoo et al. 2012 I	0.75	0.09	40	0.74	0.09	36	59.5%	0.01 [-0.03, 0.05]
Nathoo et al. 2014 I	0.71	0.1	40	0.72	0.14	20	20.7%	-0.01 [-0.08, 0.06]
Nathoo et al. 2014 II	0.75	0.11	40	0.75	0.14	20	19.8%	0.00 [-0.07, 0.07]
**Total (95% CI)**			**120**			**76**	**100.0%**	**0.00 [-0.03, 0.04]**

Heterogeneity: Chi² = 0.26, df = 2 (P = 0.88); I² = 0%
Test for overall effect: Z = 0.24 (P = 0.81)

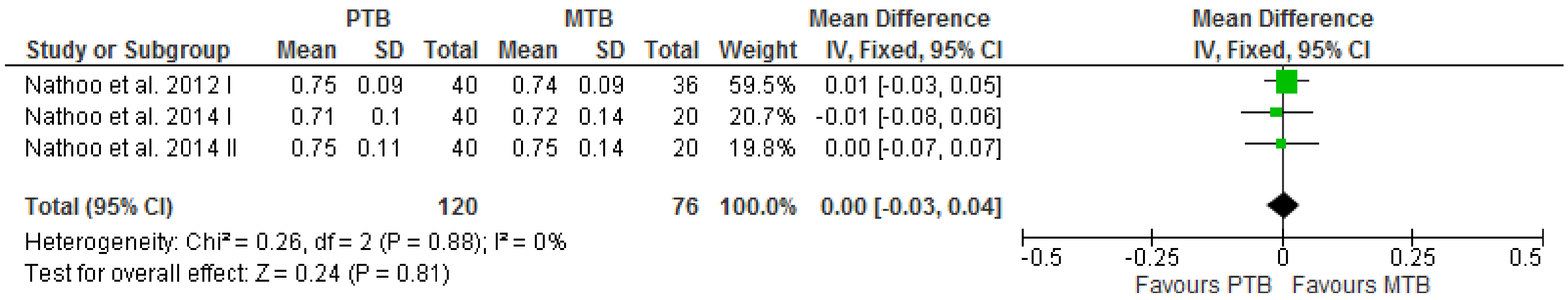

Appendix S13a

Forest plot of the overall analysis for PTB compared to MTB at post-brushing in plaque scores on the RMNPI

	PTB			MTB				Mean Difference
**Study or Subgroup**	**Mean**	**SD**	**Total**	**Mean**	**SD**	**Total**	**Weight**	**IV, Random, 95% CI**
**24.2.1 OR**								
Sharma et al. 2011 I	0.076	0.026	40	0.195	0.0557	40	14.4%	-0.12 [-0.14, -0.10]
**Subtotal (95% CI)**			**40**			**40**	**14.4%**	**-0.12 [-0.14, -0.10]**
Heterogeneity: Not applicable								
Test for overall effect: Z = 12.24 (P < 0.00001)								
**24.2.2 SS**								
Gallob et al. 2015 I	0.27	0.07	39	0.41	0.09	40	13.2%	-0.14 [-0.18, -0.10]
Nathoo et al. 2012 I	0.35	0.08	40	0.49	0.08	36	13.2%	-0.14 [-0.18, -0.10]
Nathoo et al. 2014 I	0.3	0.1	40	0.35	0.08	20	12.1%	-0.05 [-0.10, -0.00]
Nathoo et al. 2014 II	0.32	0.1	40	0.45	0.13	20	10.3%	-0.13 [-0.19, -0.07]
Sharma et al. 2006 I	0.16	0.09	35	0.14	0.07	12	11.9%	0.02 [-0.03, 0.07]
Sharma et al. 2006 II	0.11	0.06	36	0.14	0.07	12	12.4%	-0.03 [-0.07, 0.01]
Sharma et al. 2006 III	0.07	0.05	36	0.14	0.07	12	12.5%	-0.07 [-0.11, -0.03]
**Subtotal (95% CI)**			**266**			**152**	**85.6%**	**-0.08 [-0.12, -0.03]**
Heterogeneity: Tau² = 0.00; Chi² = 45.82, df = 6 (P < 0.00001); I² = 87%								
Test for overall effect: Z = 3.30 (P = 0.0010)								
**24.2.3 other**								
**Subtotal (95% CI)**			**0**			**0**		**Not estimable**
Heterogeneity: Not applicable								
Test for overall effect: Not applicable								
**Total (95% CI)**			**306**			**192**	**100.0%**	**-0.08 [-0.12, -0.05]**
Heterogeneity: Tau² = 0.00; Chi² = 52.67, df = 7 (P < 0.00001); I² = 87%								
Test for overall effect: Z = 4.41 (P < 0.0001)								
Test for subgroup differences: Chi² = 2.66, df = 1 (P = 0.10), I² = 62.4%								

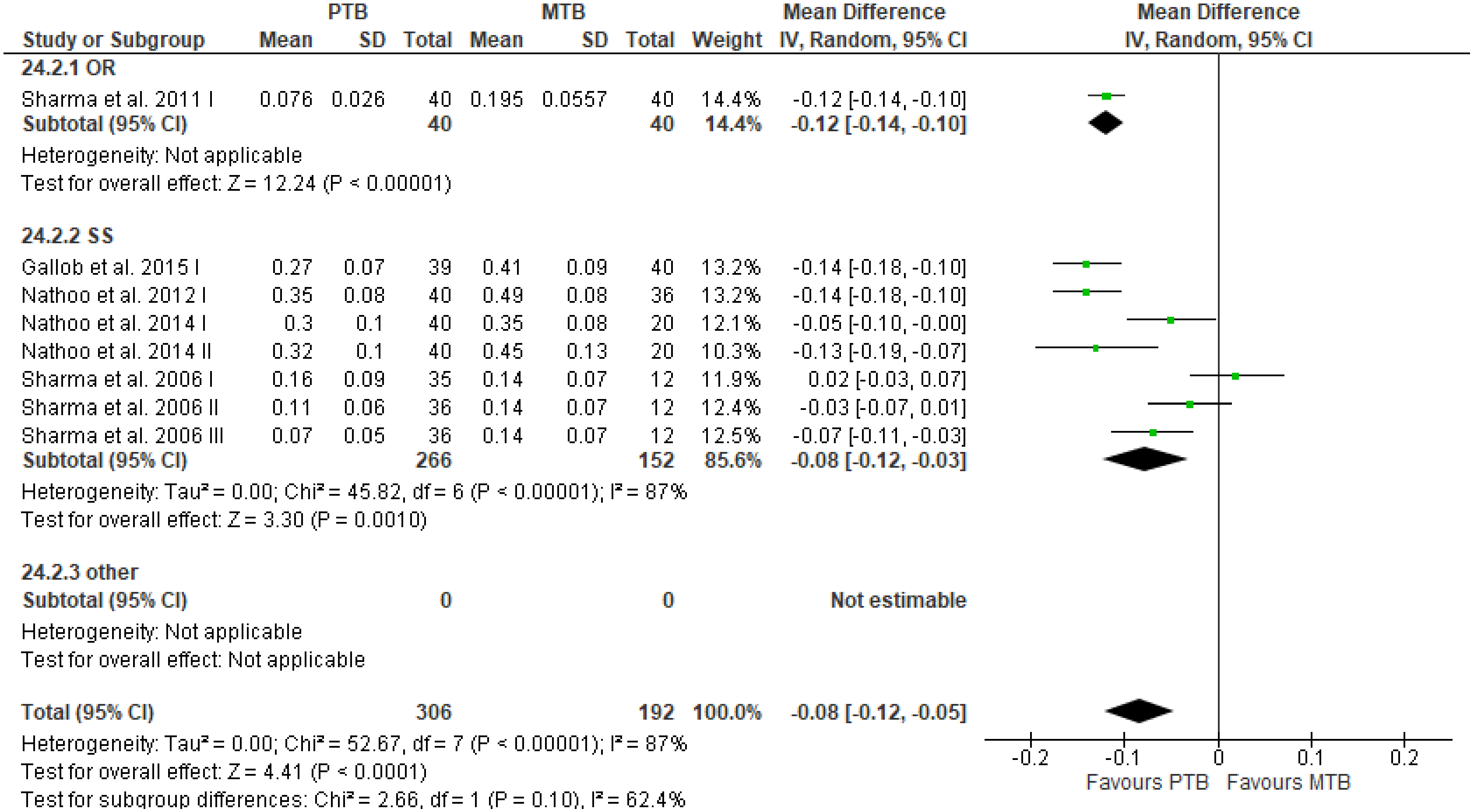

Appendix S13b

Forest plot of the random subanalysis for the SS mode of action for PTB compared to MTB at post-brushing in plaque scores on the RMNPI

Study or Subgroup	PTB Mean	PTB SD	PTB Total	MTB Mean	MTB SD	MTB Total	Weight	Mean Difference IV, Random, 95% CI
**24.2.1 OR**								
Sharma et al. 2011 I	0.076	0.026	40	0.195	0.0557	40	0.0%	-0.12 [-0.14, -0.10]
**Subtotal (95% CI)**			**0**			**0**		**Not estimable**
Heterogeneity: Not applicable								
Test for overall effect: Not applicable								
**24.2.2 SS**								
Gallob et al. 2015 I	0.27	0.07	39	0.41	0.09	40	15.2%	-0.14 [-0.18, -0.10]
Nathoo et al. 2012 I	0.35	0.08	40	0.49	0.08	36	15.1%	-0.14 [-0.18, -0.10]
Nathoo et al. 2014 I	0.3	0.1	40	0.35	0.08	20	14.2%	-0.05 [-0.10, -0.00]
Nathoo et al. 2014 II	0.32	0.1	40	0.45	0.13	20	12.5%	-0.13 [-0.19, -0.07]
Sharma et al. 2006 I	0.16	0.09	35	0.14	0.07	12	14.0%	0.02 [-0.03, 0.07]
Sharma et al. 2006 II	0.11	0.06	36	0.14	0.07	12	14.4%	-0.03 [-0.07, 0.01]
Sharma et al. 2006 III	0.07	0.05	36	0.14	0.07	12	14.6%	-0.07 [-0.11, -0.03]
**Subtotal (95% CI)**			**266**			**152**	**100.0%**	**-0.08 [-0.12, -0.03]**
Heterogeneity: Tau² = 0.00; Chi² = 45.82, df = 6 (P < 0.00001); I² = 87%								
Test for overall effect: Z = 3.30 (P = 0.0010)								
**Total (95% CI)**			**266**			**152**	**100.0%**	**-0.08 [-0.12, -0.03]**
Heterogeneity: Tau² = 0.00; Chi² = 45.82, df = 6 (P < 0.00001); I² = 87%								
Test for overall effect: Z = 3.30 (P = 0.0010)								
Test for subgroup differences: Not applicable								

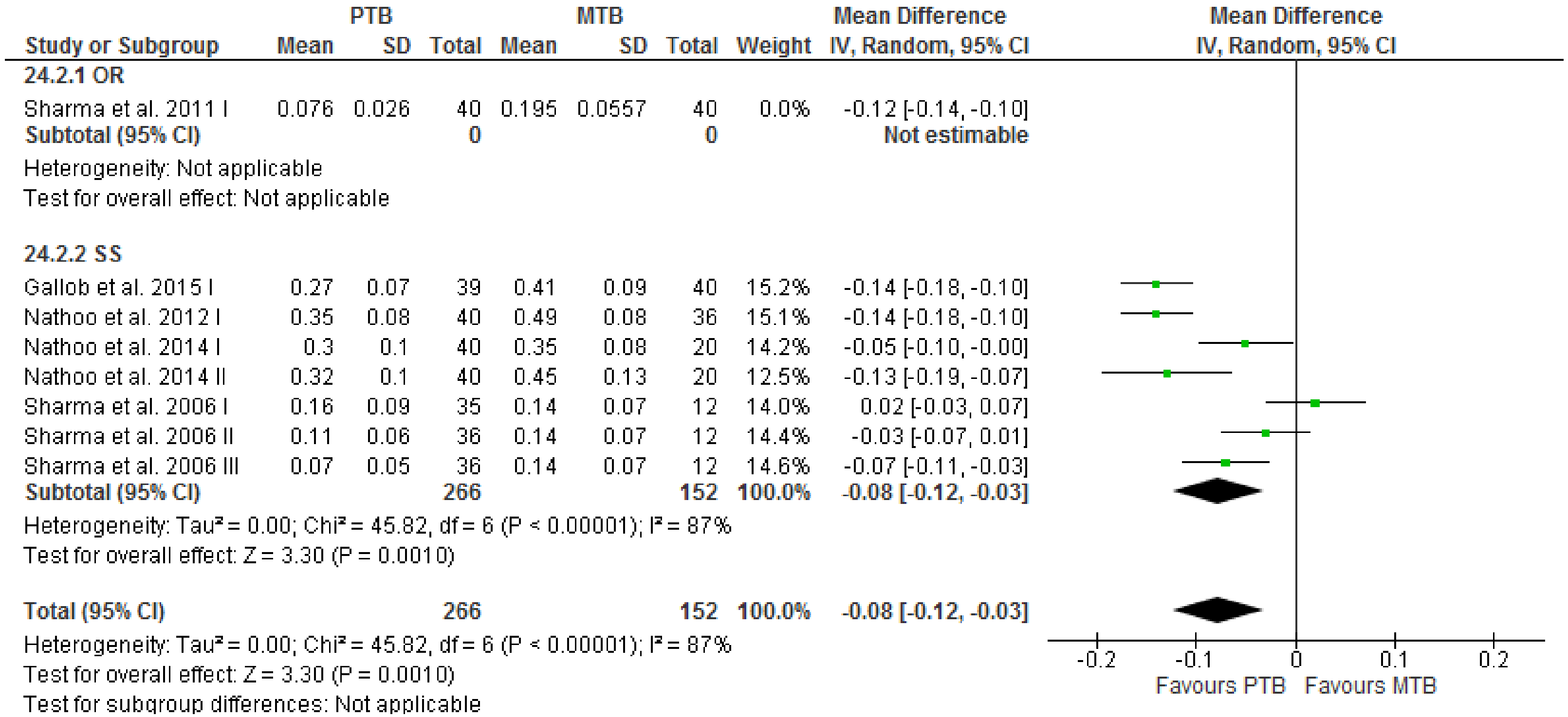

Appendix S13c

Forest plot of the random subanalysis for the SS mode of action by product (Colgate) for PTB compared to MTB at post-brushing in plaque scores on the RMNPI

	PTB			MTB				Mean Difference
Study or Subgroup	Mean	SD	Total	Mean	SD	Total	Weight	IV, Fixed, 95% CI
Nathoo et al. 2012 I	0.35	0.08	40	0.49	0.08	36	52.6%	-0.14 [-0.18, -0.10]
Nathoo et al. 2014 I	0.3	0.1	40	0.35	0.08	20	31.2%	-0.05 [-0.10, -0.00]
Nathoo et al. 2014 II	0.32	0.1	40	0.45	0.13	20	16.2%	-0.13 [-0.19, -0.07]
**Total (95% CI)**			**120**			**76**	**100.0%**	**-0.11 [-0.14, -0.08]**

Heterogeneity: Chi² = 9.34, df = 2 (P = 0.009); I² = 79%
Test for overall effect: Z = 8.28 (P < 0.00001)

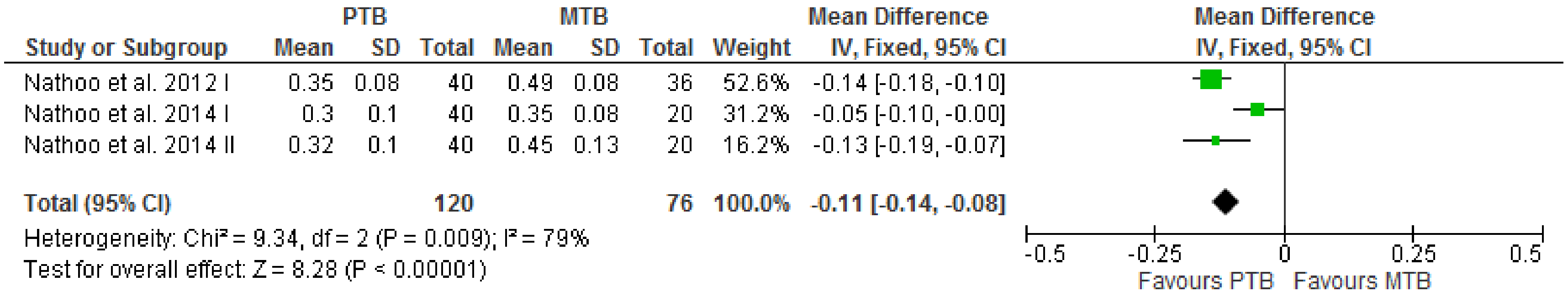

Appendix S14a

Forest plot of the overall analysis for PTB compared to MTB at the change in plaque scores on the RMNPI

Study or Subgroup	PTB Mean	PTB SD	PTB Total	MTB Mean	MTB SD	MTB Total	Weight	Mean Difference IV, Random, 95% CI
**24.3.1 OR**								
Sharma et al. 2011 I	-0.554	0.034	40	-0.43	0.034	40	14.6%	-0.12 [-0.14, -0.11]
**Subtotal (95% CI)**			**40**			**40**	**14.6%**	**-0.12 [-0.14, -0.11]**
Heterogeneity: Not applicable								
Test for overall effect: Z = 16.31 (P < 0.00001)								
**24.3.2 SS**								
Gallob et al. 2015 I	-0.39	0.06	39	-0.24	0.07	40	13.7%	-0.15 [-0.18, -0.12]
Nathoo et al. 2012 I	-0.4	0.08	40	-0.25	0.05	36	13.6%	-0.15 [-0.18, -0.12]
Nathoo et al. 2014 I	-0.41	0.07	40	-0.27	0.15	20	9.9%	-0.14 [-0.21, -0.07]
Nathoo et al. 2014 II	-0.43	0.09	40	-0.27	0.15	20	9.7%	-0.16 [-0.23, -0.09]
Sharma et al. 2006 I	-0.5	0.08	35	-0.51	0.06	12	12.5%	0.01 [-0.03, 0.05]
Sharma et al. 2006 II	-0.54	0.05	36	-0.51	0.06	12	13.0%	-0.03 [-0.07, 0.01]
Sharma et al. 2006 III	-0.59	0.05	36	-0.51	0.06	12	13.0%	-0.08 [-0.12, -0.04]
**Subtotal (95% CI)**			**266**			**152**	**85.4%**	**-0.10 [-0.15, -0.05]**
Heterogeneity: Tau² = 0.00; Chi² = 65.76, df = 6 (P < 0.00001); I² = 91%								
Test for overall effect: Z = 3.82 (P = 0.0001)								
**Total (95% CI)**			**306**			**192**	**100.0%**	**-0.10 [-0.14, -0.06]**
Heterogeneity: Tau² = 0.00; Chi² = 69.60, df = 7 (P < 0.00001); I² = 90%								
Test for overall effect: Z = 5.28 (P < 0.00001)								
Test for subgroup differences: Chi² = 0.91, df = 1 (P = 0.34), I² = 0%								

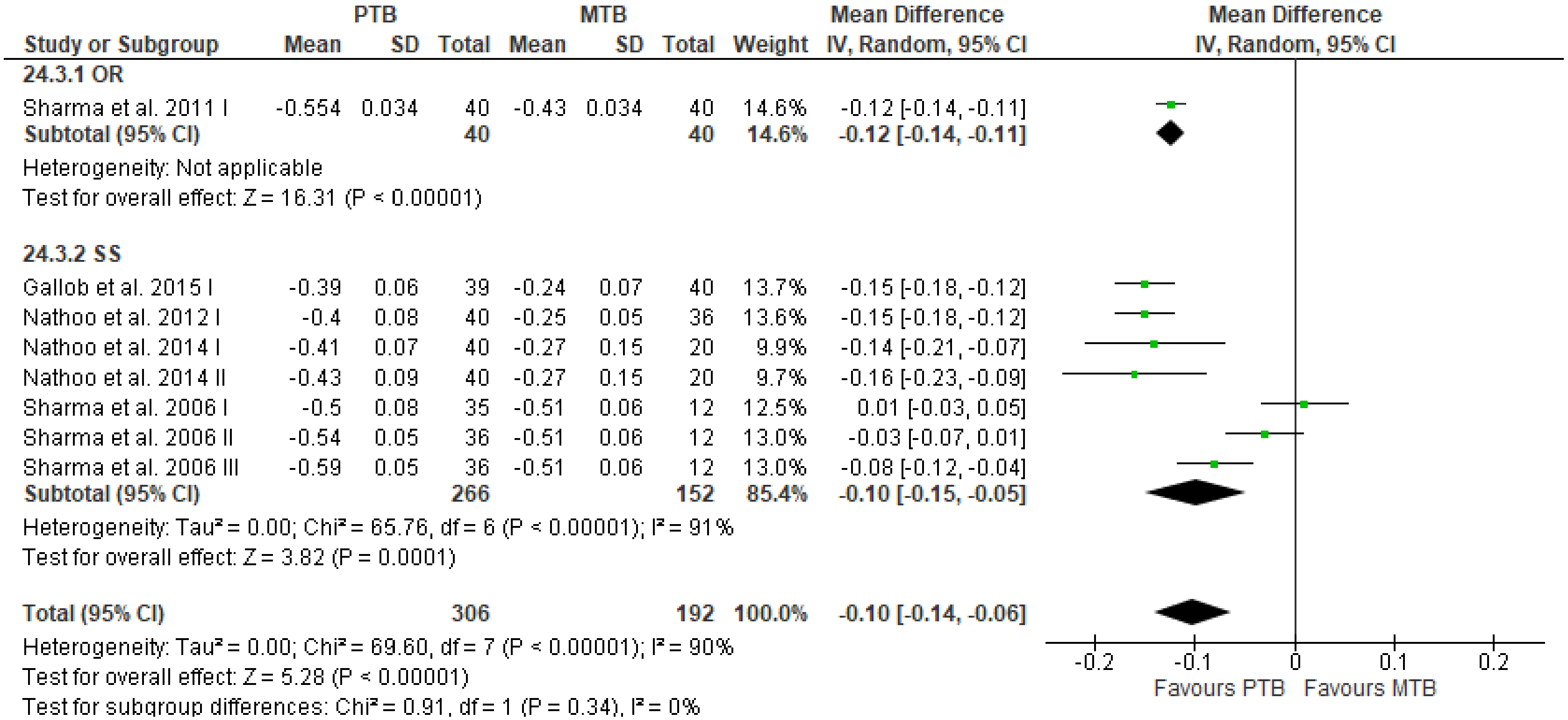

Appendix S14b

Forest plot of the random subanalysis for the SS mode of action for PTB compared to MTB at the change in plaque scores on the RMNPI

	PTB			MTB				Mean Difference
**Study or Subgroup**	**Mean**	**SD**	**Total**	**Mean**	**SD**	**Total**	**Weight**	**IV, Random, 95% CI**
**24.3.1 OR**								
Sharma et al. 2011 I	-0.554	0.034	40	-0.43	0.034	40	0.0%	-0.12 [-0.14, -0.11]
**Subtotal (95% CI)**			**0**			**0**		**Not estimable**
Heterogeneity: Not applicable								
Test for overall effect: Not applicable								
**24.3.2 SS**								
Gallob et al. 2015 I	-0.39	0.06	39	-0.24	0.07	40	15.5%	-0.15 [-0.18, -0.12]
Nathoo et al. 2012 I	-0.4	0.08	40	-0.25	0.05	36	15.4%	-0.15 [-0.18, -0.12]
Nathoo et al. 2014 I	-0.41	0.07	40	-0.27	0.15	20	12.5%	-0.14 [-0.21, -0.07]
Nathoo et al. 2014 II	-0.43	0.09	40	-0.27	0.15	20	12.3%	-0.16 [-0.23, -0.09]
Sharma et al. 2006 I	-0.5	0.08	35	-0.51	0.06	12	14.6%	0.01 [-0.03, 0.05]
Sharma et al. 2006 II	-0.54	0.05	36	-0.51	0.06	12	14.9%	-0.03 [-0.07, 0.01]
Sharma et al. 2006 III	-0.59	0.05	36	-0.51	0.06	12	14.9%	-0.08 [-0.12, -0.04]
**Subtotal (95% CI)**			**266**			**152**	**100.0%**	**-0.10 [-0.15, -0.05]**
Heterogeneity: Tau² = 0.00; Chi² = 65.76, df = 6 (P < 0.00001); I² = 91%								
Test for overall effect: Z = 3.82 (P = 0.0001)								
**Total (95% CI)**			**266**			**152**	**100.0%**	**-0.10 [-0.15, -0.05]**
Heterogeneity: Tau² = 0.00; Chi² = 65.76, df = 6 (P < 0.00001); I² = 91%								
Test for overall effect: Z = 3.82 (P = 0.0001)								
Test for subgroup differences: Not applicable								

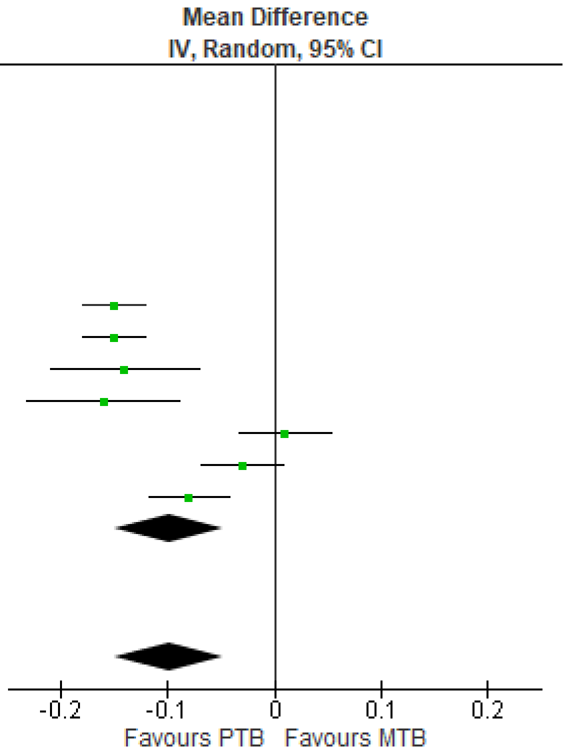

Appendix S14c

Forest plot of the random subanalysis for the SS mode of action by product (Colgate) for PTB compared to MTB at the change in plaque scores on the RMNPI

	PTB			MTB				Mean Difference
Study or Subgroup	Mean	SD	Total	Mean	SD	Total	Weight	IV, Fixed, 95% CI
Nathoo et al. 2012 I	-0.4	0.08	40	-0.25	0.05	36	73.7%	-0.15 [-0.18, -0.12]
Nathoo et al. 2014 I	-0.41	0.07	40	-0.27	0.15	20	13.6%	-0.14 [-0.21, -0.07]
Nathoo et al. 2014 II	-0.43	0.09	40	-0.27	0.15	20	12.7%	-0.16 [-0.23, -0.09]
**Total (95% CI)**			**120**			**76**	**100.0%**	**-0.15 [-0.18, -0.12]**

Heterogeneity: Chi² = 0.16, df = 2 (P = 0.93); I² = 0%
Test for overall effect: Z = 11.53 (P < 0.00001)

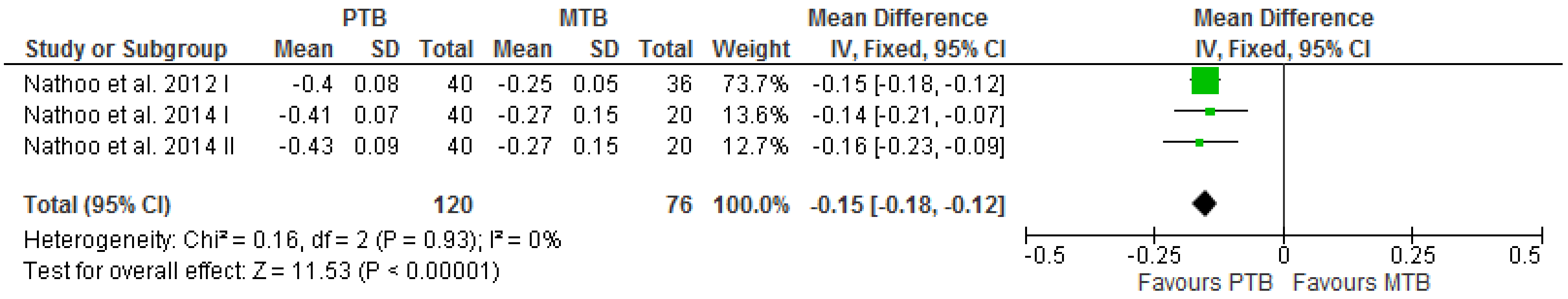

Supplement: Supplementary file 1 [file IDH-18-17-s001.pdf]
